# Trastuzumab for early-stage, HER2-positive breast cancer: a meta-analysis of 13 864 women in seven randomised trials

**DOI:** 10.1016/S1470-2045(21)00288-6

**Published:** 2021-08

**Authors:** Early Breast Cancer Trialists’ Collaborative group (EBCTCG), Rosie Bradley, Jeremy Braybrooke, Richard Gray, Robert Hills, Zulian Liu, Richard Peto, Lucy Davies, David Dodwell, Paul McGale, Hongchao Pan, Carolyn Taylor, Stewart Anderson, Richard Gelber, Luca Gianni, William Jacot, Heikki Joensuu, Alvaro Moreno-Aspitia, Martine Piccart, Michael Press, Edward Romond, Dennis Slamon, Vera Suman, Richard Berry, Clare Boddington, Mike Clarke, Christina Davies, Fran Duane, Vaughan Evans, Jo Gay, Lucy Gettins, Jon Godwin, Sam James, Hui Liu, Elizabeth MacKinnon, Gurdeep Mannu, Theresa McHugh, Philip Morris, Simon Read, Ewan Straiton, Yaochen Wang, John Crown, Evandro de Azambuja, Suzette Delaloge, Helena Fung, Charles Geyer, Marc Spielmann, Pinuccia Valagussa, Kathy Albain, Stewart Anderson, Rodrigo Arriagada, John Bartlett, Elizabeth Bergsten-Nordström, Judith Bliss, Etienne Brain, Lisa Carey, Robert Coleman, Jack Cuzick, Nancy Davidson, Lucia Del Mastro, Angelo Di Leo, James Dignam, Mitch Dowsett, Bent Ejlertsen, Prue Francis, Michael Gnant, Matthew Goetz, Pam Goodwin, Pat Halpin-Murphy, Dan Hayes, Catherine Hill, Reshma Jagsi, Wolfgang Janni, Sibylle Loibl, Eleftherios P Mamounas, Miguel Martín, Hirofumi Mukai, Valentina Nekljudova, Larry Norton, Yasuo Ohashi, Lori Pierce, Philip Poortmans, Vinod Raina, Daniel Rea, Meredith Regan, John Robertson, Emiel Rutgers, Tanja Spanic, Joseph Sparano, Guenther Steger, Gong Tang, Masakazu Toi, Andrew Tutt, Giuseppe Viale, Xiang Wang, Tim Whelan, Nicholas Wilcken, Norman Wolmark, David Cameron, Jonas Bergh, Kathleen I Pritchard, Sandra M Swain

## Abstract

**Background:**

Trastuzumab targets the extracellular domain of the HER2 protein. Adding trastuzumab to chemotherapy for patients with early-stage, HER2-positive breast cancer reduces the risk of recurrence and death, but is associated with cardiac toxicity. We investigated the long-term benefits and risks of adjuvant trastuzumab on breast cancer recurrence and cause-specific mortality.

**Methods:**

We did a collaborative meta-analysis of individual patient data from randomised trials assessing chemotherapy plus trastuzumab versus the same chemotherapy alone. Randomised trials that enrolled women with node-negative or node-positive, operable breast cancer were included. We collected individual patient-level data on baseline characteristics, dates and sites of first distant breast cancer recurrence and any previous local recurrence or second primary cancer, and the date and underlying cause of death. Primary outcomes were breast cancer recurrence, breast cancer mortality, death without recurrence, and all-cause mortality. Standard intention-to-treat log-rank analyses, stratified by age, nodal status, oestrogen receptor (ER) status, and trial yielded first-event rate ratios (RRs).

**Findings:**

Seven randomised trials met the inclusion criteria, and included 13 864 patients enrolled between February, 2000, and December, 2005. Mean scheduled treatment duration was 14·4 months and median follow-up was 10·7 years (IQR 9·5 to 11·9). The risks of breast cancer recurrence (RR 0·66, 95% CI 0·62 to 0·71; p<0·0001) and death from breast cancer (0·67, 0·61 to 0·73; p<0·0001) were lower with trastuzumab plus chemotherapy than with chemotherapy alone. Absolute 10-year recurrence risk was reduced by 9·0% (95% CI 7·4 to 10·7; p<0·0001) and 10-year breast cancer mortality was reduced by 6·4% (4·9 to 7·8; p<0·0001), with a 6·5% reduction (5·0 to 8·0; p<0·0001) in all-cause mortality, and no increase in death without recurrence (0·4%, –0·3 to 1·1; p=0·35). The proportional reduction in recurrence was largest in years 0–1 after randomisation (0·53, 99% CI 0·46 to 0·61), with benefits persisting through years 2–4 (0·73, 0·62 to 0·85) and 5–9 (0·80, 0·64 to 1·01), and little follow-up beyond year 10. Proportional recurrence reductions were similar irrespective of recorded patient and tumour characteristics, including ER status. The more high risk the tumour, the larger the absolute reductions in 5-year recurrence (eg, 5·7% [95% CI 3·1 to 8·3], 6·8% [4·7 to 9·0], and 10·7% [7·7 to 13·6] in N0, N1–3, and N4+ disease).

**Interpretation:**

Adding trastuzumab to chemotherapy for early-stage, HER2-positive breast cancer reduces recurrence of, and mortality from, breast cancer by a third, with worthwhile proportional reductions irrespective of recorded patient and tumour characteristics.

**Funding:**

Cancer Research UK, UK Medical Research Council.

## Introduction

Amplification of the *HER2* gene (also known as *ERBB2*) is present in 10–20% of tumours in patients with early-stage breast cancer and is associated with aggressive cancers and an increased risk of disease recurrence.^1^, ^2^ Trastuzumab, a humanised IgG1 monoclonal antibody that targets the extracellular domain of the HER2 protein, improves progression-free survival and overall survival when administered in combination with chemotherapy in HER2-positive metastatic breast cancer.^3^ Substantial benefits are also seen with trastuzumab added to chemotherapy in non-metastatic breast cancer.^4^, ^5^, ^6^, ^7^, ^8^, ^9^, ^10^ Hence, trastuzumab combined with chemotherapy is now a standard treatment for both metastatic and early-stage, HER2-positive breast cancer.

HER2 status is most reliably ascertained by in-situ hybridisation using probes to label the number of gene copies of *HER2* and the chromosome 17 centromere (CEP17), with most trials accepting a HER2 to CEP17 ratio of at least 2·0 as positive.^11^ Alternatively, immunohistochemical staining of the HER2 protein can be used, with 3+ on a scale of 0, 1+, 2+, and 3+ considered as positive, and in-situ hybridisation used to confirm HER2 status for tumours that are 2+.

However, it is not clear how the level of HER2 amplification, tumour characteristics, in particular oestrogen receptor (ER) status, or other patient risk factors influence the magnitude and duration of treatment benefit. Trastuzumab is expensive and can be associated with cardiac toxicity, particularly when administered with anthracyclines.^3^ In this meta-analysis, we aimed to evaluate the long-term benefits and risks of trastuzumab on breast cancer recurrence and cause-specific mortality, and whether these effects vary by patient characteristics or types of tumour.

**Research in context**

**Evidence before this study**

Amplification of the *HER2* gene is present in 10–20% of tumours in patients with early-stage breast cancer and is associated with aggressive cancers and an increased risk of disease recurrence. The Early Breast Cancer Trialists’ Collaborative Group (EBCTCG)'s ongoing extensive searches of bibliographic databases, including MEDLINE, Embase, the Cochrane Library, and meeting abstracts, up to March 31, 2020, identified seven trials comparing chemotherapy plus trastuzumab, a humanised IgG1 monoclonal antibody that targets the extracellular domain of the HER2 protein, with the same chemotherapy alone for early-stage, HER2-positive breast cancer. Individual trials reported substantial benefits with trastuzumab; however, cardiac toxicity was increased and it was unclear how the level of HER2 amplification, tumour characteristics (eg, oestrogen receptor [ER] status), or other patient risk factors influence the magnitude and duration of benefit.

**Added value of this study**

This collaborative meta-analysis collated, checked, and analysed individual patient-level data from 13 864 women in seven randomised controlled trials. Trastuzumab reduced the rate of breast cancer recurrence by 34% and of breast cancer mortality by 33%, compared with chemotherapy alone. The average absolute reduction in the 10-year risk of breast cancer recurrence was 9·0%, with a 6·4% reduction in the 10-year risk of dying from breast cancer, and no increase in deaths unrelated to breast cancer. The proportional reductions in recurrence were similar for ER-positive and ER-negative tumours, and did not differ significantly by other patient or tumour characteristics, including the level of HER2 amplification, for patients considered to be HER2-positive.

**Implications of all the available evidence**

Adding trastuzumab to chemotherapy for patients with early-stage, HER2-positive breast cancer reduces recurrence of and mortality from breast cancer by a third, with worthwhile proportional benefits irrespective of recorded patient and tumour characteristics.

## Methods

### Study design and participants

Methods of identifying trials, data collection, checking, analysis, and presentation for this collaborative meta-analysis of individual patient-level data are as described in previous EBCTCG reports,^12^, ^13^, ^14^, ^15^ and conform to the Preferred Reporting Items for Systematic Reviews and Meta-Analyses (Individual Patient Data).^16^

Trials were eligible if they began before Jan 1, 2010, and randomly assigned women with node-negative or node-positive, operable breast cancer to either chemotherapy (adjuvant or neoadjuvant) plus trastuzumab or to the same chemotherapy alone. Trials of trastuzumab added to other biological therapies (eg, trials comparing lapatinib, pertuzumab, or neratinib with or without trastuzumab) were not eligible. The lead investigators of all identified, eligible trials were asked during 2016–19 to supply information for each individual patient on randomisation date; allocated treatment; age; menopausal status; body-mass index (BMI); tumour diameter, grade, histology, and spread to loco-regional lymph nodes; ER, progesterone receptor (PR), and HER2 receptor test scores; cell proliferation (Ki-67); dates and sites of first distant breast cancer recurrence and any previous local recurrence or second primary cancer; and the date and underlying cause of death.

Primary outcomes were any recurrence of invasive breast cancer (distant, loco-regional, or new primary in the contralateral breast), breast cancer mortality, death without recurrence, and all-cause mortality. Prespecified primary subgroup investigations were by site of recurrence; concurrent or sequential trastuzumab and chemotherapy; level of HER2 amplification; age; ER status; PR status; nodal status; tumour diameter, grade, histology (ie, ductal or lobular); BMI; proliferation index (Ki-67 <10%, 10–19%, or ≥20%); and follow-up period (years 0–1, 2–4, 5–9, ≥10).

### Statistical analysis

Statistical methods for checking and analysing data are described in previous EBCTCG reports,^12^, ^13^, ^14^, ^15^ and in the statistical analysis plan (appendix). Time-to-event analyses were stratified by age, ER status, trial, and, except for studies including neoadjuvant trastuzumab, nodal status. Each analysis compared all women randomly assigned, regardless of treatment compliance (yielding intention-to-treat analyses). Log-rank statistics were used to estimate the ratio of the annual event rates in the trastuzumab and control groups and its confidence interval; 95% CIs were used for meta-analyses and 99% CIs were used for individual trials or subgroups. Breast cancer mortality rate ratios (RRs) were estimated by subtracting log-rank statistics for mortality without recurrence from those of overall mortality, which avoids the need to identify which deaths after recurrence were from breast cancer.^14^ Forest plots and Kaplan-Meier graphs describe the separate trials and their combined results, and tests for heterogeneity^13^ explore whether proportional risk reductions vary by trial, or by patient or tumour-related characteristics. Sensitivity analyses to investigate the effect of crossover to trastuzumab censored all women in the HERA trial on the date when crossover to trastuzumab was offered to control patients. Statistical analyses used in-house FORTRAN programmes.

### Role of the funding source

The funders of the study had no role in study design, data collection, data analysis, data interpretation, or writing of the report.

## Results

Individual patient-level data were obtained from all seven identified, relevant trials (table),^4^, ^5^, ^6^, ^7^, ^8^, ^9^, ^10^ providing data for 13 864 women with early-stage, HER2-positive breast cancer who had been randomly assigned to receive chemotherapy plus trastuzumab or chemotherapy alone between February, 2000, and December, 2005. Of these women, 3685 (26·6%) had breast cancer recurrence. 2738 (19·7%) deaths occurred, of which 347 (12·7%) were from causes unrelated to breast cancer and without recorded disease recurrence. Median follow-up was 10·7 years (IQR 9·5–11·9). Trial inclusion criteria were HER2-positive tumours, with most trials requiring that these should be either lymph node-positive or high-risk if node-negative (generally defined as grade 3, tumour >1 cm if ER-negative or >2 cm if ER-positive; table). HER2 status was based on local immunohistochemistry or fluorescence in-situ hybridisation testing, with some studies requiring central laboratory confirmation.

**Table:** Baseline patient characteristics

	**Years of recruitment**	**Number of patients**	**Key participant eligibility criteria**	**Median age (IQR), years**	Nodal status*	**Tumour size**	**Tumour grade**	**ER status**	**HER2:CEP17 ratio**	**Median follow-up, years (IQR)**
FinHER trial (Joensuu et al, 2009)^6^	2000–03	231	Aged ≤65 years; HER2-positive; node-positive or node-negative cancer with tumour size >2 cm and negative staining for PR	50 (25–65)	16% N0; 52% N1–3; 32% N4+	7% T1a+b; 28% T1c; 58% T2; 6% T3 or T4; 1% unknown	2% well differentiated; 31% moderate; 64% poor; 3% unknown	53% ER-negative; 47% ER-positive	No data	10·7 (10·1–11·4)
NSABP trial B-31 (Romond et al, 2005)^4^	2000–05	2119	Aged ≥18 years; HER2-positive; node-positive disease	49 (22–78)	57% N1–3; 43% N4+	8% T1a+b; 32% T1c; 50% T2; 9% T3 or T4; 1% unknown	2% well differentiated; 29% moderate; 68% poor; 1% unknown	47% ER-negative; 53% ER-positive	No data	9·4 (8·1–10·8)
NCCTG trial N9831 (Romond et al, 2005)^4^	2000–05	3505	Aged ≥18 years; HER2-positive; node-positive or high-risk node-negative disease defined as tumour size >2 cm and positive ER or PR, or tumour size >1 cm and negative ER and PR	50 (19–82)	13% N0; 40% N1–3; 39% N4+; 8% unknown	7% T1a+b; 32% T1c; 52% T2; 9% T3 or T4	2% well differentiated; 27% moderate; 70% poor; 1% unknown	47% ER-negative; 53% ER-positive	0–1·9: 8%; 2·0–3·4: 6%; 3·5–4·9: 5%; 5·0–7·4: 16%; 7·5–9·9: 17%; ≥10·0: 20%; unknown: 28%	12·8 (11·3–14·2)
HERA trial (Piccart-Gebhart et al, 2005)^5^	2001–05	5099	Aged ≥18 years; HER2-positive; node-positive disease (irrespective of pathological tumour size) or node-negative disease if tumour size >1 cm on pathological examination	49 (18–79)	32% N0; 29% N1–3; 28% N4+; 11% unknown	5% T1a+b; 34% T1c; 44% T2; 5% T3 or T4; 12% unknown	2% well differentiated; 32% moderate; 61% poor; 5% unknown	51% ER-negative; 49% ER-positive	0–1·9: 1%; 2·0–3·4: 9%; 3·5–4·9: 12%; 5·0–7·4: 17%; 7·5–9·9: 9%; ≥10·0: 6%; unknown: 46%	11·0 (10·1–11·5)
PACS 04 trial (D'Hondt et al, 2019)^8^	2001–04	528	Aged 18–65 years; HER2-positive; node-positive disease	49 (22–65)	58% N1–3; 42% N4+	7% T1a+b; 38% T1c; 48% T2; 7% T3 or T4	3% well differentiated; 31% moderate; 65% poor; 1% unknown	44% ER-negative; 56% ER-positive	No data	9·6 (8·0–10·1)
BCIRG 006 trial (Slamon et al, 2011)^9^	2001–04	2147	Aged 18–70 years; HER2-positive; node-positive or high-risk node-negative disease defined as invasive adenocarcinoma with either 0 (pN0) among a minimum of six resected lymph nodes or negative sentinel node biopsy (pN0) and at least one of the following: tumour size >2 cm; negative ER, PR, or both; grade 2–3; or aged <35 years	49 (22–74)	29% N0; 38% N1–3; 33% N4+	7% T1a+b; 32% T1c; 54% T2; 6% T3 or T4	2% well differentiated; 29% moderate; 65% poor; 4% unknown	51% ER-negative; 49% ER-positive	2·0–3·4: 10%; 3·5–4·9: 11%; 5·0–7·4: 26%; 7·5–9·9: 21%; ≥10·0: 29%; unknown: 3%	10·5 (8·5–10·7)
NOAH trial (Gianni et al, 2010)^7^	2002–05	235	Aged ≥18 years; HER2-positive; suitable for neoadjuvant chemotherapy	51 (25–81)	15% N0; 85% N+	No data	No data	69% ER-negative; 31% ER-positive	No data	5·8 (4·8–6·7)

All trials administered chemotherapy and trastuzumab after surgery, except for the NOAH trial, which gave trastuzumab with neoadjuvant chemotherapy in addition to giving trastuzumab after surgery. ER=oestrogen receptor. PR=progesterone receptor.

* Nodal status recorded at the time of surgery, apart from in the NOAH trial, in which it was determined before neoadjuvant therapy.

Sequential 12-week anthracycline followed by 12-week taxane chemotherapy regimens were used in the NSABP trial B-31 (2119 [15·3%] of 13 864 patients),^4^ NCCTG trial N9831 (3505 [25·3%]),^4^ and BCIRG 006 trial (2147 [15·5%]).^9^ Patients in the NOAH trial (235 [1·7%] patients)^7^ were given concurrent taxane and anthracycline, then taxane alone followed by cyclophosphamide–methotrexate–fluorouracil. FinHER (231 [1·7%] patients)^6^ included a randomisation to docetaxel or vinorelbine before anthracyclines, and PACS 04 (528 [3·8%] patients)^8^ randomly assigned patients to fluorouracil–epirubicin–cyclophosphamide or to epirubicin plus docetaxel. The choice of chemotherapy was not mandated in the HERA study (5099 [36·8%] patients), with 94% receiving anthracyclines and 26% receiving a taxane in addition to an anthracycline.^5^ BCIRG 006 included a non-anthracycline (docetaxel–carboplatin–trastuzumab) group, which was excluded from the main analyses as there was no control group receiving the same chemotherapy. Comparing the docetaxel–carboplatin–trastuzumab group with the anthracycline and docetaxel control group would have shown similar benefits from adding trastuzumab as those in the included trials (appendix p 20). In all trials, endocrine therapy was recommended in hormone receptor-positive disease.

Except for the NOAH trial,^7^ chemotherapy plus trastuzumab was administered after surgery. In HERA, PACS-04, and one group in NCCTG trial N9831, trastuzumab was commenced after completion of all chemotherapy. In other trials, trastuzumab was started after any anthracycline courses and given concurrently with taxane (or vinorelbine for 120 [52%] of FinHER patients) chemotherapy. Duration of treatment was 12 months in all trials except for FinHER,^6^ which tested 9 weeks of trastuzumab. The HERA trial also included a 24-month trastuzumab treatment group,^5^ which was included in the primary analyses of trastuzumab (any duration) versus no trastuzumab. Thus, mean scheduled treatment duration across all trials was 14·4 months. Following presentation of early analyses in June, 2005, 884 (52·1%) patients in the control group of HERA^10^ and 382 (36·0%) patients in the control group of NSABP trial B-31^4^ crossed over to receive trastuzumab (at median periods of 23 months from randomisation for HERA and 17 months for NSABP trial B-31). There were few crossovers in the other trials.

**Figure 1:**
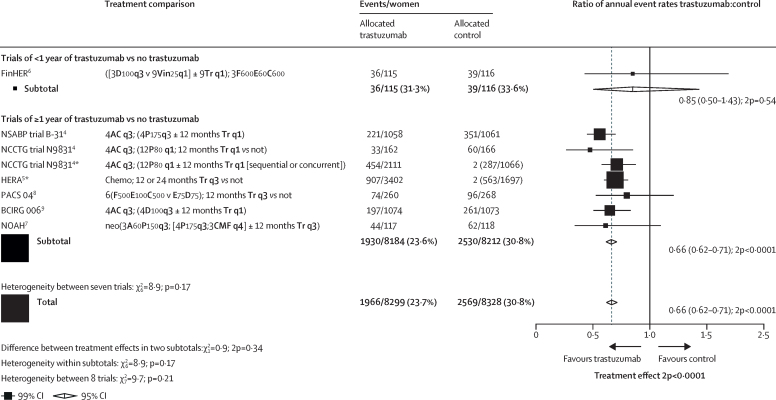
Recurrence (distant, local, or contralateral) in trials testing trastuzumab versus control A=doxorubicin (adriamycin). C=cyclophosphamide. D=docetaxel. E=epirubicin. F= fluorouracil. M=methotrexate. P=paclitaxel. Tr=trastuzumab. Vin=vinorelbine. q1=once weekly. q3=once every three weeks. q4=once every four weeks. *For balance, control patients in three-way trials or trial strata count twice in subtotals and in final total of events or patients.

**Figure 2:**
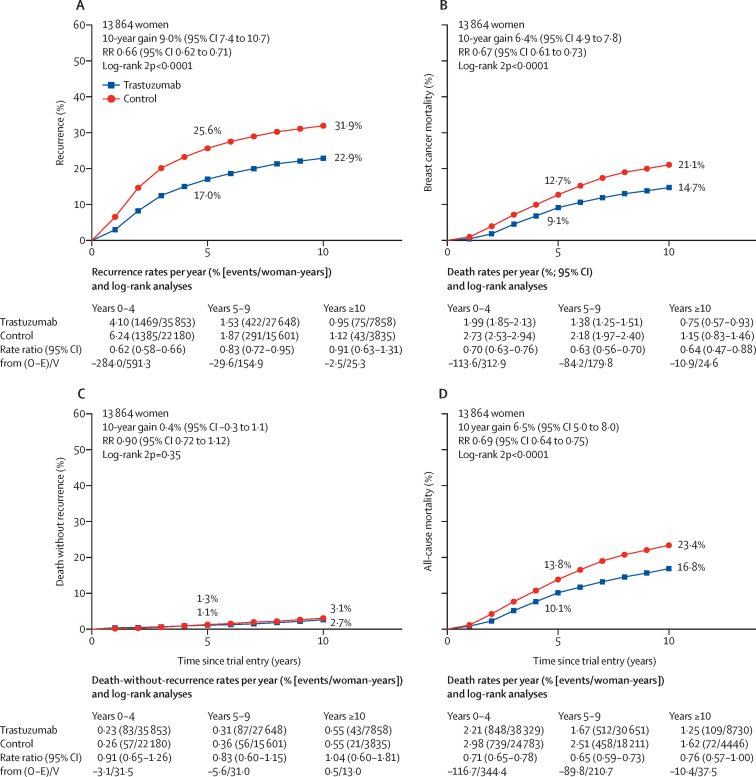
Effect of trastuzumab versus control on recurrence and mortality 10-year cumulative risk of any recurrence (ie, distant, local, or contralateral; A), breast cancer mortality (B), death without any recurrence (C), and death from any cause (D). Breast cancer mortality rates calculated by total rate (events/woman-years) – rate in women without recurrence. Error bars are 95% CIs. O–E=observed minus expected. RR=rate ratio. V=variance of O–E.

**Figure 3:**
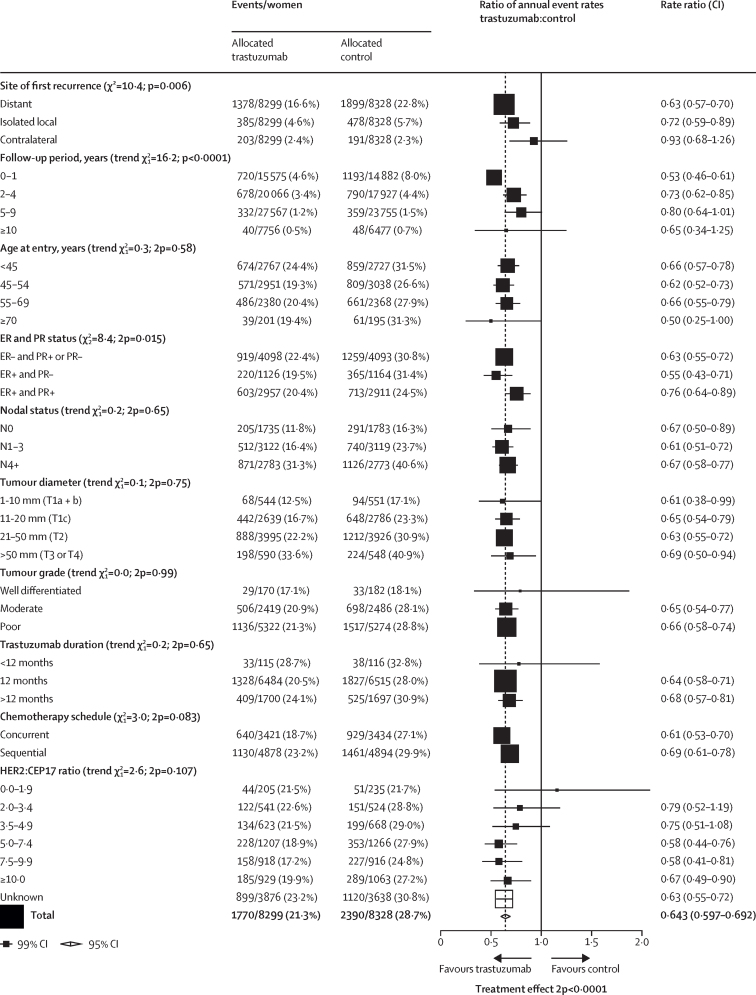
Subgroup analyses of the effect of trastuzumab All analyses, except for site of first recurrence, include any loco-regional or distant recurrence but exclude contralateral disease. ER=oestrogen receptor. PR=progesterone receptor.

Figure 1 shows the year started, study name, treatment comparison, log-rank statistics, and the ratio of annual event rates for each trial. Pooled analysis of all trials found a highly significant reduction in recurrence (RR 0·66, 95% CI 0·62 to 0·71; p<0·0001; figure 1) and breast cancer mortality (0·67, 0·61 to 0·73; p<0·0001; appendix p 7) with chemotherapy plus trastuzumab compared with chemotherapy alone. There was no significant heterogeneity between the seven trial results for recurrence or breast cancer mortality (p=0·21). The average absolute reduction in 10-year risk of recurrence was 9·0% (95% CI 7·4 to 10·7; p<0·0001) with a 6·4% (4·9 to 7·8; p<0·0001) reduction in 10-year breast cancer mortality, a 6·5% (5·0 to 8·0; p<0·0001) reduction in all-cause mortality, and no increase in death without recurrence (0·4%, –0·3 to 1·1; p=0·35; figure 2; appendix pp 3–11). Subgroup analyses of breast cancer recurrence (local or distant, excluding contralateral), distant recurrence, and breast cancer mortality are shown in figure 3 and the appendix (pp 15–17). Trastuzumab reduced distant recurrence (RR 0·63, 99% CI 0·57 to 0·70) and local recurrence (0·72, 0·59 to 0·89), but not contralateral breast cancer incidence (0·93, 0·68 to 1·26; figure 3). There was little or no reduction in the incidence of brain metastases as the first site of distant recurrence (RR 0·91, 95% CI 0·73 to 1·13; p=0·40), a significantly lesser effect of trastuzumab than on distant recurrence at other sites (0·60, 0·55 to 0·65; p<0·0001; appendix p 18).

**Figure 4:**
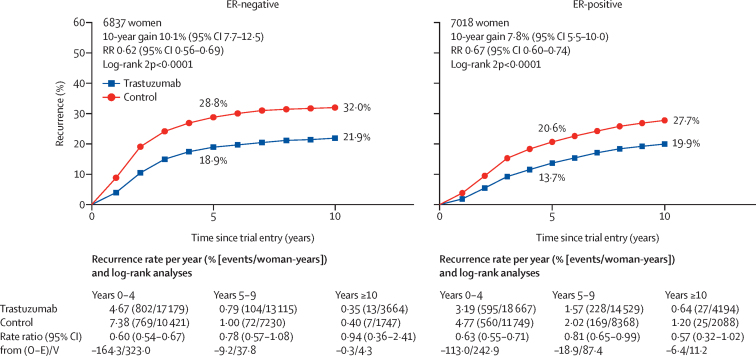
Effect of trastuzumab versus control on recurrence by ER status Recurrence analyses include any loco-regional or distant recurrence, but exclude contralateral disease. Error bars are 95% CIs. ER=oestrogen receptor. O–E=observed minus expected. RR=rate ratio. V=variance of O–E.

The largest proportional reduction in recurrence was in years 0–1 (RR 0·53, 99% CI 0·46–0·61) with smaller proportional reductions in years 2–4 (0·73, 0·62–0·85) and years 5–9 (0·80, 0·64–1·01), and little follow-up beyond year 10 (figure 3). There were similar proportional reductions in risk of recurrence for ER-negative and ER-positive cancers (RR 0·62, 95% CI 0·56–0·69 *vs* 0·67, 0·60–0·74; figure 4). The recurrence rates in the first 2 years were higher for ER-negative than for ER-positive cancers (appendix p 12), but were higher for ER-positive than for ER-negative in years 5–9. Hence, the average absolute reductions in 10-year recurrence risk with trastuzumab were similar for women with ER-negative (10·1%, 95% CI 7·7–12·5) and ER-positive disease (7·8%, 5·5–10·1; figure 4). The average absolute reductions in 10-year breast cancer mortality were 6·9% (4·8–9·1) for ER-negative tumours and 5·1% (3·2–7·0) for ER-positive tumours (appendix p 13). The proportional reductions in recurrence were greater in ER-positive, PR-negative tumours than for ER-positive, PR-positive tumours (RR 0·55, 95% CI 0·45–0·67 *vs* 0·76, 0·67–0·86; appendix p 14).

The proportional reduction in recurrence did not vary by age, BMI, duration of trastuzumab treatment, or traditional histopathological features (including tumour size, nodal status, tumour grade, or histological subtype), although there were relatively few women older than 70 years, or with low-grade or lobular cancers (figure 3). In the few women with Ki-67 scores, there was no indication that efficacy varied with increasing proliferation index (figure 3).

In three trials, individual patient HER2 to CEP17 ratios were available from central pathology fluorescence in-situ hybridisation tests, with tumours with a ratio of at least 2·0 deemed to be HER2-positive. Figure 3 shows the reductions in risk of recurrence subdivided by finer categories of the HER2 to CEP17 ratio, suggesting no effect in the few patients with HER2 to CEP17 ratios of less than 2·0, and no significant trend of increasing benefit with increasing HER2 to CEP17 ratios above this threshold.

**Figure 5:**
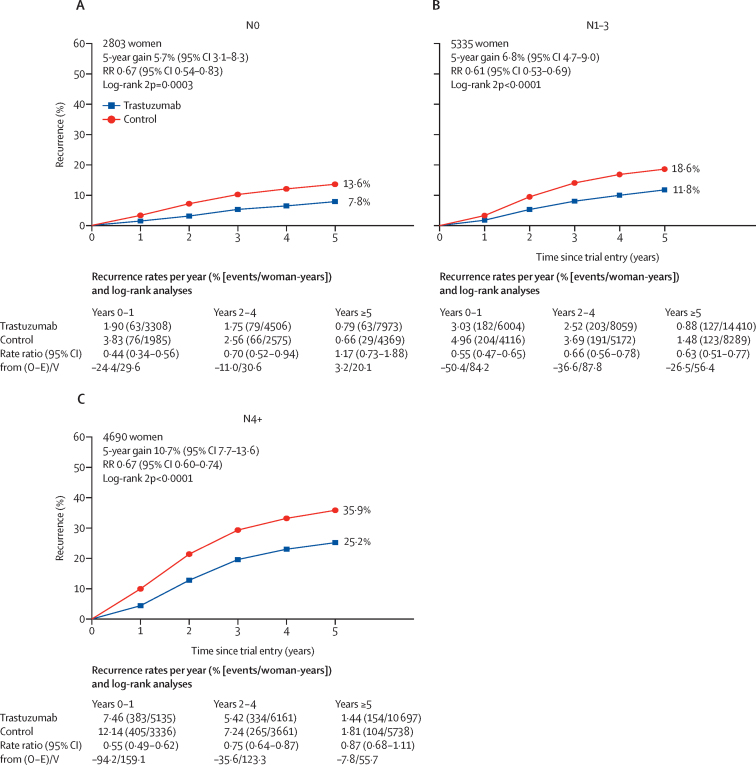
Effect of trastuzumab versus control on recurrence by nodal status Recurrence analyses include any loco-regional or distant recurrence, but exclude contralateral disease. Error bars are 95% CIs. O–E=observed minus expected. RR=rate ratio. V=variance of O–E.

Figure 5 shows the 5-year risk of recurrence in different nodal status groups. Although the proportional reduction was similar across groups, the absolute reduction in 5-year risk was greatest in patients with more involved nodes (5·7%, 95% CI 3·1–8·3 in N0; 6·8%, 4·7–9·0 in N1–3; and 10·7%, 7·7–13·6 in ≥N4 disease) because of their higher absolute risk of recurrence.

The reduction in recurrence in trials that administered trastuzumab concurrently with chemotherapy did not differ significantly from that in trials where it was given sequentially after completion of chemotherapy (RR 0·61, 99% CI 0·53–0·70 for concurrent trastuzumab *vs* 0·69, 0·61–0·78 for sequential trastuzumab; heterogeneity p=0·083; figure 3). In HERA, the largest sequential treatment trial, trastuzumab was offered to control patients, which might have attenuated the benefits of trastuzumab. In sensitivity analyses censoring all women in the HERA trial on the date when crossover to trastuzumab was offered to control patients, the average absolute improvement in 10-year recurrence risk with trastuzumab increased from 9·0% to 10·6% (appendix p 21). To minimise the effects of crossover, we compared the recurrence reductions seen with concurrent versus sequential trastuzumab in years 0–4 from randomisation, which showed a significantly larger reduction with concurrent treatment than with sequential treatment (p=0·02; appendix p 22). This indirect comparison is supported by the direct randomised comparison between concurrent and sequential trastuzumab in the NCCTG trial N9831, in which there were fewer distant recurrences (RR 0·78, 95% CI 0·63–0·97; p=0·026) and breast cancer deaths (0·76, 0·60–0·96, p=0·023) with concurrent trastuzumab than with sequential trastuzumab (appendix pp 23–24).

Overall, there was no apparent increase in deaths without breast cancer recurrence in the trastuzumab group (RR 0·90, 95% CI 0·72–1·12; p=0·35; appendix p 19). Similarly, there was no apparent increase in cardiovascular mortality (RR 1·23, 95% CI 0·73–2·06; p=0·44) or in deaths from new cancers at sites other than the breast (0·79, 0·54–1·17; p=0·24). However, there were more deaths unrelated to breast cancer in the first year with chemotherapy plus trastuzumab than with chemotherapy alone (0·37% [31/8299] *vs* 0·16% [13/8328]; RR 2·15, 95% CI 1·11–4·14; p=0·023; appendix p 8). No particular cause of death explained this excess (appendix p 19). Of the 414 deaths without recorded recurrence, 72 (17%) were from unknown causes; however, these were unrelated to treatment allocation or TNM stage (appendix p 25), so are included with the deaths unrelated to breast cancer. The risk of death unrelated to breast cancer in the first year following randomisation appeared to be no greater in trials with concurrent chemotherapy plus trastuzumab (0·34% [11/3251] with trastuzumab *vs* 0·22% [7/3195] with control) than in trials with sequential chemotherapy followed by trastuzumab (0·43% [20/4607] with trastuzumab *vs* 0·13% [6/4468] with control; appendix p 19).

Patient-level data on cardiac toxicity were available from just one trial; the cardiac and non-cardiac toxicity reported in individual trial publications is summarised in the appendix (pp 26–29). The incidence of congestive heart failure and asymptomatic decrease in left ventricular ejection fraction, usually resulting in treatment discontinuation, was consistently higher in the trastuzumab group than in the control group, but the proportion of patients affected was low (appendix pp 26–29). Few fatal toxic events were reported with no excess in the trastuzumab group (appendix pp 26–29).

## Discussion

This meta-analysis substantiates that—for patients with operable HER2-positive breast cancer—the addition of trastuzumab to chemotherapy further reduces recurrence of, and mortality from, breast cancer during the first decade of follow-up by about a third. For the patient population in the trials in this analysis, this finding translates, on average, into a 10-year absolute reduction of 9·0% in recurrence and of 6·4% in breast cancer mortality, compared with chemotherapy alone. These reductions were achieved despite substantial crossover from control to trastuzumab in two trials; therefore, the benefits might have been larger with perfect compliance. The greatest effect was on distant recurrence, although local recurrence was also reduced; however, there was no apparent effect on the incidence of new contralateral breast cancers.

Subgroup analyses by conventional pathological features indicated similar proportional reductions in recurrence, irrespective of nodal status, tumour grade, tumour diameter, and histological subtype. Trastuzumab produced similar proportional reductions in recurrence of ER-positive and ER-negative disease, as did chemotherapy alone in previous meta-analyses.^12^, ^17^ Similar to HER2-negative disease, the risk of early (years 0–4) recurrence was higher for HER2-positive, ER-negative tumours than for HER2-positive, ER-positive tumours. However, late (years 5–9) recurrences are more likely in ER-positive than in ER-negative disease, even though patients with ER-positive disease were scheduled to receive at least 5 years of endocrine therapy.^18^

As the proportional reductions in different subgroups were similar, there were greater absolute reductions in risk among patients with node-positive disease than among those with node-negative disease because of their higher absolute risk of recurrence. Nevertheless, the absolute benefits were substantial, even in the patients with node-negative disease entered into these trials, suggesting appreciable benefits from the addition of trastuzumab to chemotherapy for all patients with HER2-positive disease fit enough to receive systemic treatment.

Only three studies included central fluorescence in-situ hybridisation testing and, in these, benefits from trastuzumab did not increase with increasing HER2 to CEP17 ratio above the conventional cutoff of 2·0. There was no apparent benefit in tumours with HER2 to CEP17 ratios below the 2·0 threshold for HER2 positivity, consistent with the reduced efficacy of trastuzumab in low HER2 tumours in the NSABP trial B-47;^19^ however, there were not enough patient numbers to assess whether, for example, 1·8 or 2·2 might be a better cutoff for HER2 positivity than might 2·0.

Starting trastuzumab concurrently with the taxane component of chemotherapy seemed at least as efficacious as giving it sequentially (ie, starting only after completing all chemotherapy), which is consistent with data suggesting that giving trastuzumab concurrently with chemotherapy is synergistic.^20^ Sequential administration does allow for assessment of cardiac function (an eligibility requirement for HERA) following chemotherapy; however, in patients for whom cardiac risk is not a major concern, giving trastuzumab concurrently with the taxane cycles seems to be advisable given that benefits emerge early: the biggest impact on recurrence was seen in the first 2 years after randomisation and there was no apparent increase in toxicity with concurrent taxane chemotherapy and trastuzumab, compared with sequential treatment.

We found no overall increase in mortality from causes unrelated to breast cancer. Nevertheless, there were more deaths without recurrence in the first year after randomisation with trastuzumab than with control, which was not explained by administering trastuzumab concurrently with chemotherapy. This finding could well be due to chance, as the difference is spread over several different causes. However, even if the apparent excess risk is real, the excess number of these deaths was 30 times smaller than the number of deaths from breast cancer prevented. Despite the well documented adverse effects of trastuzumab on cardiac function, the number of deaths from cardiovascular causes was low and not significantly higher in patients treated with chemotherapy plus trastuzumab than in those treated with chemotherapy alone, neither overall nor in the first year. Longer follow-up of these trials is needed to evaluate safety more than 10 years after treatment. Individual patient-level data on non-fatal toxicity, including cardiac morbidity, was not available for this meta-analysis, but data from individual trial reports indicate that serious toxicity is rare, even though most trials administered trastuzumab after anthracycline chemotherapy regimens. Although more effective, this type of regimen is associated with greater toxicity than is non-anthracycline chemotherapy.^9^, ^12^ The incidence of non-breast second primary cancers did not appear to be reduced by trastuzumab, indicating that the reduced incidence of such cancers in the initial NSABP trial B-31 and the NCCTG trial N9831 report^4^ might have been a chance finding.

Most trials in this meta-analysis treated patients with trastuzumab for 12 months and the recurrence reductions were largest during this treatment period. Somewhat less benefit was found during years 2–4 and 5–9, after any trastuzumab treatment had been completed, raising the possibility that extended treatment with trastuzumab might have been more efficacious. Nevertheless, in the HERA trial, 2 years of trastuzumab did not appear to provide any greater benefit than 1 year of treatment.^21^ Switching to another HER2-directed therapy, neratinib, after 1 year of trastuzumab appeared more effective than continuing trastuzumab.^22^ We could not compare the efficacy of trastuzumab for 1 year versus less than 1 year as only the FinHER trial tested a shorter treatment time of 9 weeks. We included this small trial in these analyses, even though this treatment duration appears inadequate.^23^ However, the overall results would not be materially different if it were excluded. Three trials comparing 1 year versus 6 months of trastuzumab treatment, which were not included in this meta-analysis, all reported fewer recurrences with 12 months than with 6 months of trastuzumab, but had conflicting interpretations as to whether or not 12 months of treatment was more effective than 6 months.^24^, ^25^, ^26^ Despite these trials not being included in our main analysis, we did a post-hoc meta-analysis of their published results, which showed fewer recurrences with 12 months than with 6 months of trastuzumab (appendix p 30); however, an individual patient-level data meta-analysis of these trials is needed to help clarify the benefits and risks of extended trastuzumab treatment.

The efficacy of trastuzumab could be enhanced by adding other HER-directed therapies, such as lapatinib or pertuzumab,^27^, ^28^ and by giving combination HER2-directed therapy before surgery.^29^ Adapting treatment according to initial tumour response is another approach, as in the KATHERINE trial, in which switching to trastuzumab–emtansine (an antibody–drug conjugate) was more effective than continuing trastuzumab in patients with residual disease following neoadjuvant chemotherapy plus trastuzumab.^30^ Further understanding of how cancer biology affects outcome, such as the relevance of tumour-infiltrating lymphocytes or tumour genomics,^31^ might also help to individualise treatment approaches.

In summary, this meta-analysis confirms that breast cancer recurrence and mortality can be reduced by a third by adding 1 year of trastuzumab treatment to adjuvant chemotherapy in patients with early-stage, HER2-positive breast cancer. The proportional reduction was unaffected by any of the measured tumour or patient characteristics. Because optimal anthracycline and taxane-based chemotherapy schedules also reduce the risk of breast cancer mortality by about a third, regardless of HER2 status or other characteristics,^12^, ^17^ the combination of chemotherapy plus trastuzumab for HER2-positive breast cancer could reduce the risk of death from breast cancer by about 50%, compared with receiving neither chemotherapy nor trastuzumab.

Correspondence to: EBCTCG Secretariat, Clinical Trial Service Unit, Nuffield Department of Population Health, Oxford OX3 7LF, UK **bc.overview@ctsu.ox.ac.uk**

## Data sharing

The data sharing policy is available online at https://www.ndph.ox.ac.uk/data-access.
