# Supplementary material for: Trastuzumab for early-stage, HER2-positive breast cancer: a meta-analysis of 13 864 women in seven randomised trials
Source: Lancet Oncol. 2021 Aug;22(8):1139–50. doi: 10.1016/S1470-2045(21)00288-6 (PMC8324484; doi:10.1016/S1470-2045(21)00288-6)
Supplement: Supplementary appendix [file mmc1.pdf]

THE LANCET
Oncology

Supplementary appendix

This appendix formed part of the original submission and has been peer reviewed. We post it as supplied by the authors.

Supplement to: Early Breast Cancer Trialists' Collaborative group (EBCTCG). Trastuzumab for early-stage, HER2-positive breast cancer: a meta-analysis of 13 864 women in seven randomised trials. *Lancet Oncol* 2021; **22:** 1139–50.

Holding page for journal to replace

CONTENTS LIST (Click on any listed item with a page number to jump to it)

Pages 3-10: Forest plots, one line per-trial, for 8 different endpoints (any recurrence, distant recurrence at any time, local recurrence as first event, contralateral recurrence as first event, breast cancer mortality, death without recurrence in year 0 (comparing concurrent chemotherapy and trastuzumab versus sequential chemotherapy then trastuzumab), death without recurrence, all-cause mortality)

Page 11: Kaplan-Meier graphs, for 4 different endpoints (any recurrence, distant recurrence at any time, local recurrence as first event, contralateral recurrence as first event)

Page 12: Subgroup analyses for any recurrence time period by ER-negative and ER-positive

Page 13: 10-year breast cancer mortality and all-cause mortality by ER-negative and ER-positive

Page 14: Any recurrence, excluding contralateral, by time period for ER-positive tumours split by PR status

Page 15: Subgroups analyses for recurrence

Page 16: Subgroup analyses for distant recurrence

Page 17: Subgroup analyses for breast cancer mortality

Page 18: Sites of distant recurrence (isolated CNS recurrence)

Page 19: Mortality by cause and incidence of second cancers

Pages 20-24: Sensitivity analyses

Page 20: Any recurrence including TCH arm from BCIRG 006

Pages 21-22: Analyses accounting for crossover

Pages 23-24: Concurrent versus sequential trastuzumab and chemotherapy direct randomised comparisons in the NCCTG 9831 trial

Page 25: Death without recurrence by tumour size and nodal status

Pages 26-29: Toxicity (as reported in trastuzumab study reports)

Page 30: Published trial results of recurrence from trials comparing longer versus shorter trastuzumab duration

Pages 31-38: Statistical Analysis Plan including list of relevant trials and publications, and variables requested

P3: Recurrence in trials of trastuzumab versus no trastuzumab

Year started and study name	Treatment comparison	Events/Women: Allocated Trastuz.	Events/Women: Adjusted control	Trastuz. events: Logrank O−E	Trastuz. events: Variance of O−E
**(a) Trials of <1yr of trastuzumab vs no trastuzumab**					
2000 FinHer/FBCG 00−01	((3**D**$_{100}$**q3** v 9**Vin**$_{25}$**q1**) ± 9**Tr q1**); 3**F**$_{600}$**E**$_{60}$**C**$_{600}$	36/115	39/116	−2·3	14·0
**(a) subtotal**		**36/ 115 (31·3%)**	**39/ 116 (33·6%)**	**−2·3**	**14·0**
**(b) Trials of >=1yr of trastuzumab vs no trastuzumab**					
2000 NSABP B−31	4**AC q3**; (4**P**$_{175}$**q3** ± 12mths **Tr q1**)	221/1058	351/1061	−77·6	133·5
2000 NCCTG/Int.N9831	4**AC q3**; (12**P**$_{80}$ **q1**; 12mths **Tr q1** vs not)	33/162	60/166	−14·5	19·5
2000 NCCTG/Int.N9831 *	4**AC q3**; (12**P**$_{80}$ **q1** ± 12mths **Tr q1** (Seq. or Concur.))	454/2111	2(287/1066)	−51·7	151·2
2001 HERA/BIG 01−01 *	Chemo; 12 or 24mths **Tr q3** vs not	907/3402	2(563/1697)	−105·4	289·0
2001 PACS 04 France	6(**F**$_{500}$**E**$_{100}$**C**$_{500}$ v **E**$_{75}$**D**$_{75}$); 12mths **Tr q3** vs not	74/260	96/268	−8·7	38·4
2001 BCIRG 006	4**AC q3**; (4**D**$_{100}$**q3** ± 12mths **Tr q1**)	197/1074	261/1073	−46·2	106·5
2002 NOAH	neo(3**A**$_{60}$**P**$_{150}$**q3**; (4**P**$_{175}$**q3**;3**CMF q4**) ± 12mths **Tr q3**)	44/117	62/118	−9·6	19·5
**(b) subtotal**		**1930/ 8184 (23·6%)**	**2530/ 8212 (30·8%)**	**−313·8**	**757·5**
**Total (a + b)**		**1966/ 8299 (23·7%)**	**2569/ 8328 (30·8%)**	**−316·1**	**771·6**

Heterogeneity between 7 trials: $\chi^2_6 = 8{\cdot}9$; p > 0·1; NS

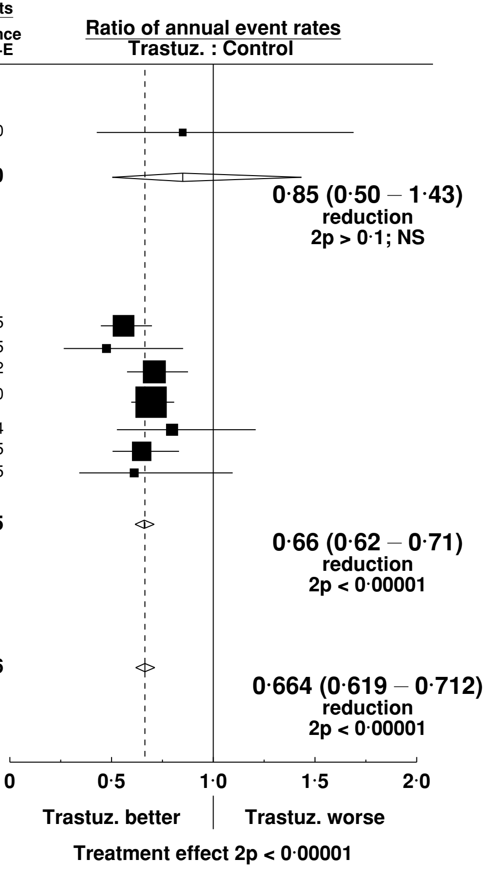

99% or 95% confidence intervals

Difference between treatment effects in 2 subtotals: $\chi^2_1 = 0{\cdot}9$; 2p > 0·1; NS

Heterogeneity within subtotals: $\chi^2_6 = 8{\cdot}9$; p > 0·1; NS

Heterogeneity between 8 trials: $\chi^2_7 = 9{\cdot}7$; p > 0·1; NS

* For balance, control patients in 3−way trials or trial strata count half or twice in subtotal(s) and in final total of events/patients.

P4: Distant recurrence at any time in trials of trastuzumab versus no trastuzumab

Year started and study name	Treatment comparison	Events/Women: Allocated Trastuz.	Events/Women: Adjusted control	Trastuz. events: Logrank O–E	Trastuz. events: Variance of O–E	Ratio of annual event rates Trastuz. : Control
**(a) Trials of <1yr of trastuzumab vs no trastuzumab**						
2000 FinHer/FBCG 00–01	((3$\mathbf{D}_{100}$**q3** v 9$\mathbf{Vin}_{25}$**q1**) ± 9**Tr q1**); 3$\mathbf{F}_{600}\mathbf{E}_{60}\mathbf{C}_{600}$	28/115	36/116	−5·0	11·6	
**(a) subtotal**		**28/ 115 (24·3%)**	**36/ 116 (31·0%)**	**−5·0**	**11·6**	**0·65 (0·36 – 1·15) reduction 2p > 0·1; NS**
**(b) Trials of >=1yr of trastuzumab vs no trastuzumab**						
2000 NSABP B–31	4**AC q3**; (4$\mathbf{P}_{175}$**q3** ± 12mths **Tr q1**)	169/1058	284/1061	−64·5	106·3	
2000 NCCTG/Int.N9831	4**AC q3**; (12$\mathbf{P}_{80}$ **q1**; 12mths **Tr q1** vs not)	28/162	55/166	−13·8	17·9	
2000 NCCTG/Int.N9831 *	4**AC q3**; (12$\mathbf{P}_{80}$ **q1** ± 12mths **Tr q1** (Seq. or Concur.))	341/2111	2(232/1066)	−49·4	117·8	
2001 HERA/BIG 01–01 *	Chemo; 12 or 24mths **Tr q3** vs not	710/3402	2(470/1697)	−101·6	234·3	
2001 PACS 04 France	6($\mathbf{F}_{500}\mathbf{E}_{100}\mathbf{C}_{500}$ v $\mathbf{E}_{75}\mathbf{D}_{75}$); 12mths **Tr q3** vs not	61/260	84/268	−9·0	33·1	
2001 BCIRG 006	4**AC q3**; (4$\mathbf{D}_{100}$**q3** ± 12mths **Tr q1**)	164/1074	229/1073	−43·6	92·0	
2002 NOAH	neo(3$\mathbf{A}_{60}\mathbf{P}_{150}$**q3**; (4$\mathbf{P}_{175}$**q3**;3**CMF q4**) ± 12mths **Tr q3**)	39/117	54/118	−7·9	17·5	
**(b) subtotal**		**1512/ 8184 (18·5%)**	**2110/ 8212 (25·7%)**	**−289·8**	**618·8**	**0·63 (0·58 – 0·68) reduction 2p < 0·00001**
**Total (a + b)**		**1540/ 8299 (18·6%)**	**2146/ 8328 (25·8%)**	**−294·9**	**630·4**	**0·626 (0·579 – 0·677) reduction 2p < 0·00001**

Heterogeneity between 7 trials: $\chi^2_6 = 5·5$; p > 0·1; NS

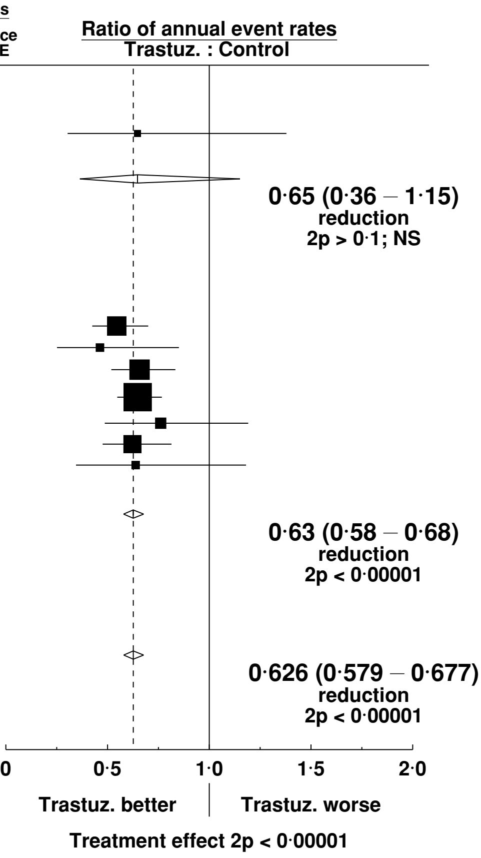

99% or 95% confidence intervals

Difference between treatment effects in 2 subtotals: $\chi^2_1 = 0·0$; 2p > 0·1; NS

Heterogeneity within subtotals: $\chi^2_6 = 5·5$; p > 0·1; NS

Heterogeneity between 8 trials: $\chi^2_7 = 5·5$; p > 0·1; NS

* For balance, control patients in 3–way trials or trial strata count half or twice in subtotal(s) and in final total of events/patients.

P5: Isolated local recurrence as first event in trials of trastuzumab versus no trastuzumab

Year started and study name	Treatment comparison	Events/Women: Allocated Trastuz.	Events/Women: Adjusted control	Trastuz. events: Logrank O–E	Trastuz. events: Variance of O–E	Ratio of annual event rates Trastuz. : Control
**(a) Trials of <1yr of trastuzumab vs no trastuzumab**						
2000 FinHer/FBCG 00–01	((3$\mathbf{D}_{100}$**q3** v 9**Vin**$_{25}$**q1**) ± 9**Tr q1**); 3$\mathbf{F}_{600}\mathbf{E}_{60}\mathbf{C}_{600}$	7/115	7/116	0·0	2·9	
**(a) subtotal**		**7/ 115 (6·1%)**	**7/ 116 (6·0%)**	**0·0**	**2·9**	**1·00 (0·32 – 3·16) increase 2p > 0·1; NS**
**(b) Trials of >=1yr of trastuzumab vs no trastuzumab**						
2000 NSABP B–31	4**AC q3**; (4$\mathbf{P}_{175}$**q3** ± 12mths **Tr q1**)	47/1058	70/1061	−14·3	28·7	
2000 NCCTG/Int.N9831	4**AC q3**; (12$\mathbf{P}_{80}$ **q1**; 12mths **Tr q1** vs not)	3/162	5/166	−1·3	1·9	
2000 NCCTG/Int.N9831 *	4**AC q3**; (12$\mathbf{P}_{80}$ **q1** ± 12mths **Tr q1** (Seq. or Concur.))	73/2111	2(37/1066)	−2·3	23·4	
2001 HERA/BIG 01–01 *	Chemo; 12 or 24mths **Tr q3** vs not	196/3402	2(126/1697)	−25·3	67·0	
2001 PACS 04 France	6($\mathbf{F}_{500}\mathbf{E}_{100}\mathbf{C}_{500}$ v $\mathbf{E}_{75}\mathbf{D}_{75}$); 12mths **Tr q3** vs not	10/260	21/268	−5·4	7·6	
2001 BCIRG 006	4**AC q3**; (4$\mathbf{D}_{100}$**q3** ± 12mths **Tr q1**)	41/1074	36/1073	0·3	18·9	
2002 NOAH	neo(3$\mathbf{A}_{60}\mathbf{P}_{150}$**q3**; (4$\mathbf{P}_{175}$**q3**;3**CMF q4**) ± 12mths **Tr q3**)	8/117	13/118	−2·2	4·6	
**(b) subtotal**		**378/ 8184 (4·6%)**	**471/ 8212 (5·7%)**	**−50·5**	**152·2**	**0·72 (0·61 – 0·84) reduction 2p = 0·00004**
**Total (a + b)**		**385/ 8299 (4·6%)**	**478/ 8328 (5·7%)**	**−50·5**	**155·1**	**0·722 (0·617 – 0·845) reduction 2p = 0·00005**

Heterogeneity between 7 trials: χ^2_6 = 5·8; p > 0·1; NS

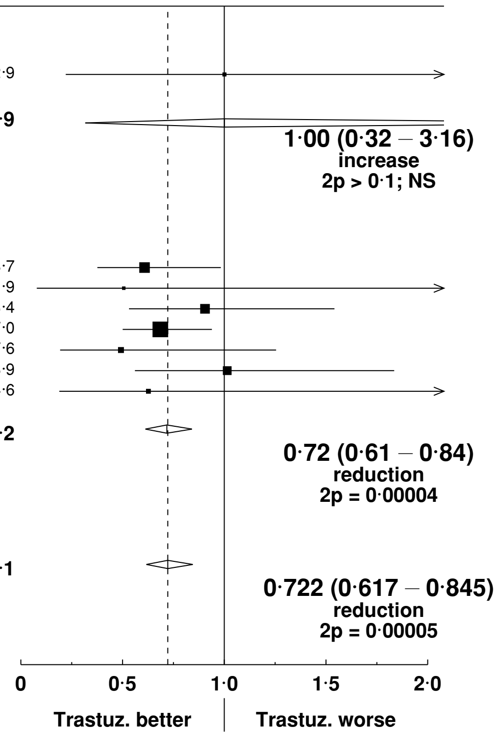

99% or 95% confidence intervals

Difference between treatment effects in 2 subtotals: χ^2_1 = 0·3; 2p > 0·1; NS

Heterogeneity within subtotals: χ^2_6 = 5·8; p > 0·1; NS

Heterogeneity between 8 trials: χ^2_7 = 6·2; p > 0·1; NS

* For balance, control patients in 3–way trials or trial strata count half or twice in subtotal(s) and in final total of events/patients.

P6: Isolated contralateral recurrence as first event in trials of trastuzumab versus no trastuzumab

Year started and study name	Treatment comparison	Events/Women: Allocated Trastuz.	Events/Women: Adjusted control	Trastuz. events: Logrank O–E	Trastuz. events: Variance of O–E
**(a) Trials of <1yr of trastuzumab vs no trastuzumab**					
2000 FinHer/FBCG 00–01	((3D$_{100}$q3 v 9Vin$_{25}$q1) ± 9Tr q1); 3F$_{600}$E$_{60}$C$_{600}$	4/115	1/116	1·2	1·1
. **(a) subtotal**		**4/ 115 (3·5%)**	**1/ 116 (0·9%)**	**1·2**	**1·1**
**(b) Trials of >=1yr of trastuzumab vs no trastuzumab**					
2000 NSABP B–31	4AC q3; (4P$_{175}$q3 ± 12mths Tr q1)	28/1058	34/1061	–5·7	15·2
2000 NCCTG/Int.N9831	4AC q3; (12P$_{80}$ q1; 12mths Tr q1 vs not)	3/162	2/166	–0·0	0·9
2000 NCCTG/Int.N9831 *	4AC q3; (12P$_{80}$ q1 ± 12mths Tr q1 (Seq. or Concur.))	65/2111	2(27/1066)	2·3	19·2
2001 HERA/BIG 01–01 *	Chemo; 12 or 24mths Tr q3 vs not	89/3402	2(41/1697)	–0·7	27·3
2001 PACS 04 France	6(F$_{500}$E$_{100}$C$_{500}$ v E$_{75}$D$_{75}$); 12mths Tr q3 vs not	8/260	5/268	1·3	3·1
2001 BCIRG 006	4AC q3; (4D$_{100}$q3 ± 12mths Tr q1)	4/1074	10/1073	–3·6	3·4
2002 NOAH	neo(3A$_{60}$P$_{150}$q3; (4P$_{175}$q3;3CMF q4) ± 12mths Tr q3)	2/117	3/118	–0·3	0·9
■ **(b) subtotal**		**199/ 8184 (2·4%)**	**190/ 8212 (2·3%)**	**–6·7**	**70·0**
■ **Total (a + b)**		**203/ 8299 (2·4%)**	**191/ 8328 (2·3%)**	**–5·5**	**71·2**

Heterogeneity between 7 trials: χ^2_6 = 6·2; p > 0·1; NS

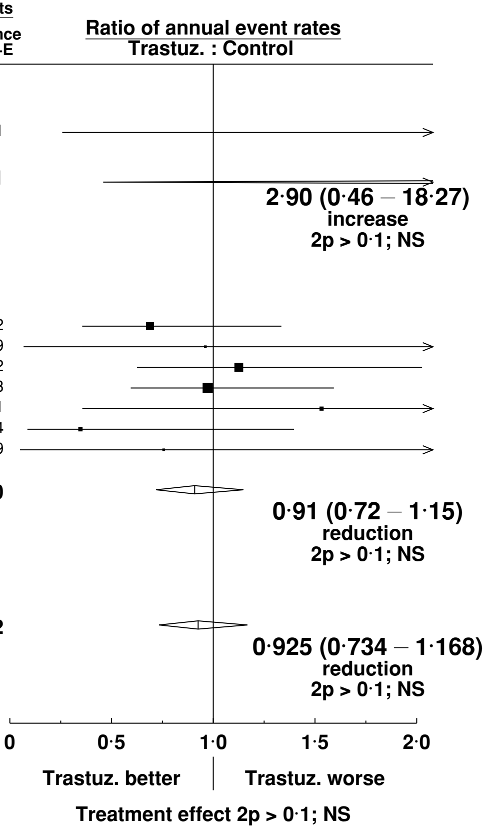

■ 99% or ◇ 95% confidence intervals

Difference between treatment effects in 2 subtotals: χ^2_1 = 1·5; 2p > 0·1; NS

Heterogeneity within subtotals: χ^2_6 = 6·2; p > 0·1; NS

Heterogeneity between 8 trials: χ^2_7 = 7·7; p > 0·1; NS

* For balance, control patients in 3–way trials or trial strata count half or twice in subtotal(s) and in final total of events/patients.

P7: Breast cancer mortality in trials of trastuzumab versus no trastuzumab

Year started and study name	Treatment comparison	Deaths/Women: Allocated Trastuz.	Deaths/Women: Adjusted control	Trastuz. deaths: Logrank O−E	Trastuz. deaths: Variance of O−E
**(a) Trials of <1yr of trastuzumab vs no trastuzumab**					
2000 FinHer/FBCG 00−01	((3D_{100}**q3** v 9Vin_{25}**q1**) ± 9**Tr q1**); 3$F_{600}E_{60}C_{600}$	22/115	26/116	−3·6	8·5
**(a) subtotal**		**22/ 115 (19·1%)**	**26/ 116 (22·4%)**	**−3·6**	**8·5**
**(b) Trials of >=1yr of trastuzumab vs no trastuzumab**					
2000 NSABP B−31	4**AC q3**; (4P_{175}**q3** ± 12mths **Tr q1**)	146/1058	218/1061	−40·3	86·7
2000 NCCTG/Int.N9831	4**AC q3**; (12P_{80} **q1**; 12mths **Tr q1** vs not)	25/162	40/166	−6·8	14·0
2000 NCCTG/Int.N9831 *	4**AC q3**; (12P_{80} **q1** ± 12mths **Tr q1** (Seq. or Concur.))	292/2111	2(188/1066)	−32·5	100·6
2001 HERA/BIG 01−01 *	Chemo; 12 or 24mths **Tr q3** vs not	580/3402	2(383/1697)	−77·4	197·7
2001 PACS 04 France	6($F_{500}E_{100}C_{500}$ v $E_{75}D_{75}$); 12mths **Tr q3** vs not	44/260	54/268	−2·1	23·1
2001 BCIRG 006	4**AC q3**; (4D_{100}**q3** ± 12mths **Tr q1**)	121/1074	181/1073	−37·0	71·8
2002 NOAH	neo(3$A_{60}P_{150}$**q3**; (4P_{175}**q3**;3**CMF q4**) ± 12mths **Tr q3**)	26/117	45/118	−9·1	14·7
**(b) subtotal**		**1234/ 8184 (15·1%)**	**1680/ 8212 (20·5%)**	**−205·2**	**508·7**
**Total (a + b)**		**1256/ 8299 (15·1%)**	**1706/ 8328 (20·5%)**	**−208·7**	**517·2**

Heterogeneity between 7 trials: $\chi^2_6 = 5·0$; p > 0·1; NS

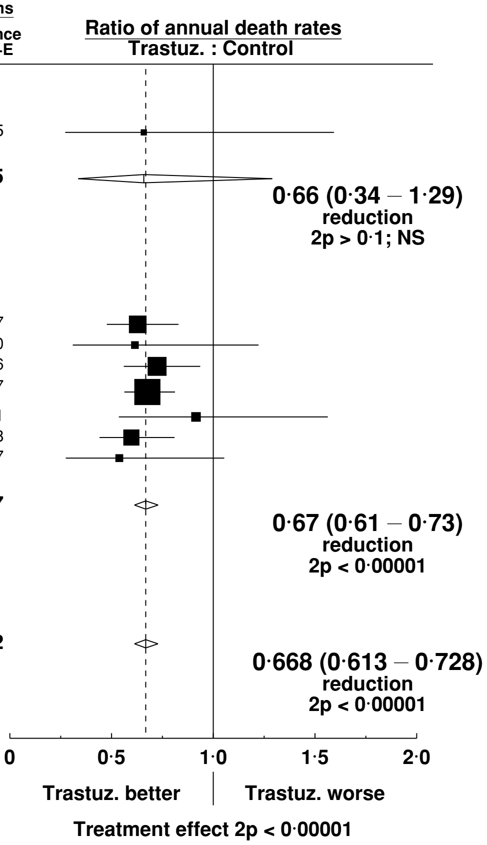

■ 99% or ◇ 95% confidence intervals

Difference between treatment effects in 2 subtotals: $\chi^2_1 = 0·0$; 2p > 0·1; NS

Heterogeneity within subtotals: $\chi^2_6 = 5·0$; p > 0·1; NS

Heterogeneity between 8 trials: $\chi^2_7 = 5·0$; p > 0·1; NS

* For balance, control patients in 3−way trials or trial strata count half or twice in subtotal(s) and in final total of deaths/patients.

P8: Death without recurrence in year 0, comparing concurrent chemotherapy and trastuzumab with sequential chemotherapy then trastuzumab

Category	Events/Women: Allocated Trastuz.	Events/Women: Allocated control	Trastuz. events: Logrank O−E	Trastuz. events: Variance of O−E
**Death without recurrence** (χ_1^2 = 1.1; 2p > 0.1; NS)				
**Concurrent**	11/3421 (0·3%)	7/3434 (0·2%)	2·2	4·4
**Sequential**	20/4878 (0·4%)	6/4894 (0·1%)	6·5	5·6
**Total**	**31/ 8299 (0·4%)**	**13/ 8328 (0·2%)**	**6·8**	**8·9**

■ 99% or ◇ 95% confidence intervals

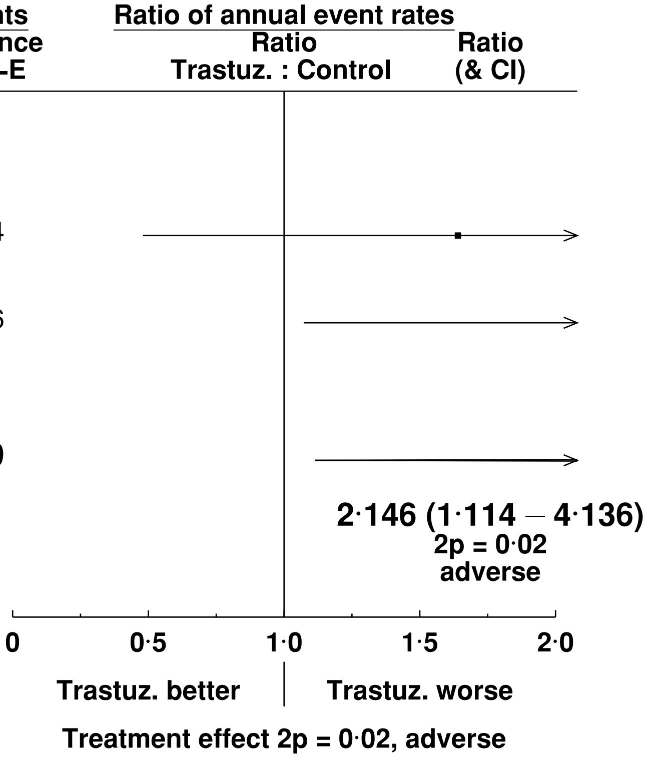

P9: Death without recurrence in trials of trastuzumab versus no trastuzumab

Year started and study name	Treatment comparison	Deaths/Women: Allocated Trastuz.	Deaths/Women: Adjusted control	Trastuz. deaths: Logrank O–E	Trastuz. deaths: Variance of O–E
**(a) Trials of <1yr of trastuzumab vs no trastuzumab**					
2000 FinHer/FBCG 00–01	((3**D**$_{100}$**q3** v 9**Vin**$_{25}$**q1**) ± 9**Tr q1**); 3**F**$_{600}$**E**$_{60}$**C**$_{600}$	1/115	0/116	0·2	0·2
**(a) subtotal**		**1/ 115 (0·9%)**	**0/ 116 (0·0%)**	**0·2**	**0·2**
**(b) Trials of >=1yr of trastuzumab vs no trastuzumab**					
2000 NSABP B–31	4**AC q3**; (4**P**$_{175}$**q3** ± 12mths **Tr q1**)	26/1058	34/1061	–7·1	14·4
2000 NCCTG/Int.N9831	4**AC q3**; (12**P**$_{80}$ **q1**; 12mths **Tr q1** vs not)	9/162	6/166	1·4	3·2
2000 NCCTG/Int.N9831 *	4**AC q3**; (12**P**$_{80}$ **q1** ± 12mths **Tr q1** (Seq. or Concur.))	100/2111	2(45/1066)	–1·0	29·8
2001 HERA/BIG 01–01 *	Chemo; 12 or 24mths **Tr q3** vs not	52/3402	2(22/1697)	0·9	15·3
2001 PACS 04 France	6(**F**$_{500}$**E**$_{100}$**C**$_{500}$ v **E**$_{75}$**D**$_{75}$); 12mths **Tr q3** vs not	3/260	5/268	–1·0	1·9
2001 BCIRG 006	4**AC q3**; (4**D**$_{100}$**q3** ± 12mths **Tr q1**)	20/1074	22/1073	–2·6	10·1
2002 NOAH	neo(3**A**$_{60}$**P**$_{150}$**q3**; (4**P**$_{175}$**q3**;3**CMF q4**) ± 12mths **Tr q3**)	2/117	0/118	0·9	0·5
**(b) subtotal**		**212/ 8184 (2·6%)**	**201/ 8212 (2·4%)**	**–8·4**	**75·3**
**Total (a + b)**		**213/ 8299 (2·6%)**	**201/ 8328 (2·4%)**	**–8·2**	**75·5**

Heterogeneity between 7 trials: $\chi^2_6 = 6{\cdot}1$**; p > 0·1; NS**

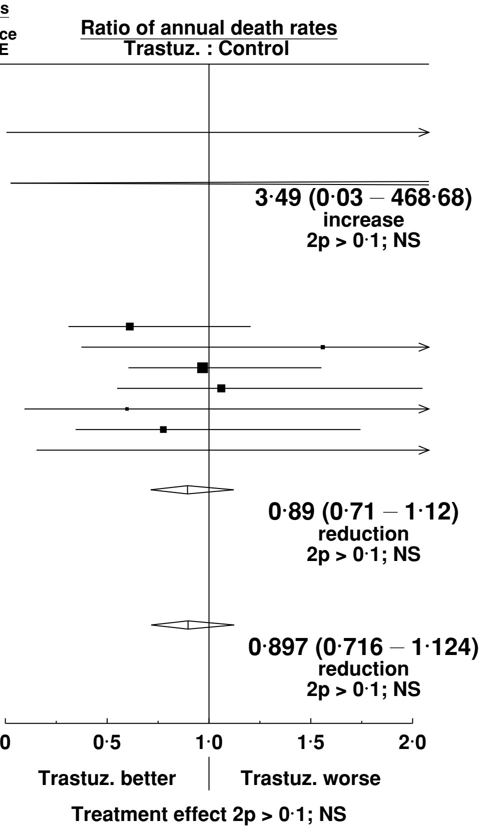

99% or 95% confidence intervals

Difference between treatment effects in 2 subtotals: $\chi^2_1 = 0{\cdot}3$**; 2p > 0·1; NS**

Heterogeneity within subtotals: $\chi^2_6 = 6{\cdot}1$**; p > 0·1; NS**

Heterogeneity between 8 trials: $\chi^2_7 = 6{\cdot}4$**; p > 0·1; NS**

* For balance, control patients in 3–way trials or trial strata count half or twice in subtotal(s) and in final total of deaths/patients.

P10: All-cause mortality in trials of trastuzumab versus no trastuzumab

Year started and study name	Treatment comparison	Deaths/Women: Allocated Trastuz.	Deaths/Women: Adjusted control	Trastuz. deaths: Logrank O–E	Trastuz. deaths: Variance of O–E
**(a) Trials of <1yr of trastuzumab vs no trastuzumab**					
2000 FinHer/FBCG 00–01	((3D_{100}**q3** v 9Vin_{25}**q1**) ± 9**Tr q1**); 3$F_{600}E_{60}C_{600}$	23/115	26/116	−3·4	8·7
**(a) subtotal**		**23/ 115 (20·0%)**	**26/ 116 (22·4%)**	**−3·4**	**8·7**
**(b) Trials of >=1yr of trastuzumab vs no trastuzumab**					
2000 NSABP B–31	4**AC q3**; (4P_{175}**q3** ± 12mths **Tr q1**)	172/1058	252/1061	−47·4	101·1
2000 NCCTG/Int.N9831	4**AC q3**; (12P_{80} **q1**; 12mths **Tr q1** vs not)	34/162	46/166	−5·4	17·3
2000 NCCTG/Int.N9831 *	4**AC q3**; (12P_{80} **q1** ± 12mths **Tr q1** (Seq. or Concur.))	392/2111	2(233/1066)	−33·5	130·5
2001 HERA/BIG 01–01 *	Chemo; 12 or 24mths **Tr q3** vs not	632/3402	2(405/1697)	−76·5	213·0
2001 PACS 04 France	6($F_{500}E_{100}C_{500}$ v $E_{75}D_{75}$); 12mths **Tr q3** vs not	47/260	59/268	−3·1	25·1
2001 BCIRG 006	4**AC q3**; (4D_{100}**q3** ± 12mths **Tr q1**)	141/1074	203/1073	−39·6	81·9
2002 NOAH	neo(3$A_{60}P_{150}$**q3**; (4P_{175}**q3**;3**CMF q4**) ± 12mths **Tr q3**)	28/117	45/118	−8·2	15·1
**(b) subtotal**		**1446/ 8184 (17·7%)**	**1881/ 8212 (22·9%)**	**−213·6**	**584·0**
**Total (a + b)**		**1469/ 8299 (17·7%)**	**1907/ 8328 (22·9%)**	**−216·9**	**592·6**

Heterogeneity between 7 trials: $\chi^2_6 = 5{\cdot}8$; p > 0·1; NS

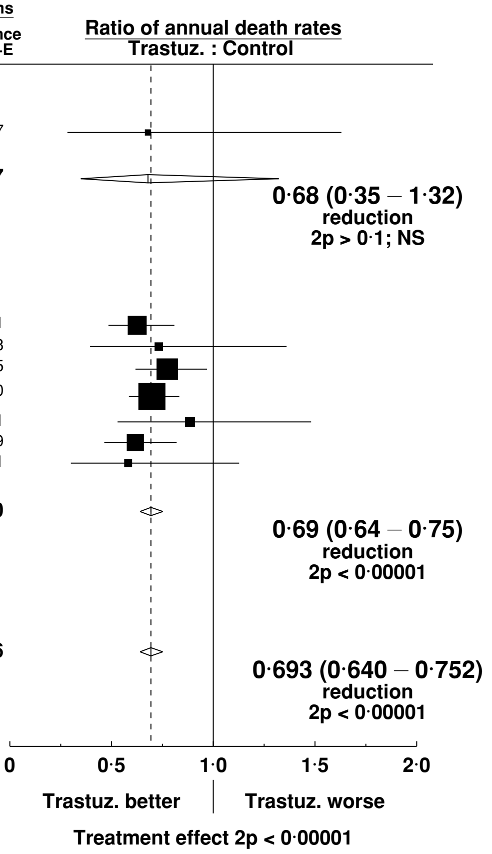

99% or 95% confidence intervals

Difference between treatment effects in 2 subtotals: $\chi^2_1 = 0{\cdot}0$; 2p > 0·1; NS

Heterogeneity within subtotals: $\chi^2_6 = 5{\cdot}8$; p > 0·1; NS

Heterogeneity between 8 trials: $\chi^2_7 = 5{\cdot}8$; p > 0·1; NS

* For balance, control patients in 3–way trials or trial strata count half or twice in subtotal(s) and in final total of deaths/patients.

P11: 10-year risk of (A) recurrence, (B) distant recurrence at any time, (C) isolated local recurrence as first event and (D) contralateral recurrence as first event in trials of trastuzumab versus no trastuzumab

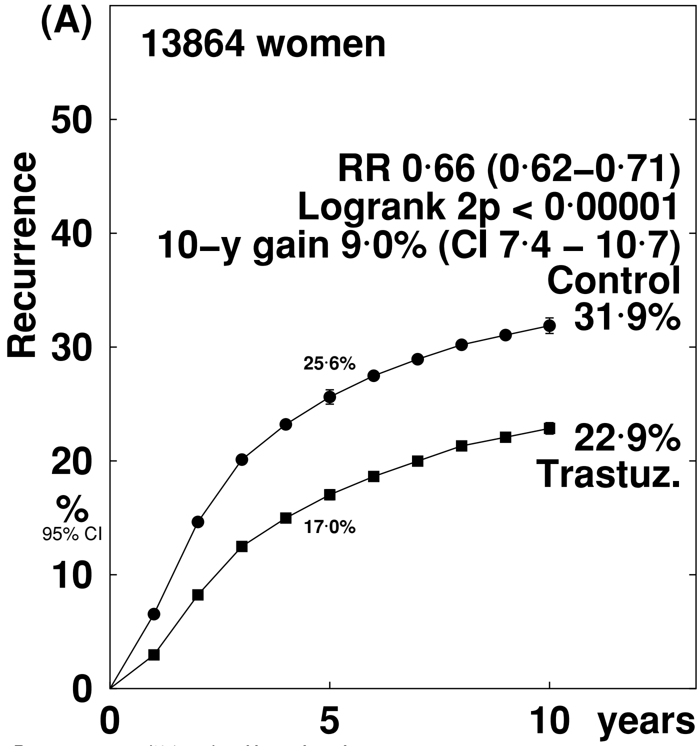

Recurrence rates (% / year) and logrank analyses

Allocation	Years 0 – 4	Years 5 – 9	Year 10+
Trastuz.	4·10 (1469 / 35853)	1·53 (422 / 27648)	0·95 (75 / 7858)
Control	6·24 (1385 / 22180)	1·87 (291 / 15601)	1·12 (43 / 3835)
Rate ratio, from	0·62 CI 0·58 – 0·66	0·83 CI 0·72 – 0·95	0·91 CI 0·63 – 1·31
(O–E) / V	–284·0 / 591·3	–29·6 / 154·9	–2·5 / 25·3

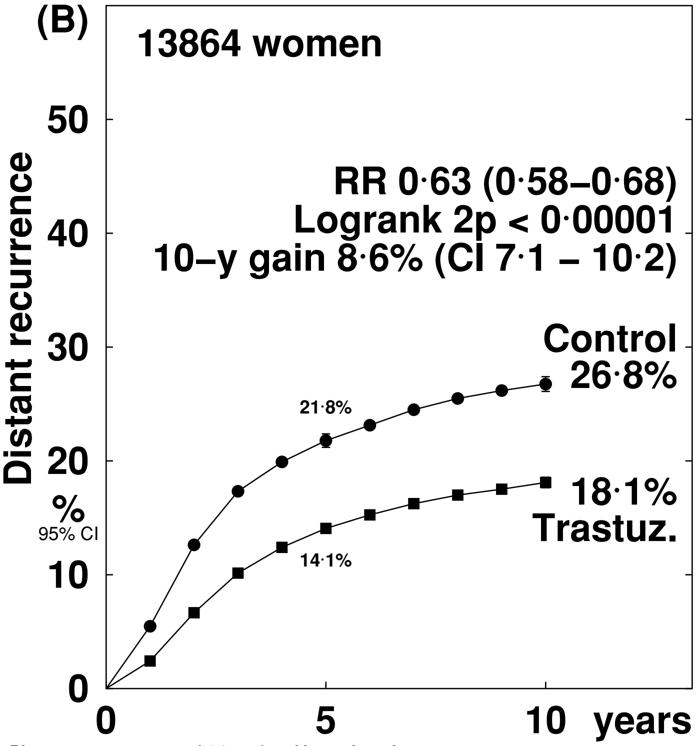

Distant recurrence rates (% / year) and logrank analyses

Allocation	Years 0 – 4	Years 5 – 9	Year 10+
Trastuz.	3·33 (1215 / 36522)	1·02 (297 / 29025)	0·33 (28 / 8376)
Control	5·19 (1182 / 22754)	1·42 (235 / 16579)	0·65 (27 / 4124)
Rate ratio, from	0·61 CI 0·57 – 0·65	0·73 CI 0·63 – 0·85	0·52 CI 0·34 – 0·79
(O–E) / V	–250·7 / 502·3	–36·4 / 116·3	–7·8 / 11·8

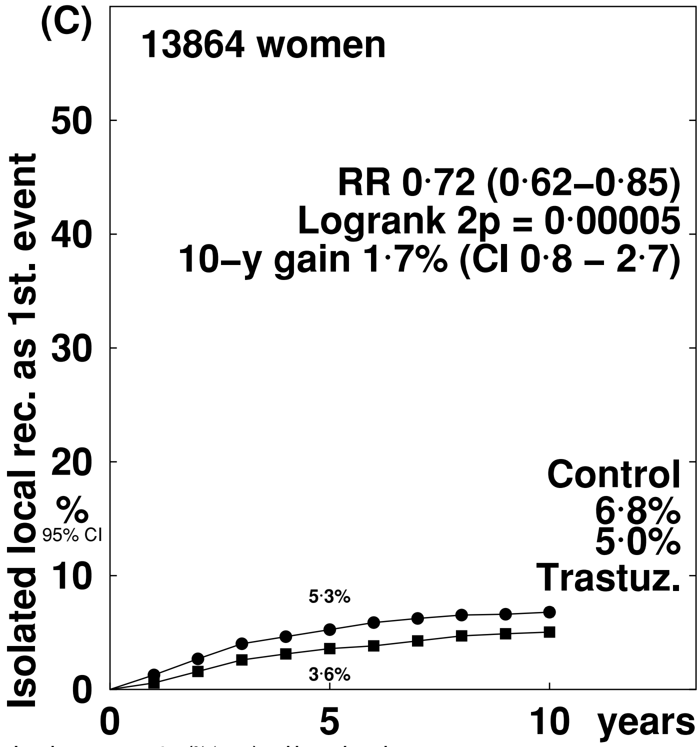

Local recurrence rates (% / year) and logrank analyses

Allocation	Years 0 – 4	Years 5 – 9	Year 10+
Trastuz.	0·79 (284 / 35853)	0·31 (85 / 27648)	0·20 (16 / 7858)
Control	1·13 (251 / 22180)	0·36 (56 / 15601)	0·21 (8 / 3835)
Rate ratio, from	0·67 CI 0·58 – 0·77	0·90 CI 0·65 – 1·25	1·13 CI 0·46 – 2·78
(O–E) / V	–47·7 / 118·0	–3·4 / 31·7	0·6 / 5·3

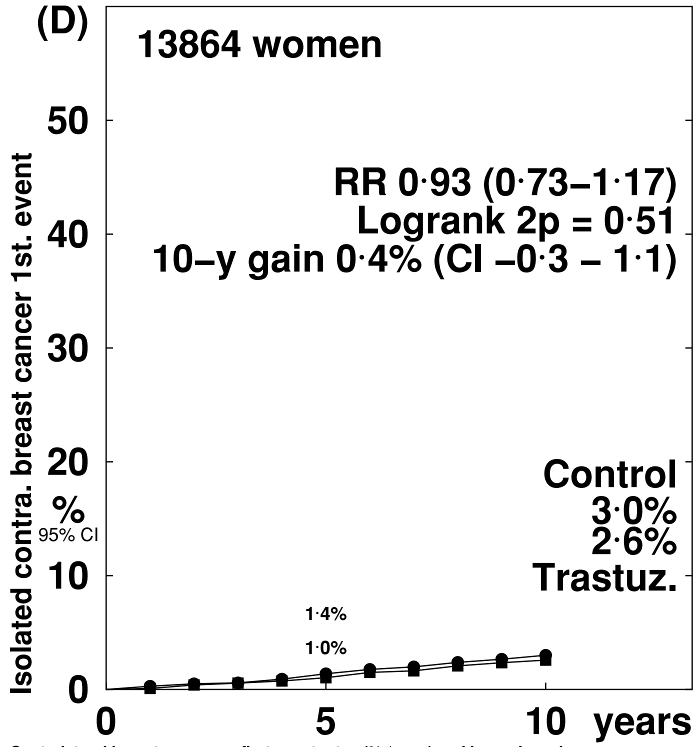

Contralateral breast cancer as first event rates (% / year) and logrank analyses

Allocation	Years 0 – 4	Years 5 – 9	Year 10+
Trastuz.	0·21 (76 / 35853)	0·33 (91 / 27648)	0·46 (36 / 7858)
Control	0·28 (62 / 22180)	0·32 (50 / 15601)	0·29 (11 / 3835)
Rate ratio, from	0·75 CI 0·55 – 1·02	0·96 CI 0·68 – 1·36	1·55 CI 0·72 – 3·37
(O–E) / V	–8·7 / 30·4	–1·2 / 30·7	4·5 / 10·1

P12: Any recurrence, excluding contralateral, by time period by ER-negative and ER-positive

Category	Events/woman-years: Allocated Trastuz.	Events/woman-years: Allocated control	Trastuz. events: Logrank O−E	Trastuz. events: Variance of O−E	Ratio (& CI)
**(a) ER− by time period (trend χ^2_1 =13.2; 2p = 0.0003)**					
**Year 0–1**	468/7569 (6·2%/y)	783/7087 (11·0%/y)	−133·1	200·0	**0·51 (0·43 – 0·62)**
**2–4**	334/9487 (3·5%/y)	355/8267 (4·3%/y)	−31·3	123·0	**0·78 (0·61 – 0·98)**
**5–9**	104/13068 (0·8%/y)	111/10955 (1·0%/y)	−9·2	37·8	**0·78 (0·52 – 1·19)**
**10+**	13/3608 (0·4%/y)	10/2903 (0·3%/y)	−0·3	4·3	
**(a) subtotal**	**919/ 33732 (2·7%/y)**	**1259/ 29212 (4·3%/y)**	**−173·8**	**365·1**	**0·62 (0·56 – 0·69) 2p < 0·00001**
**(b) ER+ by time period (trend χ^2_1 = 4.0; 2p = 0.05)**					
**Years 0–1**	251/8003 (3·1%/y)	409/7788 (5·3%/y)	−61·7	107·7	**0·56 (0·44 – 0·72)**
**2–4**	344/10577 (3·3%/y)	433/9656 (4·5%/y)	−51·3	135·3	**0·68 (0·55 – 0·85)**
**5–9**	228/14496 (1·6%/y)	248/12798 (1·9%/y)	−18·9	87·4	**0·81 (0·61 – 1·06)**
**10+**	27/4148 (0·7%/y)	38/3574 (1·1%/y)	−6·4	11·2	**0·57 (0·26 – 1·22)**
**(b) subtotal**	**850/ 37224 (2·3%/y)**	**1128/ 33816 (3·3%/y)**	**−138·3**	**341·5**	**0·67 (0·60 – 0·74) 2p < 0·00001**

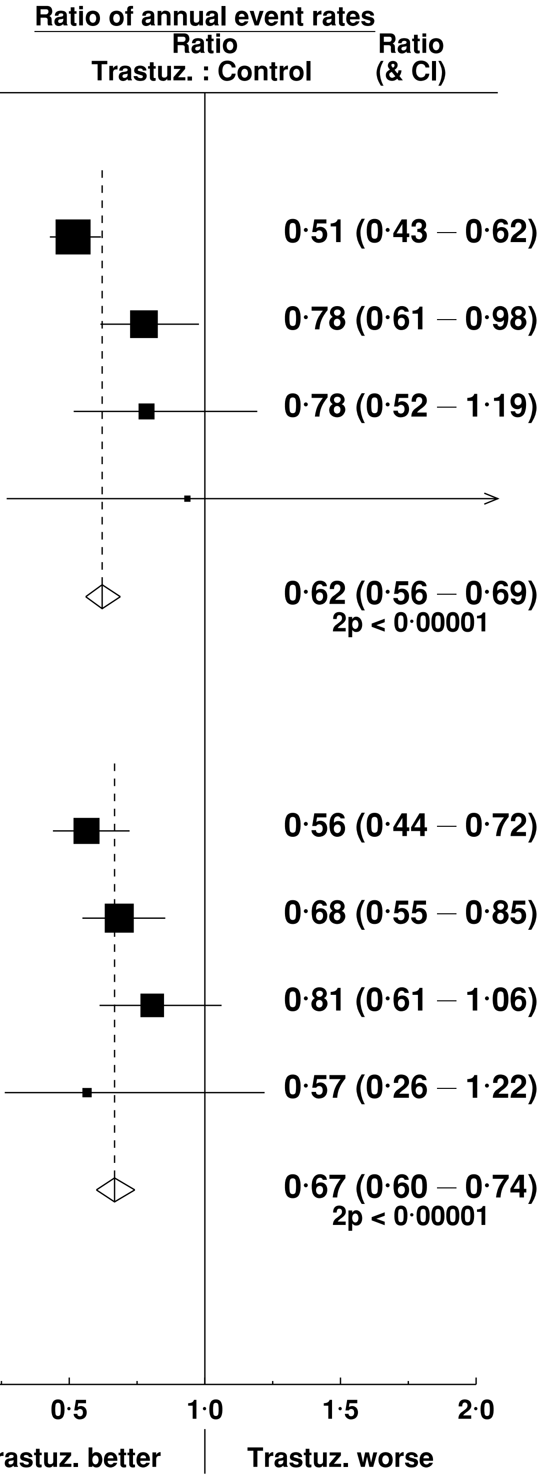

99% or 95% confidence intervals

P13: 10-year risk of (A) breast cancer mortality and (B) all-cause mortality by ER-negative and ER-positive

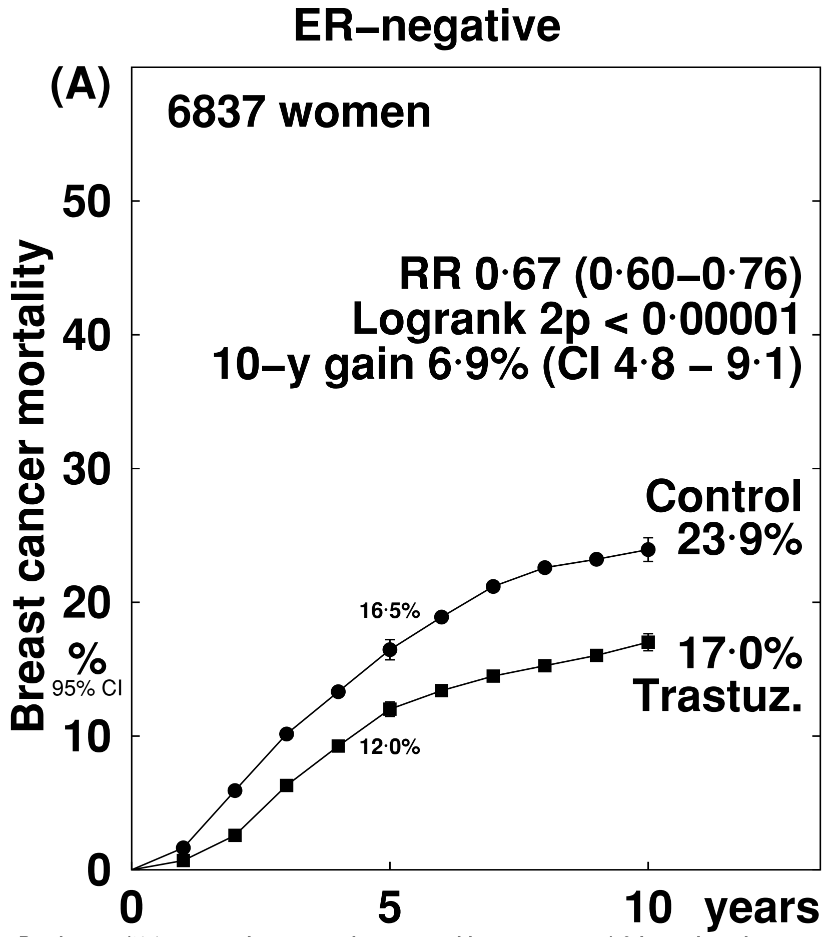

Death rates (% / year: total rate – rate in women without recurrence) & logrank analyses

Allocation	Years 0 – 4	Years 5 – 9	Year 10+
Trastuz.	2·62 CI 2·39 – 2·86	1·27 CI 1·09 – 1·45	0·47 CI 0·27 – 0·68
Control	3·67 CI 3·32 – 4·01	2·06 CI 1·76 – 2·36	0·67 CI 0·32 – 1·02
Rate ratio, from	0·70 CI 0·62 – 0·78	0·61 CI 0·51 – 0·72	0·76 CI 0·40 – 1·42
(O–E) / V	–74·0 / 206·7	–40·6 / 82·1	–2·1 / 7·4

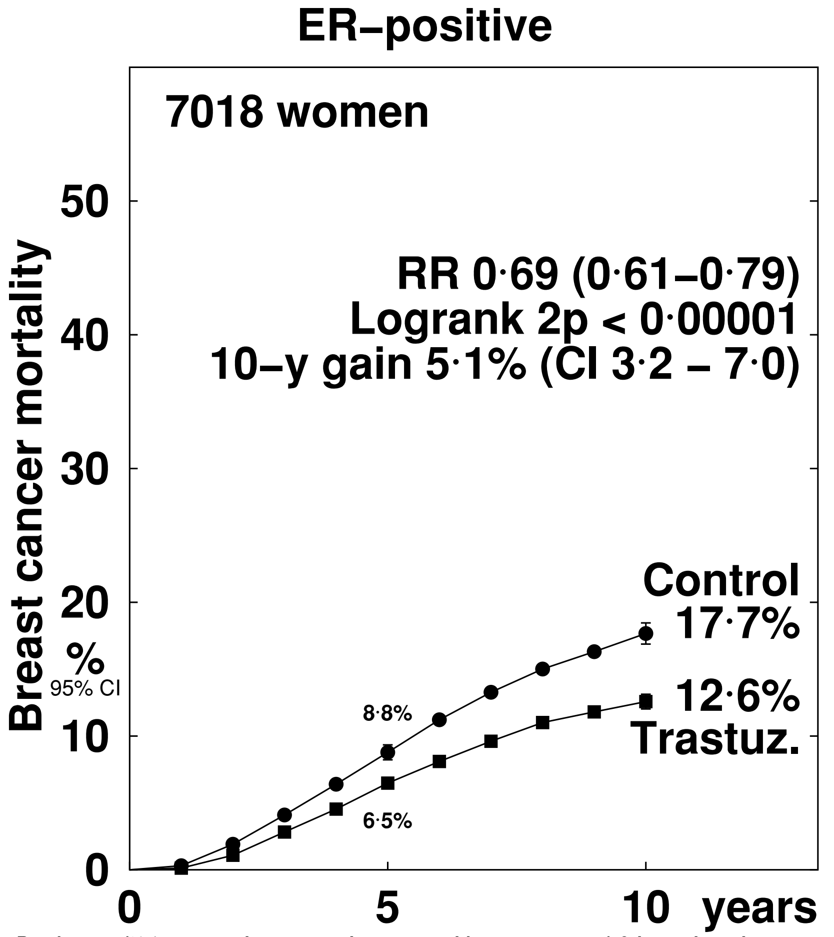

Death rates (% / year: total rate – rate in women without recurrence) & logrank analyses

Allocation	Years 0 – 4	Years 5 – 9	Year 10+
Trastuz.	1·37 CI 1·21 – 1·54	1·43 CI 1·25 – 1·62	0·91 CI 0·65 – 1·17
Control	1·85 CI 1·61 – 2·08	2·21 CI 1·92 – 2·50	1·51 CI 1·02 – 1·99
Rate ratio, from	0·73 CI 0·62 – 0·85	0·66 CI 0·57 – 0·78	0·67 CI 0·46 – 0·98
(O–E) / V	–37·0 / 115·4	–42·6 / 103·4	–7·3 / 18·3

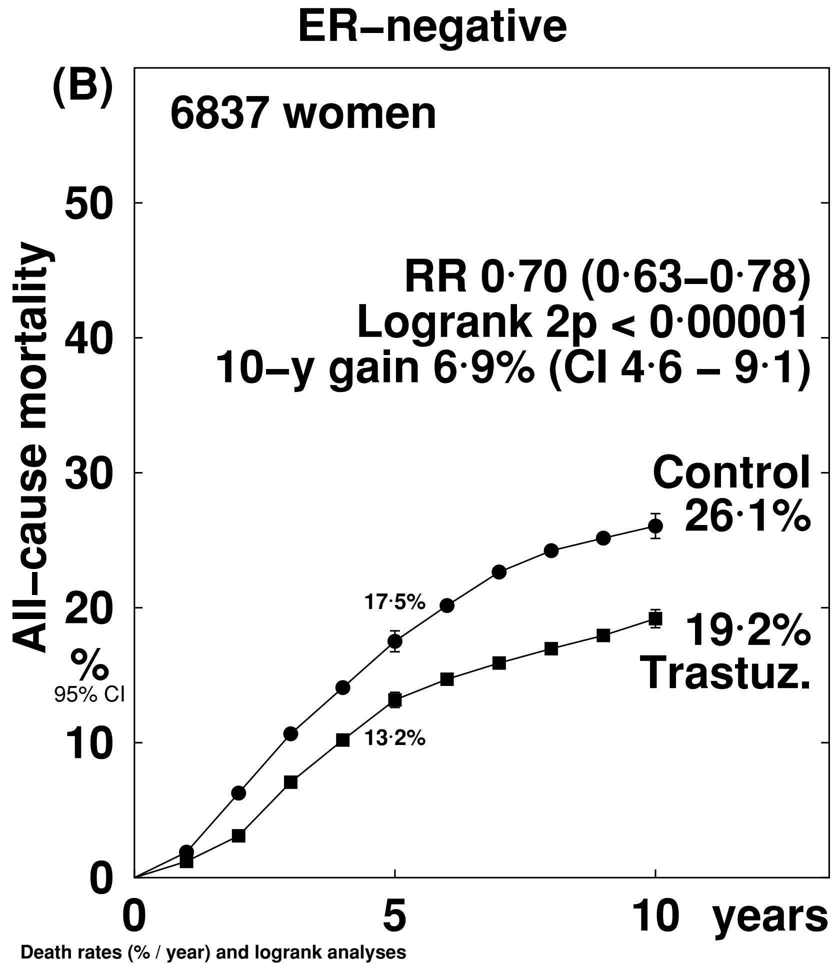

Death rates (% / year) and logrank analyses

Allocation	Years 0 – 4	Years 5 – 9	Year 10+
Trastuz.	2·89 (537 / 18585)	1·54 (224 / 14499)	0·81 (33 / 4053)
Control	3·91 (466 / 11908)	2·39 (202 / 8465)	1·03 (21 / 2034)
Rate ratio, from	0·72 CI 0·65 – 0·81	0·64 CI 0·54 – 0·75	0·79 CI 0·47 – 1·32
(O–E) / V	–72·5 / 222·8	–42·6 / 94·8	–2·7 / 11·6

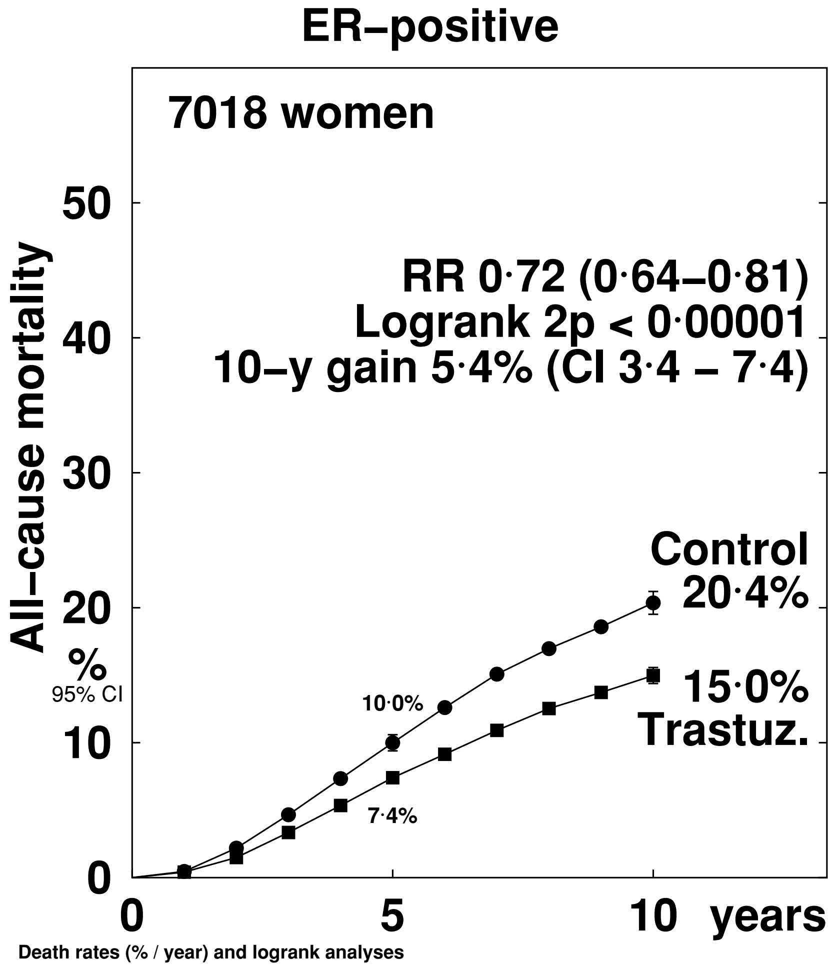

Death rates (% / year) and logrank analyses

Allocation	Years 0 – 4	Years 5 – 9	Year 10+
Trastuz.	1·57 (310 / 19734)	1·78 (288 / 16148)	1·62 (76 / 4677)
Control	2·12 (273 / 12859)	2·63 (256 / 9733)	2·12 (51 / 2408)
Rate ratio, from	0·73 CI 0·63 – 0·84	0·70 CI 0·60 – 0·81	0·81 CI 0·58 – 1·14
(O–E) / V	–41·4 / 131·5	–44·5 / 122·9	–5·8 / 28·2

P14: Any recurrence, excluding contralateral, by time period for ER-positive tumours split by PR-status

ER+ PR−

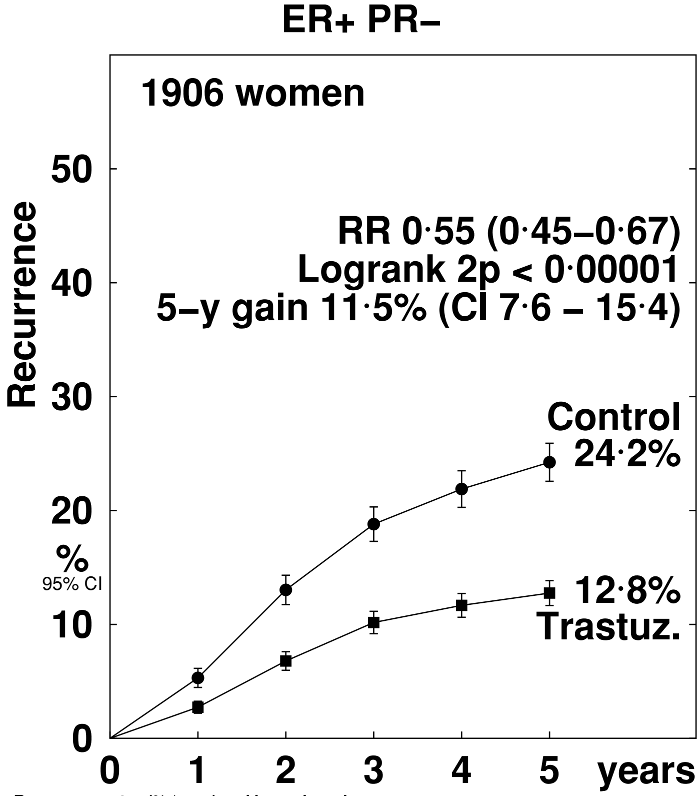

Recurrence rates (% / year) and logrank analyses

Allocation	Years 0 – 1	Years 2 – 4	Year 5+
Trastuz.	3·88 (83 / 2138)	2·48 (70 / 2824)	1·35 (67 / 4957)
Control	7·14 (103 / 1443)	5·02 (87 / 1733)	2·04 (55 / 2702)
Rate ratio, from	0·51 CI 0·41 – 0·63	0·49 CI 0·39 – 0·62	0·72 CI 0·52 – 0·99
(O–E) / V	−27·6 / 40·6	−24·6 / 34·4	−8·9 / 27·3

ER+ PR+

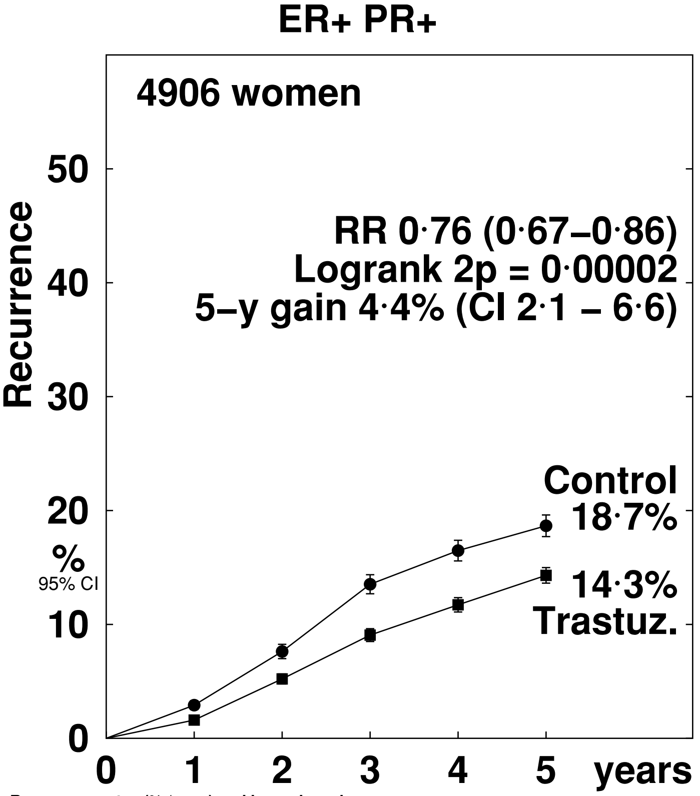

Recurrence rates (% / year) and logrank analyses

Allocation	Years 0 – 1	Years 2 – 4	Year 5+
Trastuz.	2·87 (163 / 5677)	3·45 (259 / 7513)	1·36 (181 / 13290)
Control	3·95 (144 / 3642)	4·51 (205 / 4548)	1·75 (130 / 7418)
Rate ratio, from	0·67 CI 0·55 – 0·82	0·78 CI 0·66 – 0·93	0·81 CI 0·65 – 1·00
(O–E) / V	−26·5 / 66·8	−25·0 / 100·9	−14·8 / 69·0

Category	Events/Women Allocated Trastuz.	Events/Women Allocated control	Trastuz. events Logrank O−E	Trastuz. events Variance of O−E	Ratio (& CI)
ER+ PR−	220/1126 (19·5%)	365/1164 (31·4%)	−61·1	102·3	0·55 (0·43 – 0·71)
ER+ PR+	603/2957 (20·4%)	713/2911 (24·5%)	−66·4	236·8	0·76 (0·64 – 0·89)
**Total**	**823/ 4083 (20·2%)**	**1078/ 4075 (26·5%)**	**−127·5**	**339·1**	**0·687 (0·617 – 0·764)** 2p < 0·00001

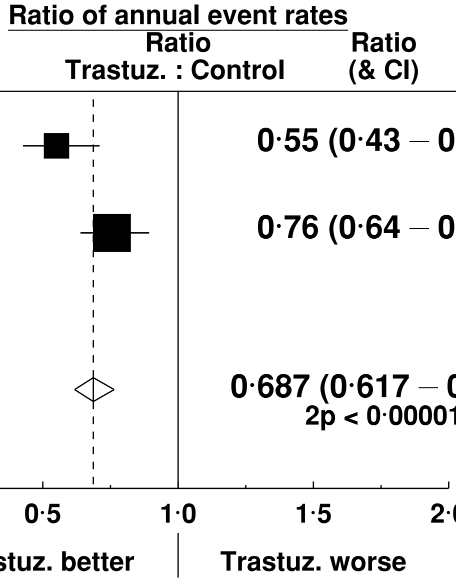

99% or 95% confidence intervals

Difference between treatment effects in 2 categories: $\chi^2_1 = 7{\cdot}2$; 2p = 0·007

Treatment effect 2p < 0·00001

P15: Sub-set analyses of trastuzumab versus control trials; any recurrence (analyses (b-l) and the overall total include any loco-regional or distant recurrence, but ignore contralateral disease)

Category	Events/Women: Allocated Trastuz.	Events/Women: Allocated control	Trastuz. events: Logrank O−E	Trastuz. events: Variance of O−E	Ratio (& CI)
**(a) Site of first recurrence** (χ^2_2 = 10.4; p = 0.006)					
**Distant**	1378/8299 (16·6%)	1899/8328 (22·8%)	−259·4	562·2	0·63 (0·57 − 0·70)
**Isolated local**	385/8299 (4·6%)	478/8328 (5·7%)	−50·5	155·1	0·72 (0·59 − 0·89)
**Contralateral**	203/8299 (2·4%)	191/8328 (2·3%)	−5·5	71·2	0·93 (0·68 − 1·26)
**(b) Follow up period, years** (trend χ^2_1 = 16.2; 2p = 0.00005)					
**0–1**	720/15575 (4·6%)	1193/14882 (8·0%)	−195·3	307·9	0·53 (0·46 − 0·61)
**2–4**	678/20066 (3·4%)	790/17927 (4·4%)	−82·6	258·3	0·73 (0·62 − 0·85)
**5–9**	332/27567 (1·2%)	359/23755 (1·5%)	−28·0	125·2	0·80 (0·64 − 1·01)
**10+**	40/7756 (0·5%)	48/6477 (0·7%)	−6·7	15·5	0·65 (0·34 − 1·25)
**(c) Age at entry** (trend χ^2_1 = 0.3; 2p > 0.1; NS)					
**Age < 45**	674/2767 (24·4%)	859/2727 (31·5%)	−105·9	259·3	0·66 (0·57 − 0·78)
**45 − 54**	571/2951 (19·3%)	809/3038 (26·6%)	−115·4	239·5	0·62 (0·52 − 0·73)
**55 − 69**	486/2380 (20·4%)	661/2368 (27·9%)	−81·8	194·5	0·66 (0·55 − 0·79)
**70+**	39/201 (19·4%)	61/195 (31·3%)	−9·5	13·6	0·50 (0·25 − 1·00)
**(d) ER/PR status** (χ^2_2 = 8.4; p = 0.01)					
**ER− PR any**	919/4098 (22·4%)	1259/4093 (30·8%)	−179·7	390·6	0·63 (0·55 − 0·72)
**ER+ PR−**	220/1126 (19·5%)	365/1164 (31·4%)	−61·1	102·3	0·55 (0·43 − 0·71)
**ER+ PR?**	27/116 (23·3%)	50/152 (32·9%)	−5·6	13·0	
**ER+ PR+**	603/2957 (20·4%)	713/2911 (24·5%)	−66·4	236·8	0·76 (0·64 − 0·89)
**(e) Nodal status** (trend χ^2_1 = 0.2; 2p > 0.1; NS)					
**N0**	205/1735 (11·8%)	291/1783 (16·3%)	−32·3	80·4	0·67 (0·50 − 0·89)
**N1–3**	512/3122 (16·4%)	740/3119 (23·7%)	−113·4	228·4	0·61 (0·51 − 0·72)
**N4+**	871/2783 (31·3%)	1126/2773 (40·6%)	−137·6	338·1	0·67 (0·58 − 0·77)
**N unknown**	182/659 (27·6%)	233/653 (35·7%)	−29·2	60·0	0·61 (0·44 − 0·86)
**(f) Tumour diameter** (trend χ^2_1 = 0.1; 2p > 0.1; NS)					
**1 − 10mm (T1a+b)**	68/544 (12·5%)	94/551 (17·1%)	−13·9	28·5	0·61 (0·38 − 0·99)
**11 − 20mm (T1c)**	442/2639 (16·7%)	648/2786 (23·3%)	−81·0	189·4	0·65 (0·54 − 0·79)
**21 − 50mm (T2)**	888/3995 (22·2%)	1212/3926 (30·9%)	−167·4	365·3	0·63 (0·55 − 0·72)
**> 50mm (T3/T4)**	198/590 (33·6%)	224/548 (40·9%)	−24·6	65·5	0·69 (0·50 − 0·94)
**Other / unknown**	174/531 (32·8%)	212/517 (41·0%)	−26·4	58·0	0·63 (0·45 − 0·89)
**(g) Tumour grade** (trend χ^2_1 = 0.0; 2p > 0.1; NS)					
**Well−differentiated**	29/170 (17·1%)	33/182 (18·1%)	−2·1	8·8	
**Moderately**	506/2419 (20·9%)	698/2486 (28·1%)	−91·5	210·2	0·65 (0·54 − 0·77)
**Poorly**	1136/5322 (21·3%)	1517/5274 (28·8%)	−194·6	461·7	0·66 (0·58 − 0·74)
**Grade unknown**	99/388 (25·5%)	142/386 (36·8%)	−21·3	41·0	0·60 (0·40 − 0·89)
**(h) Histological type** (χ^2_1 = 0.0; 2p > 0.1: NS)					
**Ductal**	1484/7210 (20·6%)	2038/7303 (27·9%)	−269·9	602·2	0·64 (0·58 − 0·71)
**Lobular**	73/253 (28·9%)	94/238 (39·5%)	−7·4	17·5	0·65 (0·35 − 1·21)
**Unknown**	213/836 (25·5%)	258/787 (32·8%)	−28·3	65·2	0·65 (0·47 − 0·89)
**(i) BMI** (trend χ^2_1 = 0.6; 2p > 0.1; NS)					
**<20**	117/549 (21·3%)	149/542 (27·5%)	−9·9	38·8	0·77 (0·51 − 1·17)
**20 − <25**	603/3005 (20·1%)	838/3050 (27·5%)	−122·3	237·9	0·60 (0·51 − 0·71)
**25 − <30**	559/2567 (21·8%)	768/2505 (30·7%)	−103·0	210·3	0·61 (0·51 − 0·73)
**30+**	486/2131 (22·8%)	617/2150 (28·7%)	−62·8	178·3	0·70 (0·58 − 0·85)
**Unknown**	5/47 (10·6%)	18/81 (22·2%)	−0·5	0·2	
**(j) Trastuzumab duration** (trend χ^2_1 = 0.2; 2p > 0.1; NS)					
**<12 months**	33/115 (28·7%)	38/116 (32·8%)	−3·3	13·1	
**12 months**	1328/6484 (20·5%)	1827/6515 (28·0%)	−282·6	641·1	0·64 (0·58 − 0·71)
**>12 months**	409/1700 (24·1%)	525/1697 (30·9%)	−80·8	210·7	0·68 (0·57 − 0·81)
**(k) Chemotherapy schedule** (χ^2_1 = 3.0; 2p = 0.08)					
**Concurrent**	640/3421 (18·7%)	929/3434 (27·1%)	−178·3	356·8	0·61 (0·53 − 0·70)
**Sequential**	1130/4878 (23·2%)	1461/4894 (29·9%)	−161·1	428·7	0·69 (0·61 − 0·78)
**(l) HER2:CEP17 ratio** (trend χ^2_1 = 2.6; 2p > 0.1; NS)					
**0 − <2.0**	44/205 (21·5%)	51/235 (21·7%)	1·6	11·2	
**2.0 − <3.5**	122/541 (22·6%)	151/524 (28·8%)	−9·5	39·6	0·79 (0·52 − 1·19)
**3.5 − <5.0**	134/623 (21·5%)	199/686 (29·0%)	−13·9	47·4	0·75 (0·51 − 1·08)
**5.0 − <7.5**	228/1207 (18·9%)	353/1266 (27·9%)	−48·8	88·7	0·58 (0·44 − 0·76)
**7.5 − <10.0**	158/918 (17·2%)	227/916 (24·8%)	−32·1	58·6	0·58 (0·41 − 0·81)
**10.0+**	185/929 (19·9%)	289/1063 (27·2%)	−30·5	74·9	0·67 (0·49 − 0·90)
**Unknown**	899/3876 (23·2%)	1120/3638 (30·8%)	−179·5	386·6	0·63 (0·55 − 0·72)
**Total**	**1770/8299 (21·3%)**	**2390/8328 (28·7%)**	**−312·6**	**706·9**	**0·643 (0·597 − 0·692)** 2p < 0·00001

Ratio of annual event rates — Ratio Trastuz. : Control

99% or ◇ 95% confidence intervals

0 0·5 1·0 1·5 2·0

Trastuz. better | Trastuz. worse

Treatment effect 2p < 0·00001

P16: Sub-set analyses of trastuzumab versus control trials; distant recurrence at any time

Category	Events/Women: Allocated Trastuz.	Events/Women: Allocated control	Trastuz. events: Logrank O–E	Trastuz. events: Variance of O–E	Ratio of annual event rates: Ratio (& CI)
**(a) Follow up period, years (trend χ^2_1 = 10.4; 2p = 0.001)**					
0–1	614/15676 (3·9%)	1064/15054 (7·1%)	−177·2	270·1	0·52 (0·44 – 0·61)
2–4	601/20618 (2·9%)	692/18627 (3·7%)	−68·5	229·4	0·74 (0·63 – 0·88)
5–9	297/28937 (1·0%)	349/25287 (1·4%)	−36·5	116·3	0·73 (0·58 – 0·93)
10+	28/8273 (0·3%)	41/6995 (0·6%)	−7·8	11·8	0·52 (0·24 – 1·09)
**(b) Age at entry (trend χ^2_1 = 0.0; 2p > 0.1; NS)**					
Age < 45	568/2767 (20·5%)	758/2727 (27·8%)	−100·2	225·9	0·64 (0·54 – 0·76)
45 – 54	501/2951 (17·0%)	734/3038 (24·2%)	−108·8	214·5	0·60 (0·51 – 0·72)
55 – 69	434/2380 (18·2%)	598/2368 (25·3%)	−72·8	174·2	0·66 (0·54 – 0·80)
70+	37/201 (18·4%)	56/195 (28·7%)	−8·2	13·0	0·53 (0·26 – 1·09)
**(c) ER/PR status (χ^2_2 = 7.6; p = 0.02)**					
ER− PR any	801/4098 (19·5%)	1119/4093 (27·3%)	−155·5	336·7	0·63 (0·55 – 0·72)
ER+ PR−	191/1126 (17·0%)	340/1164 (29·2%)	−60·1	93·2	0·52 (0·40 – 0·69)
ER+ PR?	23/116 (19·8%)	42/152 (27·6%)	−4·4	11·0	
ER+ PR+	524/2957 (17·7%)	645/2911 (22·2%)	−65·5	210·7	0·73 (0·61 – 0·88)
**(d) Nodal status (trend χ^2_1 = 1.0; 2p > 0.1; NS)**					
N0	153/1787 (8·6%)	226/1805 (12·5%)	−28·1	62·9	0·64 (0·46 – 0·89)
N1–3	423/3140 (13·5%)	652/3143 (20·7%)	−108·3	196·6	0·58 (0·48 – 0·69)
N4+	824/2793 (29·5%)	1086/2795 (38·9%)	−132·3	324·9	0·67 (0·58 – 0·77)
N unknown	140/579 (24·2%)	182/585 (31·1%)	−21·2	43·2	0·61 (0·41 – 0·91)
**(e) Tumour diameter (trend χ^2_1 = 2.4; 2p = 0.10)**					
1 – 20 mm (T1)	399/3183 (12·5%)	637/3337 (19·1%)	−98·6	183·8	0·58 (0·48 – 0·71)
21 – 50 mm (T2)	788/3995 (19·7%)	1106/3926 (28·2%)	−160·5	332·5	0·62 (0·54 – 0·71)
> 50 mm (T3/T4)	193/590 (32·7%)	208/548 (38·0%)	−18·0	63·9	0·75 (0·55 – 1·04)
Other / unknown	160/531 (30·1%)	195/517 (37·7%)	−17·3	52·0	0·72 (0·50 – 1·03)
**(f) Tumour grade (trend χ^2_1 = 0.0; 2p > 0.1; NS)**					
Well−differentiated	23/170 (13·5%)	27/182 (14·8%)	−2·5	6·8	
Moderately	434/2419 (17·9%)	610/2486 (24·5%)	−83·3	183·9	0·64 (0·53 – 0·77)
Poorly	997/5322 (18·7%)	1377/5274 (26·1%)	−184·8	415·9	0·64 (0·57 – 0·73)
Grade unknown	86/388 (22·2%)	132/386 (34·2%)	−15·8	34·5	0·63 (0·41 – 0·98)
**(g) Ki−67 (trend χ^2_1 = 0.1; 2p > 0.1; NS)**					
<10%	26/111 (23·4%)	38/131 (29·0%)	−2·3	6·6	
10 – <20%	33/169 (19·5%)	48/165 (29·1%)	−4·2	9·1	
20+%	74/343 (21·6%)	93/346 (26·9%)	−6·0	21·8	
Unknown	1407/7676 (18·3%)	1967/7686 (25·6%)	−271·2	578·6	0·63 (0·56 – 0·70)
**(h) Histological type (χ^2_1 = 0.0; 2p > 0.1; NS)**					
Ductal	1290/7210 (17·9%)	1828/7303 (25·0%)	−254·2	535·8	0·62 (0·56 – 0·70)
Lobular	66/253 (26·1%)	90/238 (37·8%)	−7·3	17·0	0·65 (0·35 – 1·21)
Unknown	184/836 (22·0%)	228/787 (29·0%)	−20·1	55·1	0·69 (0·49 – 0·98)
**(i) BMI (trend χ^2_1 = 1.8; 2p > 0.1; NS)**					
<20	90/549 (16·4%)	134/542 (24·7%)	−14·0	32·3	0·65 (0·41 – 1·02)
20 – <25	511/3005 (17·0%)	726/3050 (23·8%)	−107·2	203·3	0·59 (0·49 – 0·71)
25 – <30	495/2567 (19·3%)	700/2505 (27·9%)	−97·0	189·8	0·60 (0·50 – 0·72)
30+	440/2131 (20·6%)	571/2150 (26·6%)	−57·3	163·9	0·70 (0·58 – 0·86)
Unknown	4/47 (8·5%)	15/81 (18·5%)	−0·5	0·2	
**(j) Trastuzumab duration (trend χ^2_1 = 0.2; 2p > 0.1; NS)**					
<12 months	28/115 (24·3%)	36/116 (31·0%)	−5·0	11·6	
12 months	1158/6484 (17·9%)	1640/6515 (25·2%)	−258·6	570·1	0·64 (0·57 – 0·71)
>12 months	354/1700 (20·8%)	470/1697 (27·7%)	−77·2	188·0	0·66 (0·55 – 0·80)
**(k) Chemotherapy schedule (χ^2_1 = 2.6; 2p > 0.1; NS)**					
Concurrent	550/3421 (16·1%)	835/3434 (24·3%)	−162·8	313·8	0·60 (0·51 – 0·69)
Sequential	990/4878 (20·3%)	1311/4894 (26·8%)	−152·0	383·9	0·67 (0·59 – 0·77)
**(l) HER2:CEP17 ratio (trend χ^2_1 = 4.0; 2p = 0.05)**					
0 – <2.0	43/205 (21·0%)	45/235 (19·1%)	2·1	11·3	
2.0 – <3.5	109/541 (20·1%)	139/524 (26·5%)	−7·4	36·0	0·81 (0·53 – 1·25)
3.5 – <5.0	115/623 (18·5%)	182/686 (26·5%)	−15·6	42·0	0·69 (0·46 – 1·03)
5.0 – <7.5	193/1207 (16·0%)	324/1266 (25·6%)	−51·5	79·2	0·52 (0·39 – 0·70)
7.5 – <10.0	135/918 (14·7%)	180/916 (19·7%)	−23·8	48·7	0·61 (0·42 – 0·89)
10.0+	156/929 (16·8%)	271/1063 (25·5%)	−32·7	67·4	0·62 (0·45 – 0·84)
Unknown	789/3876 (20·4%)	1005/3638 (27·6%)	−160·6	343·1	0·63 (0·54 – 0·72)
**Total**	**1540/ 8299 (18·6%)**	**2146/ 8328 (25·8%)**	**−289·9**	**627·6**	**0·630 (0·583 – 0·681)** 2p < 0·00001

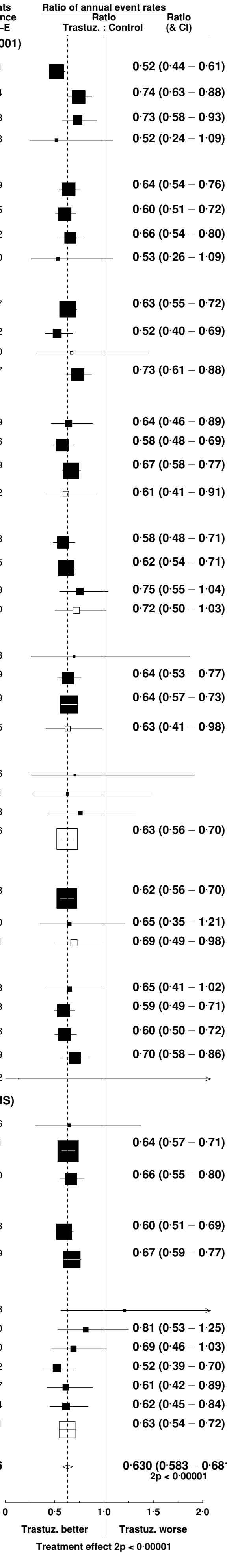

99% or 95% confidence intervals

P17: Sub-set analyses of trastuzumab versus control trials; breast cancer mortality

Category	Events/Women: Allocated Trastuz.	Events/Women: Allocated control	Trastuz. events: Logrank O–E	Trastuz. events: Variance of O–E	Ratio (& CI)
**(a) Follow up period, years** (trend $\chi^2_1 = 0.5$; 2p > 0.1; NS)					
**0–1**	169/16126 (1·0%)	338/15823 (2·1%)	−61·1	83·8	**0·48 (0·36 – 0·64)**
**2–4**	596/22134 (2·7%)	679/21151 (3·2%)	−46·7	226·2	**0·81 (0·69 – 0·97)**
**5–9**	425/30784 (1·4%)	602/27996 (2·2%)	−83·3	179·8	**0·63 (0·52 – 0·76)**
**10+**	66/8688 (0·8%)	87/7532 (1·2%)	−10·9	24·6	**0·64 (0·38 – 1·08)**
**(b) Age at entry** (trend $\chi^2_1 = 0.2$; 2p > 0.1; NS)					
**Age < 45**	437/2767 (15·8%)	560/2727 (20·5%)	−59·7	175·4	**0·71 (0·59 – 0·86)**
**45 – 54**	418/2951 (14·2%)	590/3038 (19·4%)	−78·0	177·5	**0·64 (0·53 – 0·78)**
**55 – 69**	363/2380 (15·3%)	507/2368 (21·4%)	−60·2	148·9	**0·67 (0·54 – 0·82)**
**70+**	38/201 (18·9%)	49/195 (25·1%)	−4·1	12·5	
**(c) ER/PR status** ($\chi^2_2 = 6.6$; p = 0.04)					
**ER− PR any**	699/4098 (17·1%)	946/4093 (23·1%)	−115·5	294·8	**0·68 (0·58 – 0·79)**
**ER+ PR−**	152/1126 (13·5%)	268/1164 (23·0%)	−44·4	73·8	**0·55 (0·41 – 0·74)**
**ER+ PR?**	16/116 (13·8%)	36/152 (23·7%)	−4·6	8·7	
**ER+ PR+**	388/2957 (13·1%)	456/2911 (15·7%)	−37·1	153·7	**0·79 (0·64 – 0·97)**
**(d) Nodal status** (trend $\chi^2_1 = 1.5$; 2p > 0.1; NS)					
**N0**	118/1787 (6·6%)	176/1805 (9·8%)	−21·8	49·2	**0·64 (0·44 – 0·93)**
**N1–3**	327/3140 (10·4%)	476/3143 (15·1%)	−70·5	148·2	**0·62 (0·50 – 0·77)**
**N4+**	695/2793 (24·9%)	895/2795 (32·0%)	−93·4	278·4	**0·71 (0·61 – 0·83)**
**N unknown**	116/579 (20·0%)	159/585 (27·2%)	−16·2	38·4	**0·66 (0·43 – 0·99)**
**(e) Tumour diameter** (trend $\chi^2_1 = 4.4$; 2p = 0.04)					
**1 – 20 mm (T1)**	313/3183 (9·8%)	496/3337 (14·9%)	−75·6	143·8	**0·59 (0·48 – 0·73)**
**21 – 50 mm (T2)**	647/3995 (16·2%)	867/3926 (22·1%)	−106·0	270·6	**0·68 (0·58 – 0·79)**
**> 50 mm (T3/T4)**	169/592 (28·5%)	171/548 (31·2%)	−11·4	57·1	**0·82 (0·58 – 1·15)**
**Other / unknown**	127/529 (24·0%)	172/517 (33·3%)	−17·4	45·0	**0·68 (0·46 – 1·00)**
**(f) Tumour grade** (trend $\chi^2_1 = 0.0$; 2p > 0.1; NS)					
**Well−differentiated**	16/170 (9·4%)	20/182 (11·0%)	−3·0	4·1	
**Moderately**	332/2419 (13·7%)	441/2486 (17·7%)	−50·4	137·3	**0·69 (0·56 – 0·86)**
**Poorly**	848/5322 (15·9%)	1144/5274 (21·7%)	−138·1	355·8	**0·68 (0·59 – 0·78)**
**Grade unknown**	60/388 (15·5%)	101/386 (26·2%)	−10·8	26·1	**0·66 (0·40 – 1·10)**
**(g) Ki−67** (trend $\chi^2_1 = 0.0$; 2p > 0.1; NS)					
**<10%**	21/111 (18·9%)	30/131 (22·9%)	−1·6	5·6	
**10 – <20%**	28/169 (16·6%)	39/165 (23·6%)	−2·2	8·3	
**20+%**	55/343 (16·0%)	66/346 (19·1%)	−4·8	15·7	
**Unknown**	1152/7676 (15·0%)	1571/7686 (20·4%)	−190·7	475·6	**0·67 (0·60 – 0·75)**
**(h) Histological type** ($\chi^2_1 = 0.4$; 2p > 0.1: NS)					
**Ductal**	1054/7210 (14·6%)	1460/7303 (20·0%)	−184·3	439·8	**0·66 (0·58 – 0·74)**
**Lobular**	49/253 (19·4%)	62/238 (26·1%)	−2·9	11·9	
**Unknown**	153/836 (18·3%)	184/787 (23·4%)	−20·4	49·9	**0·67 (0·46 – 0·96)**
**(i) BMI** (trend $\chi^2_1 = 1.3$; 2p > 0.1; NS)					
**<20**	64/549 (11·7%)	108/542 (19·9%)	−11·3	25·2	**0·64 (0·38 – 1·07)**
**20 – <25**	412/3005 (13·7%)	542/3050 (17·8%)	−66·3	158·7	**0·66 (0·54 – 0·81)**
**25 – <30**	412/2567 (16·0%)	579/2505 (23·1%)	−75·7	159·5	**0·62 (0·51 – 0·76)**
**30+**	366/2131 (17·2%)	462/2150 (21·5%)	−37·7	137·2	**0·76 (0·61 – 0·95)**
**Unknown**	2/47 (4·3%)	15/81 (18·5%)	−0·5	0·2	
**(j) Trastuzumab duration** (trend $\chi^2_1 = 0.0$; 2p > 0.1; NS)					
**<12 months**	22/115 (19·1%)	26/116 (22·4%)	−3·6	8·5	
**12 months**	948/6484 (14·6%)	1297/6515 (19·9%)	−177·0	467·4	**0·68 (0·61 – 0·77)**
**>12 months**	286/1700 (16·8%)	383/1697 (22·6%)	−59·4	156·9	**0·68 (0·56 – 0·84)**
**(k) Chemotherapy schedule** ($\chi^2_1 = 3.4$; 2p = 0.07)					
**Concurrent**	441/3421 (12·9%)	658/3434 (19·2%)	−118·1	253·0	**0·63 (0·53 – 0·74)**
**Sequential**	815/4878 (16·7%)	1048/4894 (21·4%)	−99·6	318·7	**0·73 (0·63 – 0·85)**
**(l) HER2:CEP17 ratio** (trend $\chi^2_1 = 2.8$; 2p = 0.10)					
**0 – <2.0**	35/205 (17·1%)	42/235 (17·9%)	−0·9	8·7	
**2.0 – <3.5**	91/541 (16·8%)	101/524 (19·3%)	−0·9	28·2	
**3.5 – <5.0**	91/623 (14·6%)	147/686 (21·4%)	−11·1	33·7	**0·72 (0·46 – 1·12)**
**5.0 – <7.5**	147/1207 (12·2%)	256/1266 (20·2%)	−37·9	63·7	**0·55 (0·40 – 0·76)**
**7.5 – <10.0**	107/918 (11·7%)	158/916 (17·2%)	−23·3	39·9	**0·56 (0·37 – 0·84)**
**10.0+**	130/929 (14·0%)	214/1063 (20·1%)	−21·6	55·4	**0·68 (0·48 – 0·96)**
**Unknown**	655/8376 (7·8%)	788/3638 (21·7%)	−106·2	284·8	**0·69 (0·59 – 0·80)**
**Total**	**1256/ 8299 (15·1%)**	**1706/ 8328 (20·5%)**	**−201·9**	**514·3**	**0·675 (0·619 – 0·736)** 2p < 0·00001

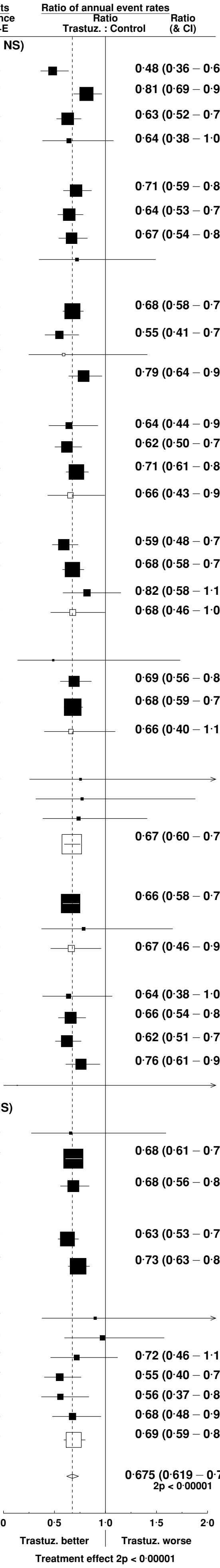

99% or 95% confidence intervals

P18: Brain recurrence as first event in trials of trastuzumab versus no trastuzumab

Year code and study name	Treatment comparison	Events/Women: Allocated Trastuz.	Events/Women: Adjusted control	Trastuz. events: Logrank O–E	Trastuz. events: Variance of O–E
**Trials of trastuzumab vs no trastuzumab**					
2000 NSABP B–31	4**AC q3**; (4**P**$_{175}$**q3** ± 12mths **Tr q1**)	28/1058	20/1061	3·3	11·8
2000 NCCTG/Int.N9831	4**AC q3**; (12**P**$_{80}$ **q1**; 12mths **Tr q1** vs not)	3/162	10/166	–3·7	2·8
2000 FinHer/FBCG 00–01	((3**D**$_{100}$**q3** v 9**Vin**$_{25}$**q1**) ± 9**Tr q1**); 3**F**$_{600}$**E**$_{60}$**C**$_{600}$	3/115	5/116	–1·1	1·3
2000 NCCTG/Int.N9831 *	4**AC q3**; (12**P**$_{80}$ **q1** ± 12mths **Tr q1** (Seq. or Concur.))	63/2111	2(30/1066)	–0·3	19·9
2001 HERA/BIG 01–01 *	Chemo; 12 or 24mths **Tr q3** vs not	76/3402	2(43/1697)	–5·4	24·7
2001 PACS 04 France	6(**F**$_{500}$**E**$_{100}$**C**$_{500}$ v **E**$_{75}$**D**$_{75}$); 12mths **Tr q3** vs not	9/260	9/268	0·7	4·4
2001 BCIRG 006	4**AC q3**; (4**D**$_{100}$**q3** ± 12mths **Tr q1**)	22/1074	22/1073	–1·3	10·5
2002 NOAH	neo(3**A**$_{60}$**P**$_{150}$**q3**; (4**P**$_{175}$**q3**;3**CMF q4**) ± 12mths **Tr q3**)	7/117	8/118	0·3	3·3
**■ Total**		**211/ 8299 (2·5%)**	**220/ 8328 (2·6%)**	**–7·5**	**78·7**

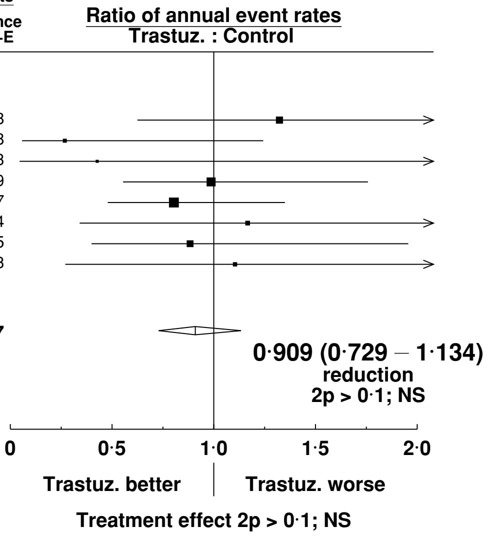

99% or 95% confidence intervals

Heterogeneity between 8 trials: χ^2_7 = 7·5; p > 0·1; NS

* For balance, control patients in 3–way trials or trial strata count half or twice in subtotal(s) and in final total of events/patients.

Distant recurrence outside the brain as first event in trials of trastuzmab versus no trastuzmab

Year started and study name	Treatment comparison	Events/Women: Allocated Trastuz.	Events/Women: Adjusted control	Trastuz. events: Logrank O–E	Trastuz. events: Variance of O–E
**Trials of trastuzumab vs no trastuzumab**					
2000 NSABP B–31	4**AC q3**; (4**P**$_{175}$**q3** ± 12mths **Tr q1**)	141/1058	264/1061	–67·8	95·9
2000 NCCTG/Int.N9831	4**AC q3**; (12**P**$_{80}$ **q1**; 12mths **Tr q1** vs not)	25/162	45/166	–10·1	15·4
2000 FinHer/FBCG 00–01	((3**D**$_{100}$**q3** v 9**Vin**$_{25}$**q1**) ± 9**Tr q1**); 3**F**$_{600}$**E**$_{60}$**C**$_{600}$	25/115	31/116	–3·9	10·3
2000 NCCTG/Int.N9831 *	4**AC q3**; (12**P**$_{80}$ **q1** ± 12mths **Tr q1** (Seq. or Concur.))	278/2111	2(202/1066)	–49·2	99·6
2001 HERA/BIG 01–01 *	Chemo; 12 or 24mths **Tr q3** vs not	634/3402	2(427/1697)	–96·2	212·4
2001 PACS 04 France	6(**F**$_{500}$**E**$_{100}$**C**$_{500}$ v **E**$_{75}$**D**$_{75}$); 12mths **Tr q3** vs not	52/260	75/268	–9·7	29·2
2001 BCIRG 006	4**AC q3**; (4**D**$_{100}$**q3** ± 12mths **Tr q1**)	142/1074	207/1073	–42·3	82·8
2002 NOAH	neo(3**A**$_{60}$**P**$_{150}$**q3**; (4**P**$_{175}$**q3**;3**CMF q4**) ± 12mths **Tr q3**)	32/117	46/118	–8·2	15·2
**■ Total**		**1329/ 8299 (16·0%)**	**1926/ 8328 (23·1%)**	**–287·4**	**560·7**

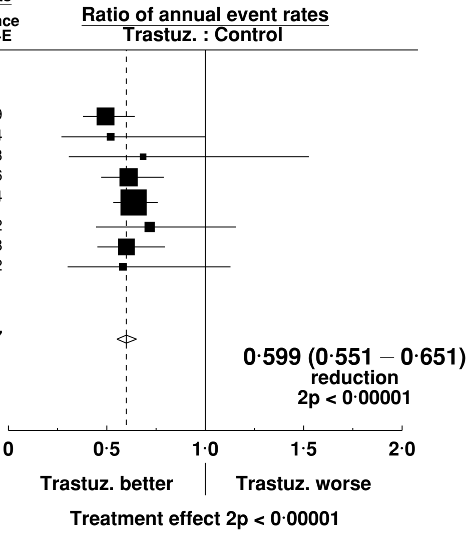

99% or 95% confidence intervals

Heterogeneity between 8 trials: χ^2_7 = 5·9; p > 0·1; NS

* For balance, control patients in 3–way trials or trial strata count half or twice in subtotal(s) and in final total of events/patients.

P19: Mortality by cause and incidence of second cancers

	Events		(O-E)	Variance	Rate Ratio	95% CI	p
	**Trastuzumab (n=8299)**	**Not (n=8328)**					
Death without recurrence	213	201	-8.2	75.5	0.90	0.72 - 1.12	0.35
Death with recurrence	1256	1706	-208.7	517.2	0.67	0.61 - 0.73	<0.00001
Any death	1469	1907	-2216.9	592.6	0.69	0.64 – 0.75	<0.00001
**Death without recurrence (selected groups of causes)**							
**Vascular disease:**	49	33	2.9	14.2	1.23	0.73 – 2.06	0.44
Stroke	13	6	1.7	3.3	1.70	0.57 – 5.05	0.34
Pulmonary embolism	6	6	-0.3	1.9	0.87	0.21 – 3.61	0.84
Heart & other vascular	30	21	1.4	9.1	1.17	0.61 – 2.25	0.63
**Cancers other than breast:**	65	68	-5.9	25.3	0.79	0.54 – 1.17	0.24
Lung cancer	14	8	1.5	4.5	1.39	0.55 – 3.51	0.49
AML	5	10	-3.0	2.9	0.36	0.11 – 1.14	0.08
Pancreatic cancer	6	9	-1.3	2.4	0.58	0.16 – 2.06	0.40
Other non-breast	40	41	-3.2	15.4	0.81	0.49 – 1.34	0.42
**Other specified cause**	59	68	-6.4	23.1	0.76	0.50 – 1.14	0.18
**Unknown cause**	40	32	1.3	13.0	1.10	0.64 – 1.90	0.72
**Death without recurrence by entry age** % (events/woman-years)							
<55	0.16 (79/49166)	0.21 (90/43473)	-7.2	31.2	0.80	0.56 – 1.13	0.20
55 – 69	0.48 (98/20524)	0.49 (91/18524)	-3.5	35.3	0.90	0.65 – 1.26	0.55
≥70	2.31 (36/1557)	1.37 (20/1464)	2.5	8.9	1.33	0.69 – 2.56	0.40
All ages	0.30 (213/71247)	0.32 (201/63461)	-8.9	75.5	0.89	0.71 – 1.11	0.31
**Death without recurrence during the first year* by chemotherapy administration** % (events/woman-years)							
Concurrent	0.34 (11/3251)	0.22 (7/3195)	2.2	4.4	1.65	0.65 – 4.20	0.29
Sequential	0.43 (20/4607)	0.13 (6/4468)	6.5	5.6	3.19	1.39 – 7.31	0.006
All women	0.39 (31/7889)	0.17 (13/7664)	6.8	8.9	2.15	1.11 – 4.14	0.02
**Second primary cancer as first event by entry age** % (events/woman-years)							
<55	0.32 (155/48593)	0.36 (155/43008)	-7.6	57.7	0.88	0.68 – 1.14	0.32
55 – 69	0.70 (140/20044)	0.69 (126/18170)	-1.4	50.4	0.97	0.74 – 1.28	0.85
≥70	1.20 (18/1505)	1.12 (16/1433)	-0.2	5.3	0.96	0.41 – 2.25	0.93
All ages	0.45 (313/70142)	0.47 (297/62611)	-9.1	113.5	0.92	0.77 – 1.11	0.39

*Break down of cause of death in first year

Cause	Trastuzumab	Control
Iatrogenic	7	3
Cardiac	4	3
Pulmonary embolism	3	0
Stroke	3	0
Other cancer, non-breast	3	2
Pneumonia & respiratory diseases	4	1
Infectious/sepsis	1	1
Other	3	3
Unknown not breast cancer	3	0
**Total**	**31**	**13**

P20: Any recurrence in trials of trastuzumab versus no trastuzumab, including TCH arm of BCIRG 006

Year started and study name	Treatment comparison	Events/Women Allocated Trastuz.	Events/Women Adjusted control	Trastuz. events Logrank O–E	Trastuz. events Variance of O–E
**(a) Trials of trastuzumab vs no trastuzumab**					
2000 NSABP B–31	4**AC q3**; (4**P**$_{175}$**q3** ± 12mths **Tr q1**)	221/1058	351/1061	−77·6	133·5
2000 NCCTG/Int.N9831	4**AC q3**; (12**P**$_{80}$ **q1**; 12mths **Tr q1** vs not)	33/162	60/166	−14·5	19·5
2000 FinHer/FBCG 00–01	((3**D**$_{100}$**q3** v 9**Vin**$_{25}$**q1**) ± 9**Tr q1**); 3**F**$_{600}$**E**$_{60}$**C**$_{600}$	36/115	39/116	−2·3	14·0
2000 NCCTG/Int.N9831 *	4**AC q3**; (12**P**$_{80}$ **q1** ± 12mths **Tr q1** (Seq. or Concur.))	454/2111	2(287/1066)	−51·7	151·2
2001 HERA/BIG 01–01 *	Chemo; 12 or 24mths **Tr q3** vs not	907/3402	2(563/1697)	−105·4	289·0
2001 PACS 04 France	6(**F**$_{500}$**E**$_{100}$**C**$_{500}$ v **E**$_{75}$**D**$_{75}$); 12mths **Tr q3** vs not	74/260	96/268	−8·7	38·4
2001 BCIRG 006	4**AC q3**; (4**D**$_{100}$**q3** ± 12mths **Tr q1**)	197/1074	261/1073	−46·2	106·5
2002 NOAH	neo(3**A**$_{60}$**P**$_{150}$**q3**; (4**P**$_{175}$**q3**;3**CMF q4**) ± 12mths **Tr q3**)	44/117	62/118	−9·6	19·5
**(a) subtotal**		**1966/ 8299 (23·7%)**	**2569/ 8328 (30·8%)**	**−316·1**	**771·6**
**(b) TCH arm of BCIRG 006**					
2001 BCIRG 006	(6**D**$_{75}$(**CisP**$_{75}$/**CarbP**$_{AUC6}$) + 18**Tr q1**; 11**Tr q3**) vs **AC**;4**D**$_{100}$	215/1075	261/1073	−34·2	110·8

Heterogeneity between 8 trials: $\chi^2_7 = 9{\cdot}7$; p > 0·1; NS

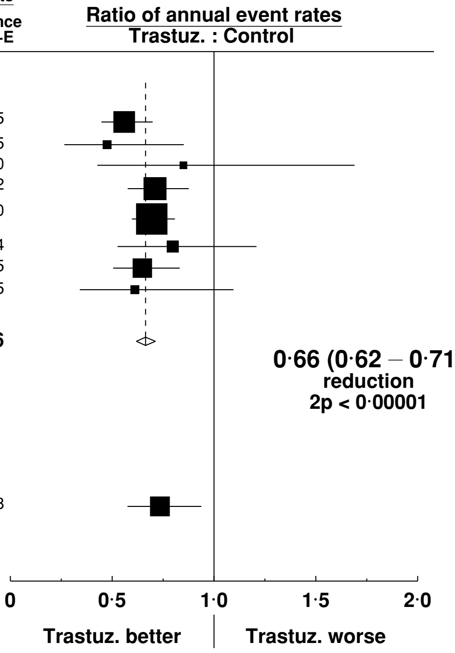

99% or 95% confidence intervals

* For balance, control patients in 3–way trials or trial strata count half or twice in subtotal(s).

Any recurrence in trials of trastuzumab versus no trastuzumab, including TCH arm of BCIRG 006, HER2:CEP17 ratio (unknowns excluded)

Category	Events/Women Allocated Trastuz.	Events/Women Allocated control	Trastuz. events Logrank O–E	Trastuz. events Variance of O–E	Ratio (& CI)
**HER2:CEP17 ratio (trend $\chi^2_1 = 3.1$; 2p = 0.08)**					
**0 – <2.0**	48/208 (23·1%)	65/235 (27·7%)	0·2	13·0	
**2.0 – <3.5**	161/658 (24·5%)	195/637 (30·6%)	−10·6	54·7	**0·82 (0·58 – 1·17)**
**3.5 – <5.0**	176/740 (23·8%)	235/797 (29·5%)	−13·7	63·0	**0·80 (0·58 – 1·11)**
**5.0 – <7.5**	306/1477 (20·7%)	442/1540 (28·7%)	−63·0	121·6	**0·60 (0·47 – 0·75)**
**7.5 – <10.0**	219/1149 (19·1%)	292/1129 (25·9%)	−42·1	84·3	**0·61 (0·46 – 0·80)**
**10.0+**	261/1229 (21·2%)	386/1386 (27·8%)	−40·5	110·9	**0·69 (0·54 – 0·89)**
**Total**	**1171/ 5461 (21·4%)**	**1615/ 5724 (28·2%)**	**−169·8**	**447·5**	**0·684 (0·624 – 0·751)** 2p < 0·00001

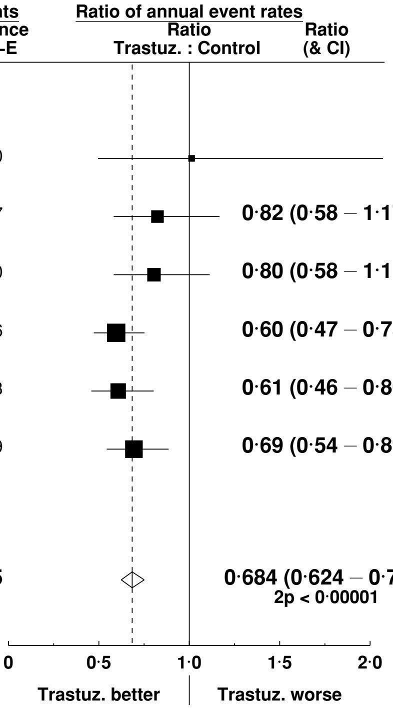

99% or 95% confidence intervals

P21: Sensitivity analyses accounting for crossover in HERA, all women censored on 30/06/2005. 10-year risk of (A) recurrence, (B) breast cancer mortality, (C) death without recurrence and (D) all-cause mortality in trials of trastuzumab versus no trastuzumab

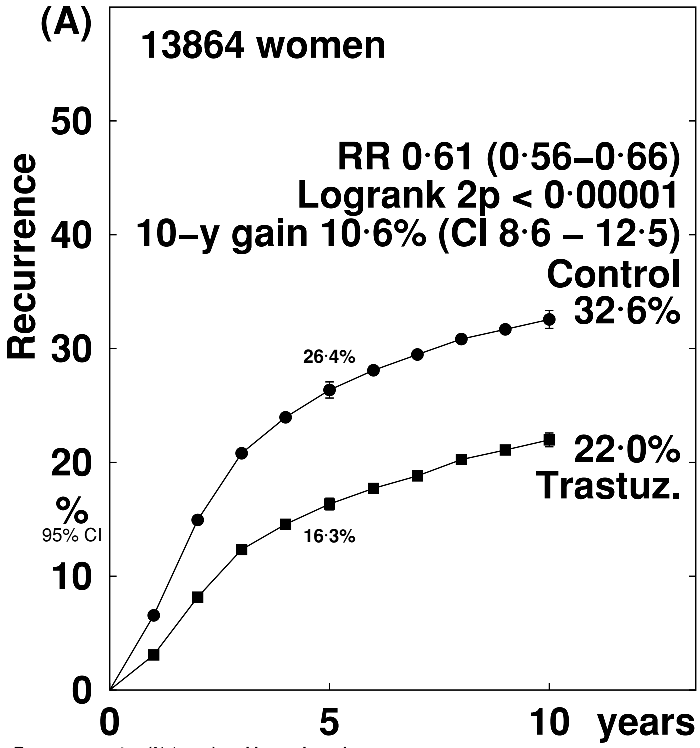

Recurrence rates (% / year) and logrank analyses

Allocation	Years 0 – 4	Years 5 – 9	Year 10+
Trastuz.	4·04 (1087 / 26902)	1·44 (234 / 16295)	1·16 (64 / 5515)
Control	6·65 (1207 / 18159)	1·88 (201 / 10669)	1·37 (39 / 2857)
Rate ratio, from	0·57 CI 0·53 – 0·61	0·78 CI 0·66 – 0·93	0·89 CI 0·60 – 1·32
(O–E) / V	–275·0 / 482·9	–24·0 / 98·6	–2·6 / 22·4

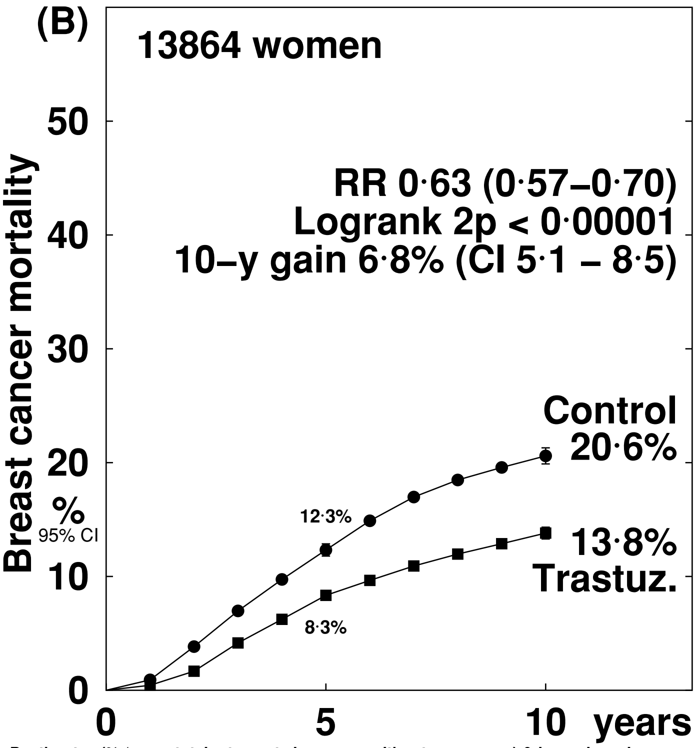

Death rates (% / year: total rate – rate in women without recurrence) & logrank analyses

Allocation	Years 0 – 4	Years 5 – 9	Year 10+
Trastuz.	1·63 CI 1·48 – 1·78	1·28 CI 1·12 – 1·45	0·82 CI 0·59 – 1·04
Control	2·57 CI 2·35 – 2·79	2·18 CI 1·93 – 2·44	1·09 CI 0·73 – 1·44
Rate ratio, from	0·64 CI 0·57 – 0·71	0·61 CI 0·53 – 0·71	0·73 CI 0·49 – 1·09
(O–E) / V	–98·2 / 218·0	–56·3 / 114·0	–5·6 / 18·1

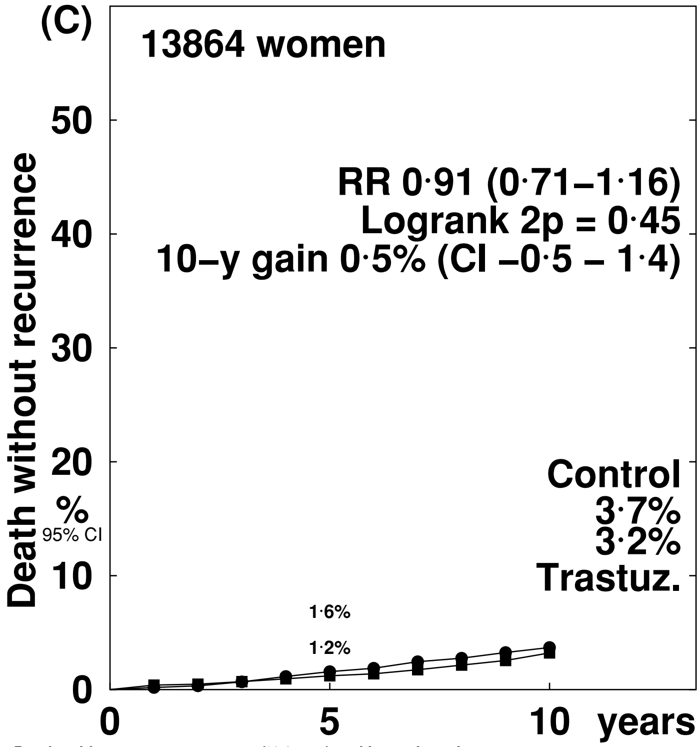

Death–without–recurrence rates (% / year) and logrank analyses

Allocation	Years 0 – 4	Years 5 – 9	Year 10+
Trastuz.	0·26 (69 / 26902)	0·42 (68 / 16295)	0·67 (37 / 5515)
Control	0·29 (52 / 18159)	0·40 (43 / 10669)	0·67 (19 / 2857)
Rate ratio, from	0·87 CI 0·62 – 1·23	0·91 CI 0·62 – 1·33	1·01 CI 0·56 – 1·81
(O–E) / V	–3·8 / 27·6	–2·3 / 24·3	0·1 / 11·4

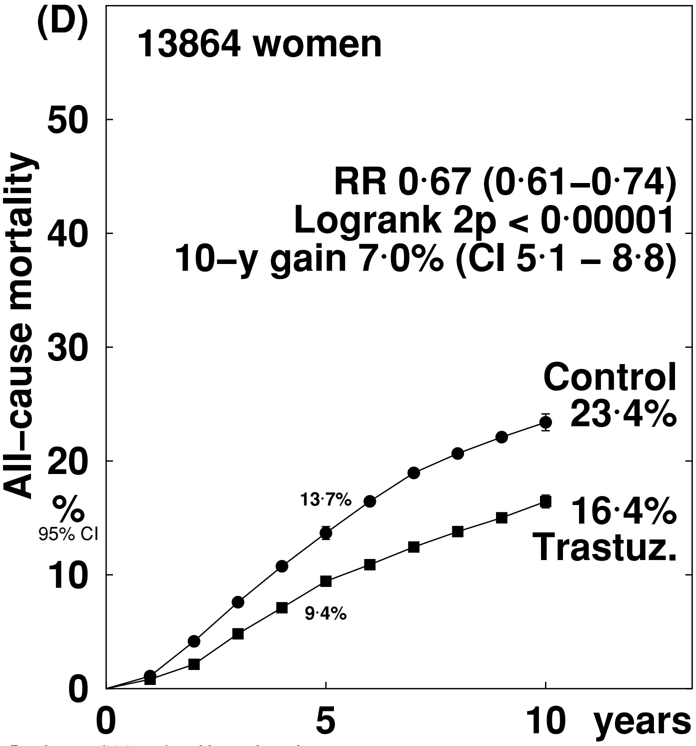

Death rates (% / year) and logrank analyses

Allocation	Years 0 – 4	Years 5 – 9	Year 10+
Trastuz.	1·88 (534 / 28371)	1·68 (298 / 17790)	1·43 (87 / 6075)
Control	2·85 (571 / 20028)	2·56 (317 / 12388)	1·66 (55 / 3309)
Rate ratio, from	0·66 CI 0·60 – 0·73	0·65 CI 0·57 – 0·75	0·83 CI 0·60 – 1·16
(O–E) / V	–102·0 / 245·6	–58·6 / 138·3	–5·5 / 29·5

P22: Comparison of recurrence reductions with sequential and concurrent trastuzumab and chemotherapy in all data and just in years 0-4

All data

Category	Events/Women: Allocated Trastuz.	Events/Women: Allocated control	Trastuz. events: Logrank O–E	Trastuz. events: Variance of O–E	Ratio of annual event rates: Ratio (& CI)
**Concurrent**	640/3421 (18·7%)	929/3434 (27·1%)	−178·3	356·8	**0·61 (0·53 – 0·70)**
**Sequential**	1130/4878 (23·2%)	1461/4894 (29·9%)	−161·1	428·7	**0·69 (0·61 – 0·78)**
**Total**	**1770/ 8299 (21·3%)**	**2390/ 8328 (28·7%)**	**−339·4**	**785·5**	**0·649 (0·605 – 0·696)** 2p < 0·00001

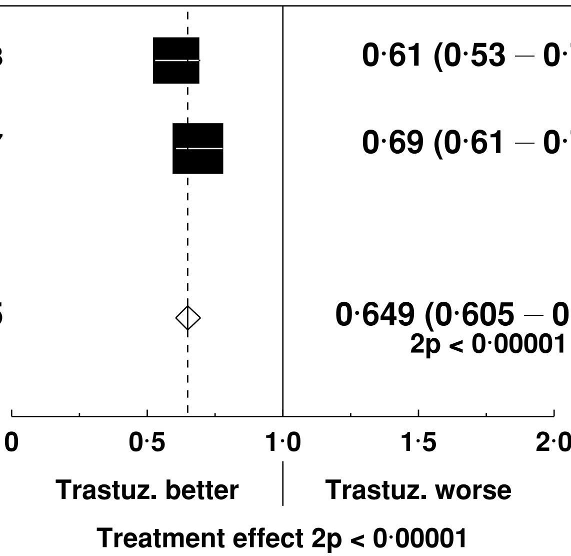

■ 99% or ◇ 95% confidence intervals

Difference between treatment effects in 2 categories: $\chi^2_1 = 3·0$; 2p = 0·08

Years 0-4

Category	Events/Women: Allocated Trastuz.	Events/Women: Allocated control	Trastuz. events: Logrank O–E	Trastuz. events: Variance of O–E	Ratio of annual event rates: Ratio (& CI)
**Concurrent**	482/3421 (14·1%)	769/3434 (22·4%)	−164·5	280·8	**0·56 (0·48 – 0·65)**
**Sequential**	916/4878 (18·8%)	1214/4894 (24·8%)	−137·1	345·3	**0·67 (0·59 – 0·77)**
**Total**	**1398/ 8299 (16·8%)**	**1983/ 8328 (23·8%)**	**−301·6**	**626·0**	**0·618 (0·571 – 0·668)** 2p < 0·00001

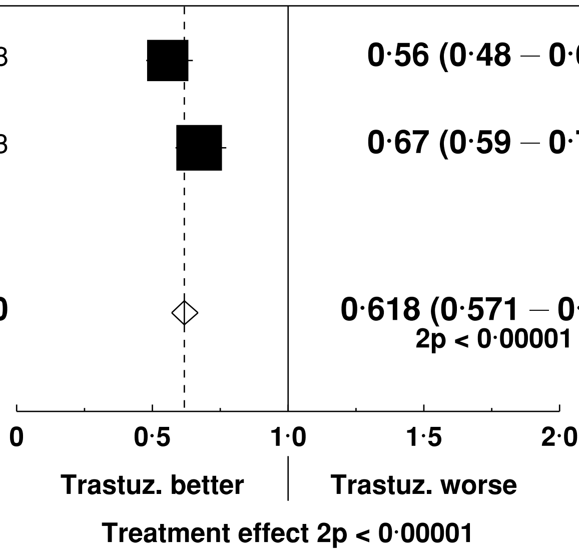

■ 99% or ◇ 95% confidence intervals

Difference between treatment effects in 2 categories: $\chi^2_1 = 5·5$; 2p = 0·02

P23: Direct randomised comparison of concurrent versus sequential chemotherapy and trastuzumab in the NCCTG 9831 trial: effect on (A) any recurrence, (B) distant recurrence at any time, and (C) breast cancer mortality

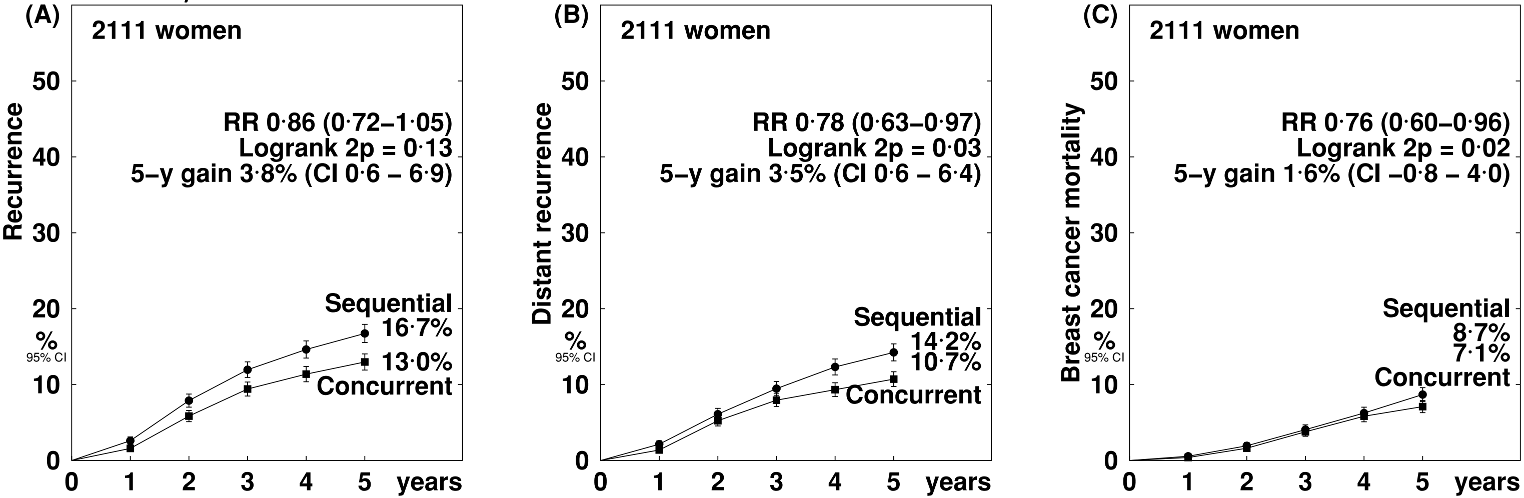

Recurrence rates (% / year) and logrank analyses

Allocation	Years 0 – 1	Years 2 – 4	Year 5+
Concurrent	2·94 (60 / 2040)	2·67 (73 / 2738)	1·34 (81 / 6023)
Sequential	4·13 (83 / 2011)	3·40 (89 / 2619)	1·16 (68 / 5850)
Rate ratio, from	0·73 CI 0·54 – 0·98	0·78 CI 0·59 – 1·03	1·13 CI 0·80 – 1·60
(O–E) / V	–10·4 / 32·7	–9·4 / 37·9	4·4 / 35·7

Distant recurrence rates (% / year) and logrank analyses

Allocation	Years 0 – 1	Years 2 – 4	Year 5+
Concurrent	2·59 (53 / 2045)	2·01 (56 / 2789)	0·65 (41 / 6323)
Sequential	3·21 (65 / 2024)	3·05 (82 / 2687)	0·72 (44 / 6088)
Rate ratio, from	0·85 CI 0·60 – 1·20	0·66 CI 0·50 – 0·87	0·92 CI 0·61 – 1·39
(O–E) / V	–4·5 / 27·2	–13·6 / 32·4	–1·8 / 20·9

Death rates (% / year: total rate – rate in women without recurrence) & logrank analyses

Allocation	Years 0 – 1	Years 2 – 4	Year 5+
Concurrent	0·77 CI 0·39 – 1·15	1·88 CI 1·39 – 2·38	0·83 CI 0·61 – 1·05
Sequential	1·02 CI 0·58 – 1·45	2·42 CI 1·85 – 2·99	1·18 CI 0·92 – 1·45
Rate ratio, from	0·83 CI 0·45 – 1·52	0·81 CI 0·58 – 1·13	0·69 CI 0·52 – 0·93
(O–E) / V	–1·6 / 8·7	–6·0 / 28·9	–11·3 / 31·0

P24: Direct randomised comparison of concurrent versus sequential chemotherapy and trastuzumab in the NCCTG 9831 trial: effect on (A) isolated local recurrence as first event, (B) contralateral breast cancer as first event, and (C) distant recurrence as first event

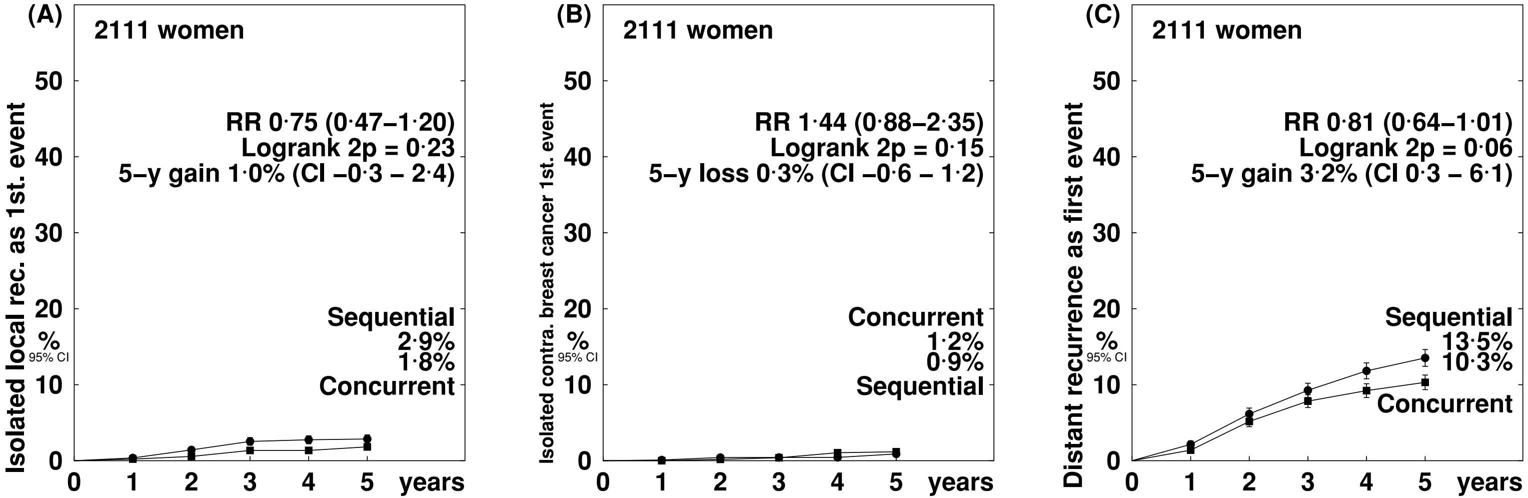

Local recurrence rates (% / year) and logrank analyses

Allocation	Years 0 – 1	Years 2 – 4	Year 5+
Concurrent	0·29 (6 / 2040)	0·44 (12 / 2738)	0·25 (15 / 6023)
Sequential	0·70 (14 / 2011)	0·50 (13 / 2619)	0·22 (13 / 5850)
Rate ratio, from	0·42 CI 0·23 – 0·77	0·86 CI 0·41 – 1·79	1·01 CI 0·46 – 2·19
(O–E) / V	–4·1 / 4·8	–0·9 / 6·1	0·0 / 6·4

Contralateral breast cancer as first event rates (% / year) and logrank analyses

Allocation	Years 0 – 1	Years 2 – 4	Year 5+
Concurrent	0·10 (2 / 2040)	0·33 (9 / 2738)	0·46 (28 / 6023)
Sequential	0·20 (4 / 2011)	0·15 (4 / 2619)	0·31 (18 / 5850)
Rate ratio, from	0·44 CI 0·14 – 1·33	2·15 CI 0·42 – 11·05	1·49 CI 0·72 – 3·05
(O–E) / V	–1·2 / 1·4	2·5 / 3·2	4·4 / 11·2

Distant recurrence as first event rates (% / year) and logrank analyses

Allocation	Years 0 – 1	Years 2 – 4	Year 5+
Concurrent	2·55 (52 / 2040)	1·90 (52 / 2738)	0·63 (38 / 6023)
Sequential	3·23 (65 / 2011)	2·75 (72 / 2619)	0·63 (37 / 5850)
Rate ratio, from	0·83 CI 0·59 – 1·17	0·69 CI 0·51 – 0·93	0·99 CI 0·63 – 1·57
(O–E) / V	–5·1 / 27·0	–10·9 / 29·1	–0·1 / 18·3

P25: Sensitivity analyses for death without recurrence (all trials), by tumour size and nodal status

Category	Events/Women: Allocated Trastuz.	Events/Women: Allocated control	Trastuz. events: Logrank O−E	Trastuz. events: Variance of O−E	Ratio of annual event rates: Ratio (& CI)
**(a) Tumour size (trend χ^2_1 = 0.1; 2p > 0.1; NS)**					
**1 – 20 mm (T1)**	78/3183 (2·5%)	89/3337 (2·7%)	−6·6	31·1	**0·81 (0·51 − 1·29)**
**21 – 50 mm (T2)**	116/3995 (2·9%)	87/3926 (2·2%)	4·8	39·1	
**> 50 mm (T3/T4)**	12/590 (2·0%)	18/548 (3·3%)	−3·0	4·6	
**Other / unknown**	7/531 (1·3%)	7/517 (1·4%)	−0·3	2·3	
**(b) Nodal status (trend χ^2_1 = 0.3; 2p > 0.1; NS)**					
**N0**	33/1735 (1·9%)	28/1783 (1·6%)	−0·3	10·7	
**N1−3**	84/3122 (2·7%)	77/3119 (2·5%)	−2·5	31·1	
**N4+**	80/2783 (2·9%)	82/2773 (3·0%)	−5·8	28·9	
**N unknown**	16/659 (2·4%)	14/653 (2·1%)	0·4	4·7	
**■ Total**	**213/ 8299 (2·6%)**	**201/ 8328 (2·4%)**	**−8·2**	**75·5**	**0·897 (0·716 − 1·124)** 2p > 0·1; NS

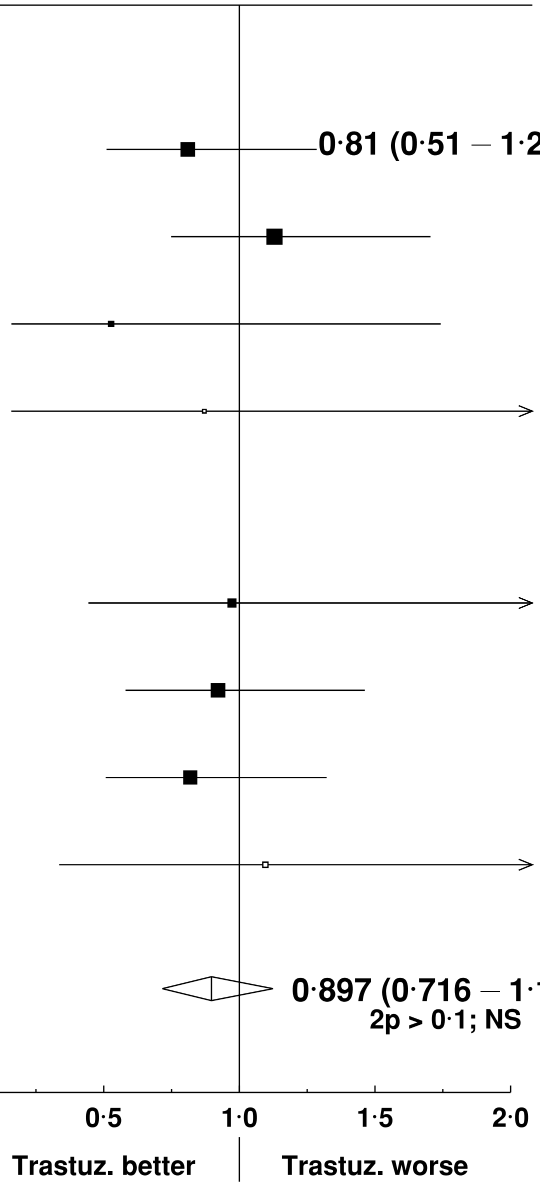

99% or 95% confidence intervals

Treatment effect 2p > 0·1; NS

P26-29: Published adverse event data

Year code	Trial name<br>Comparison	N randomised	Non-fatal cardiac events	Second primary cancers by site	Causes of death	Follow-up (median)
00C	**NSABP B-31**<br>4**AC** q3; (4**P**175q3 ± 12mths **Tr** q1)	2119	Trastuzumab vs not:<br>Overall CHF rates: 3.8% vs 1.3%	Trastuzumab vs not:<br>As first disease-free survival event: 1.0% (11/1055) vs 2.7% (28/1046)	Trastuzumab vs not:<br>Death without evidence of disease: 0.8% (8/1055) vs 0.9% (9/1046)	3.9 years
00D, 00~	**NCCTG N9831**<br>4**AC** q3; (12**P**80 q1 ± 12mths Tr q1) **	3505<br>(328 + 3177)	Trastuzumab vs not:<br>Overall CHF rates: 2.3% vs 0.9%	Trastuzumab vs not:<br>As first disease-free survival event: 1.2% (12/973) vs 1.5% (15/971)	Trastuzumab vs not:<br>Death without evidence of disease: 0.7% (7/973) vs 0.5% (5/971)	3.9 years
	**NCCTG N9831**<br>4**AC** q3; ((12P80 q1 + 12mths **Tr** q1) vs (12**P**80q1;12mths **Tr**q1)) (Concurrent.vs Sequential))	3505<br>(328 + 3177)	Concurrent vs sequential taxane and Trz:<br>Cardiac events causing treatment discontinuation:<br>- asymptomatic drop in LVEF: 7.0% (66/949) vs 4.5% (43/954) *(p=0.022)*;<br>- congestive heart failure: 2.2% (21) vs 1.7% (16) *(p=0.40)*	Concurrent vs sequential taxane and Trz:<br>Second primary disease causing treatment discontinuation: 0.1% (1/949) vs 0.1% (1/954)<br>Second primary disease as the first disease-free survival event: 2.8% (27/949) vs 2.5% (24/954)	Concurrent vs sequential taxane and Trz:<br>Causes of death:<br>- febrile neutropenia/sepsis: 0 (0/949) vs 0 (0/954);<br>- pneumonia: 0 (0) vs 0.2% (2);<br>- pulmonary embolism: 0.1% (1) vs 0.1% (1);<br>- respiratory distress syndrome: 0 (0) vs 0.1% (1)<br>- respiratory failure: 0.1% (1) vs 0 (0);<br>- cardiac arrest: 0.1% (1) vs 0.1% (1/954);<br>- indwelling catheter placed on pericardia cavity causing cardiac death: 0 (0/949) vs 0.1% (1/954);<br>- automobile accident: 0 (0/949) vs 0.1% (1/954).	6 years
00C, 00D, 00~ (joint analysis)	Joint analysis of **NSABP B-31** and the concurrent Trz arm in **NCCTG N9831** vs the no Trz arms **<br>4**AC** q3; (4**P**175q3 ± 12mths **Tr** q1)<br>or<br>4**AC** q3; (12**P**80 q1 ± 12mths **Tr** q1)	2119 + 3505	(Reported on discontinuation of Trz due to adverse cardiac effect, at a median follow-up of 2 years.)	CT ± concurrent Trz:<br>• Total second primary cancers: 3.3% (66/2028) vs 3.7% (74/2018)<br>• Second primary that caused death:<br>- Solid tumour: 0.7% (15/2028) vs 0.7% (15/2018);<br>- Haematologic malignancy: 0.1% (2/2028) vs 0.3% (7/2018)	• Death without progressive disease, second primary, or contralateral breast disease: 1.9% (38/2028) vs 1.5% (31/2018);<br>• Breast cancer: 10.4% (210/2028) vs 16.9% (341/2018);<br>• Second primary cancer:<br>- Solid tumour: 0.7% (15/2028) vs 0.7% 0.(15/2018);<br>- Haematologic malignancy: 0.1% (2/2028) vs 0.3% (7/2018)<br>• Cardiac conditions:<br>- CHF: 0.1% (3/2028) vs 0.0 (2/2018);<br>- Cardiac arrest: 0.1% (3/2028) vs 0.0 (1/2018);<br>- Cardiomyopathy: 0.05% (1/2028) vs 0 (0/2018)<br>- MI: 0.05% (1/2028) vs 0 (0/2018)<br>• Unspecified cardiac: 0.05% (1/2028) vs 0 (0/2018)<br>• Sepsis/septicaemia: 0.05% (1/2028) vs 0.4% (8/2018)<br>• Other cause: 0.7% (14/2028) vs 1.0% (21/2018);<br>• Unknown cause: 1.7% (35/2028) vs 1.1% (23/2018)	8.4 years for the Trz arm and 8.3 years for the control arm, with 4% in the Trz arm and 8.2% in the control arm followed for <5 years.
00E2	**FinHer**<br>((3**D**100q3 v 9**Vin**25q1) ± 9**Tr** q1); 3**F**600**E**60**C**600	232	CT ± concurrent Trz:<br>- LVEF decrease > 20% from baseline after completion of CT: 6.8% (7/115) vs 10.5% (10/116);<br>- Symptomatic heart failure: 0.9% (1/115) vs 1.7% (2/116);<br>- MI: no events in either arm.	No details	CT ± concurrent Trz<br>- Breast cancer: 8.7% (10/115) vs 17.2% (20/116);<br>- Suicide: 0.9% (1) vs 0.0 (0);<br>- CAD: 0.0 (0) vs 0.0 (0);<br>- ALS: 0.0 vs 0.9% (1);<br>- Colon cancer: 0.0 vs 0.0;<br>- Not known: 0.9% (1) vs 0.0 (0)	62 mths (range 2 to 81)

01B	**HERA**<br>Chemo; **Tr** q3 (12 or 24mths) vs not	5102	CT ± sequential Trz:<br>- Primary cardiac event‡: 1% (2-year Trz) vs 1% (1-year) vs 0.1%<br>- Secondary‡: 7.3% (2-year Trz) vs 4.4% (1-year Trz) vs 0.9%	Second non-breast malignancy, 2-year Trz vs 1-year Trz vs observation:<br>3.5% vs 2.8% vs 2.4%	Death without evidence of disease, 2-year Trz vs 1-year Trz vs observation: 1.2% vs 0.8% vs 1.2%.	11 years<br>(safety population)
			1-year Trz vs observation:<br>- Severe congestive heart failure (NYHA III and IV): 0.6% vs 0, p<0.001<br>- Symptomatic CHF: 2% vs 0.1%, p<0.001<br>- Confirmed significant LVEF drop: 3% vs 0.5%, p<0.001<br>- Cardiac problems resulting in Trz discontinuation: 4% vs NA, p<0.0001	In the 1-year Trz group: 0.06% (1/1688) fatal pulmonary carcinomatous lymphangitis, and 0.06% (1/1688) fatal non-breast malignant disease which caused intestinal obstruction.	1-year Trz vs observation:<br>- Fatal adverse event: 0.5% vs 0.2%, p=0.160. In the Trz arm: one each for cerebral haemorrhage, cerebrovascular accident, sudden death, appendicitis, intestinal obstruction, unknown cause of death after a road accident, and pulmonary carcinomatous lymphangitis, respectively; two unknown. In the observation arm: one each for cardiac failure, suicide, and unknown cause of death respectively.<br>- Cardiac death: 0 vs 0.1%, p=1.000	23.5 mths
01E2+4	**PACS 04**<br>6(**F**500**E**100**C**500 v **E**75**D**75); 12mths **Tr** q3 vs not	528	LVEF decline<br>- Severe: 11.1% (29/260) vs 2.6% (7/268)<br>- Moderate: 3.1% (8) vs 1.6% (4)<br>- Mild: 21.2% (55) vs 10.1% (27)<br>(18% of women in the Trz group discontinued treatment due to cardiac events of any grade)	1.2% (3/260) vs 0.4% (1/268).<br>Of the women assigned to the Trz group and did not receive Trz, one was due to second cancer.	• Breast cancer: 7.7% (20/260) vs 6.7% (18/268);<br>• Other: 1.2% (3/260) vs 0.4% (1/268)<br>• No death due to cardiac causes.	47 mths
01M	**BCIRG 006**<br>4**AC** q3; (4**D**100q3 ± 12mths **Tr** q1)<br>vs (6**D**75**CisP**75 q3 + 18**Tr** q1) ; 11**Tr** q3	3222	AC-TH vs TCH vs AC-T:<br>- NYHA III/IV congestive heart failure: 2.0% (21/1074) vs 0.4% (4/1075) vs 0.7% (7/1073): p<0.001 for AC-TH vs TCH. *(p=0.008 for AC-TH vs AC-T; p=0.363 for TCH vs AC-T)*<br>- >10% relative reduction in LVEF: 18.6% (194/1074) vs 9.4% (97/1075) vs 11.2% (114/1073), p<0.001 for AC-TH vs TCH. *p=0.000 for AC-TH vs AC-T; p=0.213 for TCH vs AC-T)*	AC-TH vs TCH vs AC-T:<br>Leukaemia: 0.1% (1/1068) vs 0.1% (1/1056) vs 0.6% (6/1050): p=1.0 for AC-TH vs TCH. *(p=0.056 for AC-TH vs AC-T; p=0.057 for TCH vs AC-T)*	There was no cardiac-related death.<br>Five of the eight patients with leukaemia had died (unclear from which treatment group).	65 mths
02A1+2	**NOAH**<br>neo(3**A**60**P**150q3; 4**P**175q3;3**CMF**q4) ± 12mths**Tr** q3	235	All grades of cardiac events: 11%(13/115) vs 11% (12/113)<br>- Angina pectoris: 4% (5/115) vs 4% (5/113)<br>- Arrhythmia: 1% (1) vs 0 (0)<br>- Bradycardia: 0 (0/115) vs 1% (1/113)<br>- Left ventricular dysfunction: 2% (2) vs 0 (0)<br>- Palpitations: 3% (3) vs 2%% (2)<br>- Sinus tachycardia: 0 (0) vs 1% (1)<br>- Tachycardia: 3% (4) vs 3% (4)<br>Left ventricular dysfunction grade 3/4: 2% (2) vs 0 (0).<br>LVEF worst value during treatment and follow-up by NCI-CTC grade: ≠<br>- Grade 0: 73% (84/115) vs 83% (94/113)<br>- Grade 1: 23% (26/115) vs 16% (18/113)<br>- Grade 2: 2% (2/115) vs 1% (1/113)<br>- Grade 3: 2% (2/115) vs 0 (0/113).<br>Drug-related cardiovascular adverse events:<br>- Grade 2 lymphostasis: 1% (1/115) vs 0 (0/113)<br>- Grade 2 lymphoedema: 1% (1/115) vs 0 (0/113)	Not reported	Not reported	5.4 years

			- Grade 2 thrombosis: 0 (0/115) vs 1 (1/113)<br>- Grade 2 lymphoedema: 1% (1/115) vs 0 (0/113)<br>Discontinued treatment due to cardia event: 1% (1/115) vs 0 (0/113)			

Abbreviations: AC= doxorubicin + cyclophosphamide; AC-T=doxorubicin + cyclophosphamide then docetaxel; AC-TH=doxorubicin + cyclophosphamide then (docetaxel + trastuzumab); ALS=amyotrophic lateral sclerosis; BC=breast cancer; CAD=coronary artery disease; Chemo=chemotherapy; CHF=congestive heart failure; CI=confidence interval; CisP= Cis-Platinum; CMF= cyclophosphamide, methotrexate, fluorouracil; CT=chemotherapy; D=doxorubicin; E=4-epi-doxorubicin; F=5-fluoro-uracil; LVEF= left ventricular ejection fraction; HER2=human epidermal growth factor receptor 2; MI= myocardial infarction; mths=months; NA=not applicable; neo=neoadjuvant; NYHA=New York Heart Association; OR=odds ratio; P=paclitaxel; q1= weekly; q3=3 weekly; TCH=docetaxel + Cis-Platinum + trastuzumab; Tr or Trz=trastuzumab; Vin= vinorelbine.

Note: statistical significant test results were not reported in the publications unless otherwise stated in this table. P-values in *Italic* are from Mantel-Haenszel tests not publications.

** For the N9831 trial patients in the sequential arm (AC followed by paclitaxel followed by trastuzumab) were not included.

ǂ Primary cardiac events: NYHA class III or IV, confirmed by a cardiologist, and a significant LVEF drop of >10% points from baseline and to an absolute LVEF below 50%, or cardiac death. Secondary cardiac events: NYHA class I/II with a significant LVEF drop of >10% points below baseline and to an absolute LVEF below 50% confirmed by repeat assessment. For the observation group: the events were before treatment for recurrent disease and before treatments crossover.

≠ NCI-CTC=National Cancer Institute common toxicity criteria. Grade 1 refers to asymptomatic reduction of 10% or more, but less than 20% of baseline. Grade refers to asymptomatic reduction to lower limit of normal or 20% or more of baseline. Grade 3 refers to congestive heart failure, responsive to treatment.

References for the above adverse events data

Year code	Trial name	Reference
00C	NSABP B-31	Perez EA, Romond EH, Suman VJ, et al. Four-year follow-up of trastuzumab plus adjuvant chemotherapy for operable human epidermal growth factor receptor 2–positive breast cancer: joint analysis of data from NCCTG N9831 and NSABP B-31. *J Clin Oncol* 2011; 29: 3366.
00D, 00~	NCCTG N9831<br>(Trz vs not, 3.9 years of follow-up)	Perez EA, Romond EH, Suman VJ, et al. Four-year follow-up of trastuzumab plus adjuvant chemotherapy for operable human epidermal growth factor receptor 2–positive breast cancer: joint analysis of data from NCCTG N9831 and NSABP B-31. *J Clin Oncol* 2011; 29: 3366.
	NCCTG N9831<br>(concurrent vs sequential Trz, 6 years of follow-up)	Perez EA, Suman VJ, Davidson NE, Gralow JR, Kaufman PA, Visscher DW, Chen B, Ingle JN, Dakhil SR, Zujewski J, Moreno-Aspitia A. Sequential versus concurrent trastuzumab in adjuvant chemotherapy for breast cancer. *J Clin Oncol* 2011;29:4491.
00C, and 00D, 00~ (Joint analysis)	NSABP B-31 and NCCTG N9831 (Joint analysis)	Perez EA, Romond EH, Suman VJ, et al. Trastuzumab plus adjuvant chemotherapy for human epidermal growth factor receptor 2–positive breast cancer: planned joint analysis of overall survival from NSABP B-31 and NCCTG N9831. *J Clin Oncol* 2014; 32: 3744-52.
00E2	FinHer	Joensuu H, Bono P, Kataja V, et al. Fluorouracil, epirubicin, and cyclophosphamide with either docetaxel or vinorelbine, with or without trastuzumab, as adjuvant treatments of breast cancer: final results of the FinHer Trial. *J Clin Oncol* 2009; 27: 5685−92
01B	HERA	Cameron D, Piccart-Gebhart MJ, Gelber RD, et al. 11 years' follow-up of trastuzumab after adjuvant chemotherapy in HER2-positive early breast cancer: final analysis of the HERceptin Adjuvant (HERA) trial. *Lancet* 2017; 389: 1195–205.<br>Smith I, Procter M, Gelber RD, et al. 2-year follow-up of trastuzumab after adjuvant chemotherapy in HER2-positive breast cancer: a randomised controlled trial. *Lancet* 2007; 369: 29-36.
01E2+4	PACS 04	Spielmann M, Roché H, Delozier T, et al. Trastuzumab for patients with axillary-node–positive breast cancer: results of the FNCLCC-PACS 04 trial. *J Clin Oncol* 2009; 27: 6129-34.
01M	BCIRG 006	Slamon D, Eiermann W, Robert N, et al. Adjuvant trastuzumab in HER2-positive breast cancer. *N Engl J Med* 2011; 365: 1273-83.
02A1+2	NOAH	Gianni L, Eiermann W, Semiglazov V, et al. Neoadjuvant chemotherapy with trastuzumab followed by adjuvant trastuzumab versus neoadjuvant chemotherapy alone, in patients with HER2-positive locally advanced breast cancer (the NOAH trial): a randomised controlled superiority trial with a parallel HER2-negative cohort. *Lancet* 2010; 375: 377-84.<br>Gianni L, Eiermann W, Semiglazov V, et al. Neoadjuvant and adjuvant trastuzumab in patients with HER2-positive locally advanced breast cancer (NOAH): follow-up of a randomised controlled superiority trial with a parallel HER2-negative cohort. *Lancet Oncol* 2014; 15: 640-7.

P32: Published* trial results of any recurrence from trials comparing longer versus shorter trastuzumab duration

Category	Events/Women: Allocated Longer	Events/Women: Allocated shorter	Longer events: Logrank O−E	Longer events: Variance of O−E	Ratio (& CI)
**(a) Trials of 6–12 months vs 9–12 weeks of trastuzumab**					
99(ECOG EST2198	13/57 (22·8%)	16/63 (25·4%)	−0·8	5·5	
06W INC Colombia	*(160 patients)*		*(no data)*		
08F SOLD	81/1089 (7·4%)	105/1085 (9·7%)	−12·0	42·5	0·75 (0·56 – 1·02)
**(a) subtotal with data §**	**94/ 1146 (8·2%)**	**121/ 1148 (10·5%)**	**−12·8**	**48·0**	**0·77 (0·58 – 1·02)** 2p = 0·07
**(b) Trials of 1 year vs 6 months of trastuzumab**					
04S HORG	17/241 (7·1%)	28/240 (11·7%)	−5·6	10·2	0·58 (0·31 – 1·07)
06R PHARE	213/1690 (12·6%)	247/1690 (14·6%)	−17·0	99·4	0·84 (0·69 – 1·03)
07W Persephone	218/2045 (10·7%)	234/2043 (11·5%)	−8·1	101·0	0·92 (0·76 – 1·12)
**(b) subtotal**	**448/ 3976 (11·3%)**	**509/ 3973 (12·8%)**	**−30·6**	**210·6**	**0·86 (0·76 – 0·99)** 2p = 0·03
**(c) Trials of 2 years vs 1 year of trastuzumab**					
01B HERA/BIG 01–01	455/1700 (26·8%)	452/1702 (26·6%)	−0·8	210·1	1·00 (0·87 – 1·14)
**Total known**	**997/ 6822 (14·6%)**	**1082/ 6823 (15·9%)**	**−44·2**	**468·7**	**0·910 (0·831 – 0·996)** 2p = 0·04

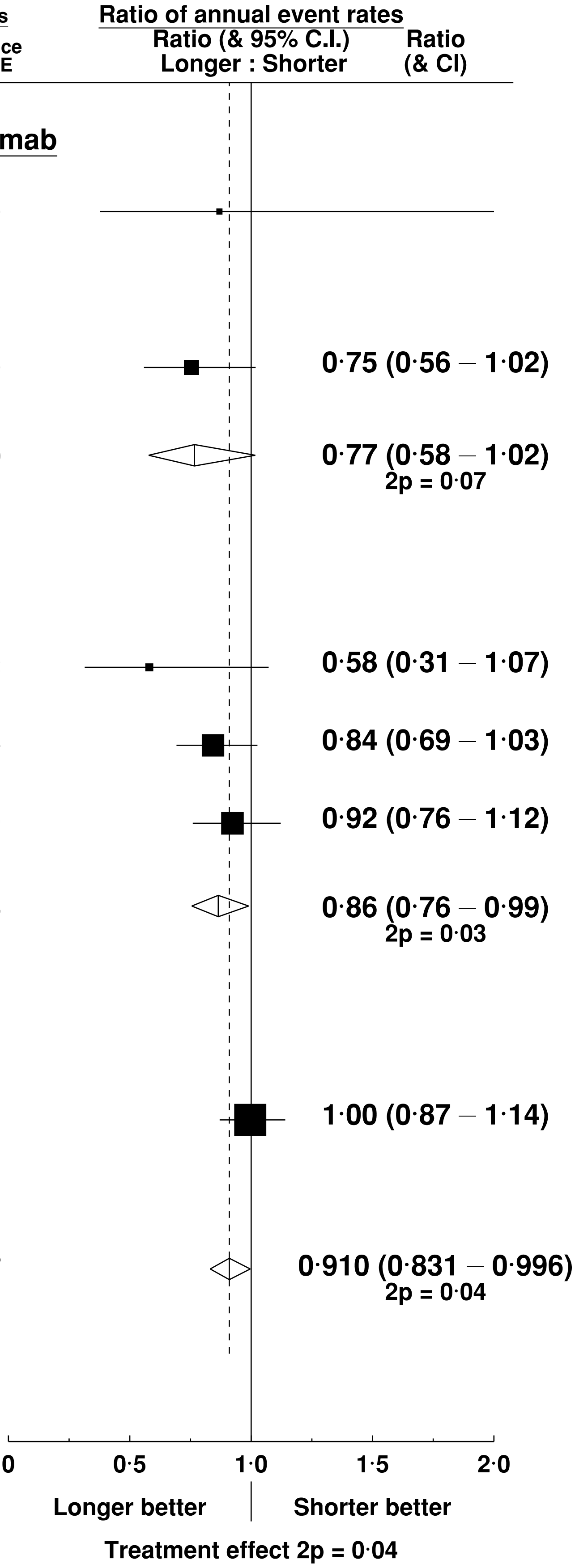

Heterogeneity between 3 subtotals: χ^2_2 = 3·7; p > 0·1; NS

Heterogeneity within subtotals: χ^2_3 = 2·2; p > 0·1; NS

Heterogeneity between 6 categories: χ^2_5 = 5·9; p > 0·1; NS

*Individual patient data used for HERA

Statistical Analysis Plan for the EBCTCG meta-analysis of trials of trastuzumab

May 2018

This meta-analysis aims to assess the benefits and risks of adding trastuzumab in the adjuvant therapy of women with HER-2 positive early breast cancer. It will include all trials where one group received trastuzumab and at least one other did not, providing all other aspects of treatment were the same in both groups. See Appendix 1 for details of relevant trials identified.

Trials comparing trastuzumab (of any duration) versus a no trastuzumab control:

a) Trials of post-operative trastuzumab vs no trastuzumab
b) Trials of pre- plus post-operative trastuzumab vs no trastuzumab

Analysis populations

Data will be sought from all relevant, randomised trials starting in, or before, 2010, irrespective of study size or primary outcome measures. The data to be requested are detailed in appendix 2.

Data checking

The usual EBCTCG quality assurance checks for range, consistency and balance between randomisation arms will be undertaken prior to analysis. In addition, to validate the death without distant recurrence analyses (see below), the quality of second recurrence and cause of death information for each trial will be explored and, if appropriate for particular trials, death without distant recurrence will be estimated as overall mortality rates minus mortality rates without any recurrence rather than without distant recurrence. The dependence of deaths without recorded recurrence on TN status will also be investigated with any association used to estimate what proportion might actually have been from breast cancer. Checks will be undertaken to compare the incidence of death without recurrence and of second cancers (overall and by site) by age group in each trial to characterise whether these might include miscoded breast cancer events.

Analyses

Primary analyses will be by Intention-to-Treat (ITT), including all randomised patients irrespective of treatment compliance. Sensitivity analyses will also be undertaken to investigate the potential impact of non-compliance on treatment efficacy.

Primary outcomes

The main endpoint definitions and methods are those used in previous EBCTCG reports, but with minor amendments reflecting the potential impact of HER-directed therapy.

1. **Breast cancer recurrence:** includes distant recurrence, locoregional recurrence and new second primary breast cancer (ipsilateral or contralateral), and the definitions of these will be as in each trial.
2. **Breast cancer mortality:** Information about mortality rates without recurrence will be subtracted from information about overall mortality rates ('logrank subtraction' as in previous EBCTCG reports). This information will be used to construct Kaplan-Meier graphs that estimate breast cancer mortality (i.e. the pattern of mortality that would have been seen if it had been possible to avoid all deaths before or after recurrence from causes other than breast cancer).
3. **Death without recurrence:** i.e. without the secretariat having any record of recurrence. Because the quality of the recurrence data in the HER-directed therapy trials is likely to be

reasonably good, deaths from a wholly unknown cause without record of recurrence will be treated as deaths from an unknown cause that was not breast cancer.

4. **All-cause mortality**

Exploratory endpoints

1. **Time to first distant recurrence:** includes distant recurrence, death from breast cancer without recorded distant recurrence, and ignores any prior loco-regional or contralateral recurrence. Information about the site(s) of first distant recurrence will be collected to allow two specific additional exploratory analyses to be performed.
    a. **Time to CNS/brain recurrence as first distant site:** Time to CNS recurrence, with or without any concomitant distant recurrence, and ignoring any prior loco-regional or contralateral recurrence.
    b. **Time to recurrence outside CNS as first distant site:** Time to first distant recurrence not including CNS and ignoring any prior loco-regional or contralateral recurrence.
2. **Death without distant recurrence:** (i.e. without any record of distant recurrence). Deaths from a wholly unknown cause without record of distant recurrence will be treated as deaths from an unknown cause that was not breast cancer.
3. **Death after distant recurrence** Information about mortality rates without distant recurrence will be subtracted from information about overall mortality rate. This provides an alternative estimate of breast cancer mortality that ignores isolated locoregional recurrence or new primary breast cancers (i.e. the pattern of mortality that would have been seen if it had been possible to avoid all deaths before or after distant recurrence from causes other than breast cancer).
4. **Time to loco-regional recurrence as first event**: includes ipsilateral breast, chest wall and locoregional lymph nodes (axilla and SCF).
5. **Time to new second primary breast cancer**
6. **Non-fatal cardiovascular events:** The incidence of non-fatal cardiovascular events (eg MI, stroke, congestive heart failure) requiring hospitalisation will be compared.
7. **Safety –** Data on the incidence and site of second cancers before any recurrence of breast cancer and causes of death without recurrence will be sought and analysed.

Subgroup analyses:

Exploratory subgroup analyses will be undertaken but, given the well-known hazards of subgroup analysis, will be interpreted appropriately cautiously.

Investigation of potential interactions between tumour or patient characteristics and treatment efficacy will also be undertaken with recurrence as primary outcome. Unless treatment efficacy is shown to vary with treatment duration or scheduling (pre or post-operative) of treatment, the analyses below will include all trials in cohorts 1a and 1b. Similarly, if the above analyses demonstrate that trastuzumab reduces distant metastases but not local recurrence or contralateral breast cancers, then the subgroup analyses to compare efficacy within subgroups would exclude the unrelated breast cancer events (ie local and/or contralateral) from the outcome measure.

Subgroup analyses of Recurrence Forest plots for subgroup analyses by:

- Site of recurrence (distant metastasis, local recurrence or contralateral breast cancer)
- Age (<45, 45-54, 55-69, ≥70, unknown),

- Premenopausal or <45 if unknown, perimenopausal or 45-54 if unknown, postmenopausal (natural or induced) or 55+ if unknown
- ER status (ER-poor, ER+)
- ER/PR status (ER+/PR- vs ER+/PR+)
- HER2:CEP17 ratio (<2.0,2.0 to <5.0, 5.0 to <7.5, 7.5 to <10.0, ≥10.0)
- Nodal status (negative, N1-3, N4+, N unknown)
- Tumour stage (T1, T2, T3/T4, T stage unknown);
- Histological grade (1, 2, 3, unknown),
- Proliferation index (%Ki-67: 0-9, 10-19, 20+, unknown)
- Tumour histology (ductal, lobular, other, unknown)
- Presence/ absence of chemotherapy
- Trastuzumab given synchronously/ sequentially with chemotherapy
- Presence/ absence endocrine therapy
- Period of follow-up years 0-1, 2-4, 5-9, and 10+ after randomisation.

Appendix 1. Trials of HER-directed therapies so far identified

Trial (Yr) Code	Trial name	Comparison	Size	
**1a) Trials of postoperative trastuzumab**				
4757/58 (00C)	NSABP B-31	ACP(T) ± trastuzumab (2mg/kg) qwk x 52	2130	Data received 2013
4773/77 (11D)	NSABP B-47	DC/ACP ± trastuzumab (6mg/kg) q3wk x 12	3260	Not eligible (HER2-low)
7710-14 (00D)	NCCTG N9831	ACP(T/AI) ± trastuzumab (2mg/kg) qwk x 52	3505	Data received 2018
8318 (01D)	CALGB 49808	(Dex)ACP ± trastuzumab (?mg/kg) qwk x 12 preop then ± qwk x 40 postop	Few	Not requested (No FU)
28903 (00E)	FinHER	DFEC/VFEC ± trastuzumab (2mg/kg) qwk x 9	232	Data received 2013
33203/07 (01E)	PACS 04	FEC/ED ± trastuzumab (6mg/kg) q21d until 1yr	528	Data received 2016
33505 (01M)	BCIRG 006	ACD ± trastuzumab (2mg/kg) qwk × 12 then (6mg/kg) q21d until 1yr	3222	Data received 2017
35401-02 (01B)	HERA BIG 01-01	pre/postop chemotherapy ± trastuzumab (6mg/kg) q21d x 1yr/2yr	5102	Data received 2018
40201 (03^)	Heraklion, Greece	± trastuzumab (6mg/kg) q3wk x 6	95	Not eligible (HER2-neg)
**1b) Trials of pre plus postoperative trastuzumab vs control**				
8318 (01D)	CALGB 49808	(Dex)ACP ± trastuzumab (?mg/kg) qwk x 12 preop then ± qwk x 40 postop	Few	Not eligible (No FU)
34504/07 (02A)	NOAH	AP;CMF ± trastuzumab (6mg/kg) q21d x 11 preop then q21d x 7 postop	235	Data received 2012

Appendix 2 –

Either using the codes we suggest below or using your own codes, please extract from your dataset the variables that correspond most closely to the items listed below and send them to us. Please provide one record for each woman ever randomised (including any woman who was randomised and then was later categorised as ineligible, withdrawn, unevaluable, lost or "protocol deviant" – but, please tell us in question 9 which patients your group's preferred analyses would exclude, and why).

For trials where a dataset has previously been sent to the EBCTCG it is probably easiest and most reliable to update by re-sending all variables. If, however, this would cause difficulties then you can send only the additional variables; let us know if you want a file of the data you previously sent and we will provide it.

If any variable is not available or not applicable, please omit it and send only the remaining variables. If you have any of the requested variables in your records in a form that would require substantial additional work to supply (e.g. computerisation, or manual coding), please feel free to omit them for now, but in your cover document please tell us of their existence.

- Please send your data in a separate Excel spreadsheet for each separate trial, if possible.
- Please send a cover document giving all your coding conventions (including your format for dates).
- Please send your data to: bc.overview@ndph.ox.ac.uk with your research group's name (and/or the EBCTCG number for your research group) and your group's name for the trial in the subject line.

If you have any questions about this data request, please contact the EBCTCG secretariat on bc.overview@ndph.ox.ac.uk (tel: +44-1865-743852). All data supplied to the secretariat will be held securely and treated confidentially, in accordance with the EBCTCG Data Policy (available at: https://www.ctsu.ox.ac.uk/research/ebctcg) and the UK Medical Research Council data security policies.

CORE VARIABLES - BASELINE (Q1-27)

A) Randomisation and patient characteristics (Q1-9)

1. **Your patient identifier** *(preferably specifying uniquely which trial as well as which patient)*
2. **Date of randomisation** *(specify your format for dates [in your covering document])*
3. **Allocated treatment** *(specify your codes)*
4. **Age** at randomisation *(years)* **NB Here & everywhere else, leaving an item Blank means Not Known**
5. **Height** at randomisation *(m)*
6. **Weight** at randomisation *(kg)*
7. **Menopausal status** at randomisation *(1=pre-, 2=peri-, 3=postmenopausal with intact ovaries & uterus, 4=ovarian ablation, 5=hysterectomy, 6=both [ie, 4 and 5])*
8. **Did chemotherapy cause apparently permanent cessation of menses?** *(1=no/not applicable, 2=yes)*
9. **Would your group's preferred analyses exclude this patient?** NB A few trial patients may be randomised in error, otherwise ineligible, lost with no follow-up, unevaluable or withdraw consent. *(1=no known reason for exclusion, 2=yes [specify main reason(s) for preferring exclusion, if known])*

B) Surgical details (Q10-11; or, define and use your own codes)

10. **Breast surgery** *(1=none, 2=only lumpectomy or wide local excision, 3=quadrantectomy or sector resection, 4=partial mastectomy, 5=simple or total mastectomy, 6=radical mastectomy)*
11. **Axillary surgery** *(1=none, 2=sentinel node biopsy only, 3=axillary sampling, 4=surgical clearance of less than levels I & II, 5=full clearance of axillary levels I & II, 6=clearance of more than levels I & II, 7=axillary clearance, but levels cleared unspecified)*

C) Nodal status (Q12-13; or, use your own codes [eg, TNM])

12. **Sentinel node biopsy** *(1=not done, 2=done and negative for cancer, 3= isolated tumour cells [≤0.2 mm], 4=micrometastasis (0.2 to 2mm), 5=macroscopic nodal deposit [>2 mm], 6=positive, size unknown)*
13. **Axillary status** *(specify codes, or: 1=pN- histologically, 2=N- other/unknown method, 3=1-3 positive nodes, 4=4-9 [or 4+] positive, 5=10+ positive, 6=N+ histologically, unknown number, 7=N+ other/unknown method)*

D) Tumour characteristics (Q14-18; or, use your own codes [eg, TNM])

14. **Method first detected** *(1=mammographic screening, 2=incidental, 3=symptomatic, 4=other)*
15. **Laterality** *(1=left, 2=right, 3=bilateral)*
16. **Pathological grade** *(1=well differentiated, 2=moderately,3=poorly)*
17. **Histological type** *(1=invasive, not otherwise specified, 2=ductal, 3=lobular, 4=other, 5=mixed)*
18. **Tumour diameter:** largest diameter of excised primary *(mm)*

E) Receptor status (Q19-27; or, use your own codes)

Note: In trials with some neo-adjuvant systemic therapy give receptor status at randomisation, i.e. prior to any neo-adjuvant therapy

19. **Summary of Estrogen Receptor (ER) status of primary tumour** *(1=ER-poor, 2=ER+, 3=ER++ [define in cover document, unless ER-poor is <10 fm/mg and ER++ is ER definitely ≥100 fm/mg])*
20. **Quantitative ER measurement** *(measured in central/reference lab if possible, otherwise best available)*
21. **Units for ER** *(1=fm/mg, 2=% +ve by IHC, 3=Allred score [category score], 4=H-score, 9=other [specify]*)

22. **Summary of Progesterone Receptor (PR) status of primary tumour** *(1=PR-poor, 2=PR+, 3=PR++ [define in cover document, unless PR-poor is <10 fm/mg and PR++ is PR definitely ≥100 fm/mg])*
23. **Quantitative PR measurement** *(done in central/reference lab if possible, otherwise best available)*
24. **Units for PR** *(coded as Q21)*

25. **Summary of HER2 status of primary** *(1=negative/normal, 2=positive/over-expressing)*
26. **Quantitative HER2 measurement** *(done in central/reference lab if possible, otherwise best available)*
27. **Units for HER2** *(1=IHC [% staining], 2=IHC score [0, 1+, 2+, 3+], 3=FISH [# copies], 4=FISH [HER2:CEP17 ratio], 5=CISH [# copies], 6=CISH [HER2:CEP17], 9=other [please specify])*

CORE VARIABLES – FOLLOW-UP (Q28-43)

F) Non-compliance before any recurrence (Q28-29; or, use your own codes)

28. Any substantial deviation from trial treatment allocation (before any breast cancer recurrence)?
(1=no, 2=never started, 3=discontinued, 4=switched to opposite trial group, 5=other [specify])

29. Date of first such deviation from allocated treatment *(ignore deviations after recurrence)*

G) Cancer recurrence and second cancers (Q30-40; or, use your own codes)

30. Any recurrence of invasive breast cancer (ie, locoregional, contralateral or distant)?
NB Includes any occurrence of new ipsilateral or contralateral breast cancer *(1=no, 2=yes)*

31. <u>If no:</u> Date patient last known to be free of such recurrence; <u>If yes:</u> Date of first such recurrence

32. Site of first distant recurrence (ie, possibly distant; not just locoregional/contralateral)
(1=no distant recurrence, 2=recurrence, unknown if distant, 3=distant recurrence, unknown/multiple sites, 4=only in distant soft tissue, 5=only in distant nodes, 6=only in bone, 7=only visceral, 8=only in CNS)

33. Date of first distant recurrence NB Locoregional recurrence can precede first distant recurrence

34. Site of first locoregional recurrence *(1=no locoregional recurrence recorded, 2=multiple or unspecified locoregional sites 3=only in breast [new or recurrent invasive cancer] or chest wall, 4=only in axilla, 5=only in other locoregional nodes [eg, supraclavicular or internal mammary])*

35. Date of first locoregional recurrence

36. Contralateral breast cancer? *(1=no, 2=yes: new invasive cancer thought to have arisen during follow-up in the contralateral breast)*

37. Date of first contralateral breast cancer

NB If patient had more than one second malignancy during follow-up, <u>repeat</u> variables 38-40 for each.

38. Site of any second malignancy *[except breast cancer]* during follow-up *(Describe ALL sites. Use and specify your own codes; if you use ICD codes specify revision, eg ICD-9 or ICD-10)*

39. Date of this second malignancy

40. MIGHT this have been a breast cancer metastasis? *(1=no, 2=possibly/not yet certain [eg, possible lung, liver, bone or brain metastasis: please do not report definite breast metastases as second cancers])*

H) Survival (Q41-43)

41. Is patient known to have died? *(1=no, 2=yes)*

42. <u>If no:</u> Date patient last known to be alive; <u>If yes:</u> Date of death

43. <u>If yes:</u> Cause of death *(use and specify your own codes; if you use ICD codes specify which version, eg ICD-9 or ICD-10)*

ADDITIONAL VARIABLES (Q44-61)

I) Additional tumour marker data (Q44-52; or, use your own codes)

Note: If tests of gene expression or special tests of IHC quantitation were done on the excised primary then <u>please send a separate file</u> in your own format with the fully detailed set of results on each individual.

44. Summary of gene-expression status of primary tumour *(1=low risk, 2=intermediate risk, 3=high risk):* NB Please also provide the fully detailed gene expression results for each patient as a separate dataset.

45. Quantitative gene-expression prognostic score *(best available single numerical measure)*

46. Prognostic score used to quantify gene expression profile *(use own code, or: 1=OncotypeDx prognostic score, 2=Mammaprint prognostic score, 9=other [please specify])*

47. Summary of Topo-isomerase II alpha (TOPO2A) status of primary tumour *(1= normal [ie, no gene over-expression or deletion], 2=positive/over-expressing, 3=deleted)*

48. Quantitative TOPO2A measurement *(done in central/reference laboratory if possible)*

49. Units for TOPO2A *(1=IHC [% staining], 2=IHC score [0, 1+, 2+, 3+], 3=FISH [number of copies], 4=FISH [TOPO:CEP17 ratio], 5=CISH [# copies], 6=CISH [TOPO:CEP17], 9=other [please specify])*

50. Summary of Proliferation Index of primary tumour *(1=low, 2=intermediate, 3=high)*

51. Quantitative Proliferation Measure *(best available numerical measure, in central/ ref lab if possible)*

52. Factor measured for Proliferation Index *(1=S-phase fraction [%], 2=thymidine labelling index [%], 3=Ki-67 by IHC [% staining], 9=other [please specify]*)

J) Bone fractures and cardiovascular events (Q53-54; omit if not sought)

Some trial treatments may cause or prevent bone fractures or cardiovascular events. Please describe all such events (eg, hip fracture, spinal fracture, myocardial infarction, stroke, pulmonary embolus, episode of cardiac failure) if, but only if, such events were sought and recorded systematically for the trial.

If more than one such event was recorded, <u>repeat</u> variables 53-54 for each.

53. Nature of event *(use your own codes; if you use ICD codes, specify which version, eg ICD-9 or ICD-10, and if you use CTC Adverse Event codes, please specify version number, eg CTCAE-3 or CTCAE-4)*

54. Date of event

K) Trials with some neo-adjuvant systemic therapy (Q55-59; or, use own codes)

55. Apparent axillary nodal status (clinical, radiological or other) before neo-adjuvant *(1=N-, 2=N+)*

56. Apparent tumour diameter before neo-adjuvant*:* largest diameter *(mm)*

57. Operability before any neo-adjuvant therapy *(blank if not given; define your own codes, or: 1=Breast-conserving surgery feasible, 2=Mastectomy but not BCS feasible, 3=inoperable, 4=uncertain operability)*

58. Breast tumour response after neo-adjuvant *(blank if not given; define your own codes, or: 1=clinically complete response [cCR] & negative pathology, 2=cCR with DCIS, 3=cCR, cancer remaining, 4=cCR with no pathological information, 5=partial response, 6=stable disease, 7=progression [define 5-7])*

59. Axillary response after neo-adjuvant *(coded as Q58)*

L) Trials of extended endocrine therapy (Q60-61)

60. Date endocrine therapy started (or date of surgery if unknown)

61. Date initial endocrine therapy stopped (ignoring any re-introduction after recurrence)
